# Temperature‑, Concentration‑, and Solvent-Dependent *M*/*P* Helicity Switching of Double-Helical Monometallofoldamers with Inversion of Circularly Polarized Luminescence

**DOI:** 10.1021/jacsau.5c01659

**Published:** 2026-01-30

**Authors:** Kotaro Matsumura, Daiki Tauchi, Masashi Hasegawa, Yoshitaka Tsuchido, Hidetoshi Kawai

**Affiliations:** † Department of Chemistry, Faculty of Science, 26413 Tokyo University of Science, 1-3 Kagurazaka, Shinjuku-ku, Tokyo 162-8601, Japan; ‡ Department of Chemistry, Graduate School of Science, 12877 Kitasato University, 1-15-1 Kitasato, Minami-ku, Sagamihara, Kanagawa 252-0373, Japan

**Keywords:** Chiral switching, Circularly polarized luminescence, Double helix, Foldamer, Stimuli-responsive

## Abstract

Fluorescent double-helical monometallofoldamers [Ag­(**1**)_2_]­[PF_6_] were constructed by the mononuclear complexation of two bipyridine strands **1** featuring two L-shaped dibenzopyrrolo­[1,2-*a*]­[1,8]­naphthyridine units at both ends with a Ag­(I) cation. These monometallofoldamers exhibited double-helical/open conformational switching. [(*R*)-Ag­(**1c**)_2_]­[PF_6_] with chiral side chains induced a single-handed helix sense and enabled precise control of *M*/*P* helicity switching in response to a solvent. This complex also exhibited strong fluorescence and circularly polarized luminescence (CPL) inversion switching upon *M*/*P* helicity inversion. For instance, [(*R*)-Ag­(**1c**)_2_]­[PF_6_] exhibited positive CPL in CH_2_Cl_2_ (φ_F_ = 0.69, *g*_lum_ = 1.9 × 10^–3^) and negative CPL in toluene (φ_F_ = 0.79, *g*_lum_ = −2.0 × 10^–3^). This double helix also aggregated in polar solvents, which led to CPL inversion switching induced by temperature- and concentration-dependent aggregation.

## Graphical abstract

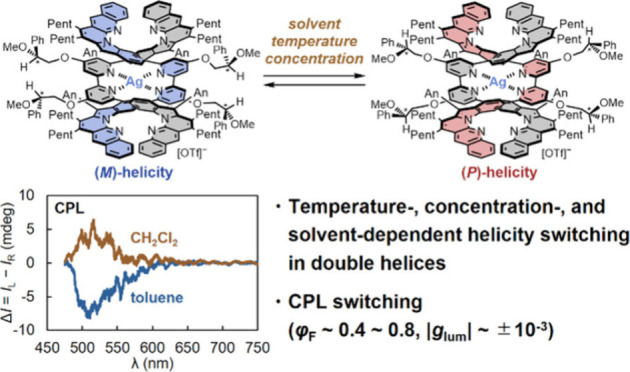

## Introduction

Intricate metallo-supramolecular systems such as helical structures^1–12^ and coordination cages^13–21^ have been constructed through the careful selection and design of metal ions and ligands over the past decade. The stability and geometry of coordination bonds are highly dependent on the choice of metal cation^22–25^ and thus significantly affect the resultant structures and properties of these systems. Simple changes in the metal ions have led to the formation of metallo-supramolecules with different structures and compositions, even with an identical four-coordinate geometry.^23^

Metallofoldamers are dynamic metallo-supramolecular systems that fold into three-dimensional structures through the controlled coordination of metal ions and strands.^8^ Their conformational switching properties have attracted significant attention.^6–11,26–29^ Structural switching without demetallation is a challenge in metallofoldamer systems, because the helical structure is stabilized by multinuclear complexation. This presents a trade-off between structural stability and dynamic switching. Examples of metallofoldamers, which exhibit temperature-dependent structural switching based on dynamic coordination bonds^28^ and helicity inversion switching induced by the coordination of an anion to the metal cation,^29^ have been reported. On the other hand, despite the numerous reports of double-helical complexes,^3,30,31^ double-helical inversion switching has rarely been reported,^32^ and its applications remain unexplored.

As an application of chiral inversion switching,^5,6,29,32–45^ circularly polarized luminescence (CPL) inversion switching has attracted much attention as an efficient method to selectively produce both left- and right-handed circularly polarized light from a single enantiomer.^46–60^ CPL inversion switching has been achieved by diverse mechanisms, including single helix inversion,^46^ conformational changes induced by solvents^47,48^ and light,^49,50^ monomer/excimer switching,^51^ and changes in the aggregation or assembly structure.^52–58^ *D*_2_-symmetric double helices are expected to be superior candidates for efficient CPL materials due to their higher *g*_lum_ values than *C*_2_-symmetric single helices, with a feature resulting from the angle between the transition electric and magnetic dipole moments being 0° or 180°.^61^ However, the realization of CPL inversion switching in these double-helical systems in response to achiral stimuli remains a significant challenge.

We have previously developed short-stranded helical foldamers composed of multiple highly fluorescent L-shaped dibenzopyrrolo­[1,2-*a*]­[1,8]­naphthyridine units^62,63^ linked to various π-conjugated cores.^64–66^ For example, double-helical monometallofoldamers [Zn­(**1**)_2_]­[OTf]_2_ were synthesized by complexing two strands of **1**, each containing two L-shaped units linked by a 2,2′-bipyridine unit, with a Zn­(II) cation. This complex adopted a stable double-helical form but could also unfold in solution to equilibrate with metastable open forms (Scheme 1a).^67^ [Zn­(**1c**)_2_]­[OTf]_2_ with chiral side chains exhibited *M*/*P* helicity inversion via the open forms in response to solvent polarity (Scheme S9), which was accompanied by a significant inversion of Δ*ε* and *g*_abs_. Although these monometallofoldamers were expected to be promising candidates for tunable CPL materials, [Zn­(**1**)_2_]­[OTf]_2_ did not exhibit luminescence, possibly due to nonradiative deactivation via ligand to metal charge transfer (LMCT). On the other hand, [Ag­(**1a**)_2_]­[PF_6_] was prepared by complexing strands **1a** with AgPF_6_ to investigate the conformational isomerism of monometallofoldamers. This complex adopted an open form in the solid state as previously reported.^67,68^ Nevertheless, a detailed investigation is still required to determine whether monometallofoldamer [M­(**1**)_2_]^*n*+^, such as [Ag­(**1**)_2_]­[PF_6_], can adopt a helical structure and undergo *M*/*P* helicity switching.

**Scheme 1:**
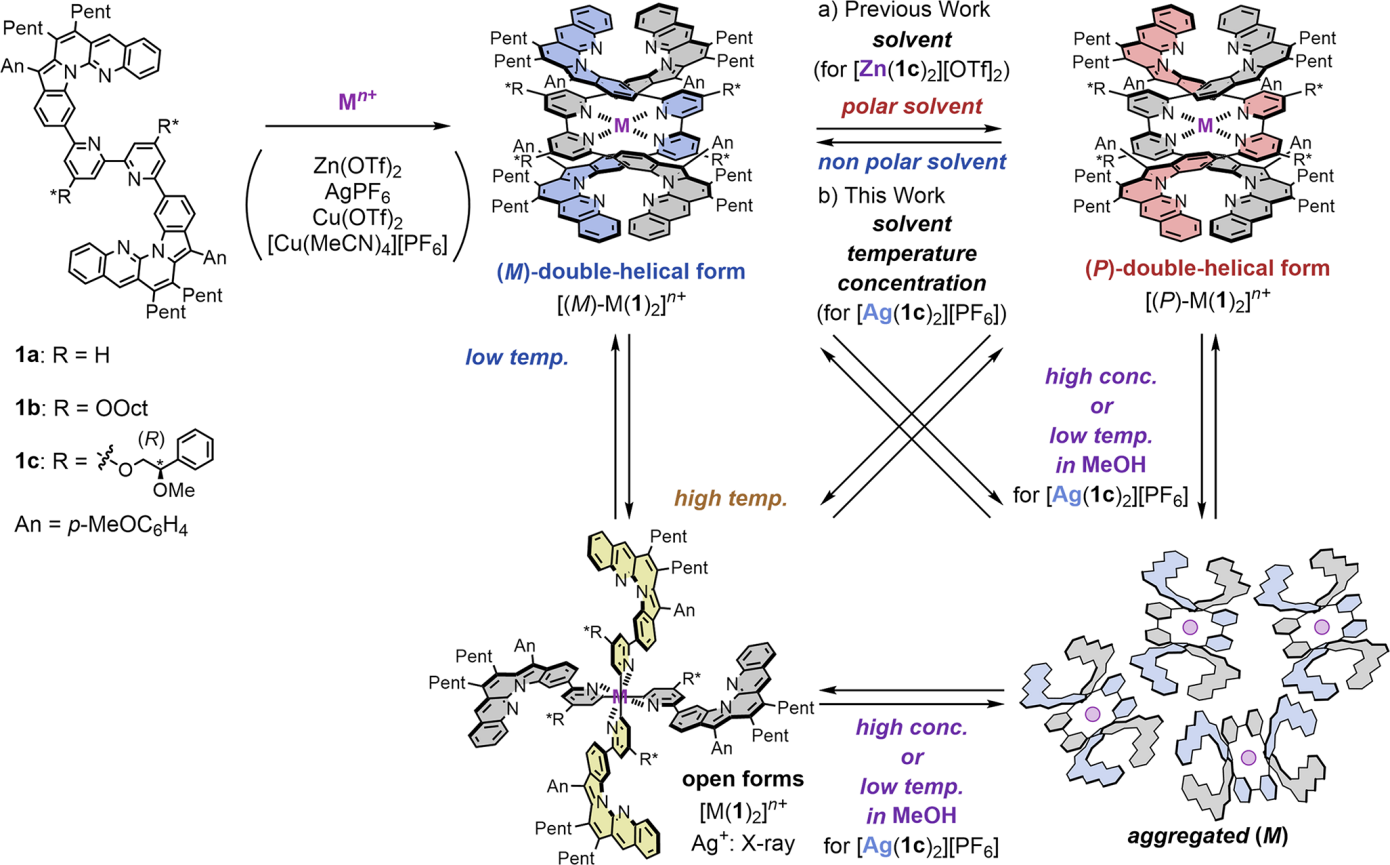
Double-Helical Monometallofoldamers Based on L-Shaped Dibenzopyrrolo­[1,2-*a*]­[1,8]­naphthyridine[Fn s1fn1]

^a^
a) Solvent-responsive *M*/*P* helicity switching in Zn­(II)-based monometallofoldamer [Zn­(**1**)_2_]­[OTf]_2_. b) Temperature-, concentration-, and solvent-responsive *M*/*P* helicity switching in Ag­(I)-based monometallofoldamer [Ag­(**1**)_2_]­[PF_6_].

Here, we investigated metal cations suitable for constructing monometallofoldamers [M­(**1**)_2_]^*n*+^ (M^*n*+^ = Ag^+^, Cu^+^, Cu^2+^, Zn^2+^) and for modulating their chiroptical switching in response to external stimuli. Among these, the Ag­(I)-based monometallofoldamers exhibited efficient fluorescence properties, which indicated their promise as a platform for switchable CPL materials. [Ag­(**1**)_2_]­[PF_6_] exhibited double-helical/open conformational switching and solvent-dependent *M*/*P* helicity inversion in solution. This change was accompanied by a CPL inversion with high fluorescence (|*g*_lum_| = −2.0 × 10^–3^ to 1.9 × 10^–3^, φ_F_ ∼ 0.7). Interestingly, unlike [Zn­(**1c**)_2_]­[OTf]_2_, [Ag­(**1c**)_2_]­[PF_6_] aggregated in polar solvents, which led to CPL inversion switching that was dependent on both the temperature and concentration.

## Results and Discussion

### Complexation and X-ray Structures of [M(1)_2_]^*n* **+**^

First, we investigated the suitability of various metal cations for constructing monometallofoldamers [M­(**1**)_2_]^*n*+^ based on strand **1**. This survey revealed that Ag­(I), Cu­(I), and Cu­(II) cations are suitable in addition to the previously reported Zn­(II)^67^ (for Cu­(I)- and Cu­(II)-based foldamers, see Supporting Information). Notably, the Ag­(I)-based foldamer [Ag­(**1c**)_2_]­[PF_6_] was expected as a promising candidate for a switchable chiroptical material due to the strong fluorescence property, leading us to study their switching properties.

The momometallofoldamers [Ag­(**1**)_2_]­[PF_6_] were prepared by the addition of AgPF_6_ to a solution of achiral strands **1a** and **1b** or chiral strands **1c**. [Ag­(**1a**)_2_]­[PF_6_][Bibr ref67] without substituents on the bipyridyl unit was prepared for X-ray structural analysis (see below). [Ag­(**1b**)_2_]­[PF_6_] with long octyloxy chains was designed to enhance solubility and to characterize in solution. [Ag­(**1c**)_2_]­[PF_6_] with (*R*)-2-methoxy-2-phenylethoxy groups as chiral auxiliaries was prepared specifically to examine the chiroptical switching behavior.

The X-ray structural analysis of [Ag­(**1a**)_2_]­[PF_6_] ^67,68^ revealed that it adopted an open form in the solid phase (Figure 1). In contrast to [Zn­(**1a**)_2_]­[OTf]_2_, which favored the double-helical form,^67^ [Ag­(**1a**)_2_]­[PF_6_] adopted a mononuclear open structure where two bipyridine strands were tetrahedrally coordinated to the silver cation. The four L-shaped units were stacked intermolecularly rather than intramolecularly (Figure 1b). The Ag–N coordination bond lengths were 2.33–2.38 Å (Figure 1c), which are longer than the Zn–N bonds in [Zn­(**1a**)_2_]­[OTf]_2_ (2.02–2.04 Å).^67^ In the open form adopted by the Ag­(I) complex, the two bipyridine units were coordinated to the Ag­(I) cation at an almost right angle (89.3°) (Figure 1d). This is in contrast to the Zn­(II) complex, which adopted the double-helical form with distorted tetrahedral coordination (70.2°).^67^ Such differences in coordination angles and the pitches in the double helices that result from the replacement of Zn­(II) with Ag­(I) are expected to influence the preference for either the open or double-helical form.

**Figure 1:**
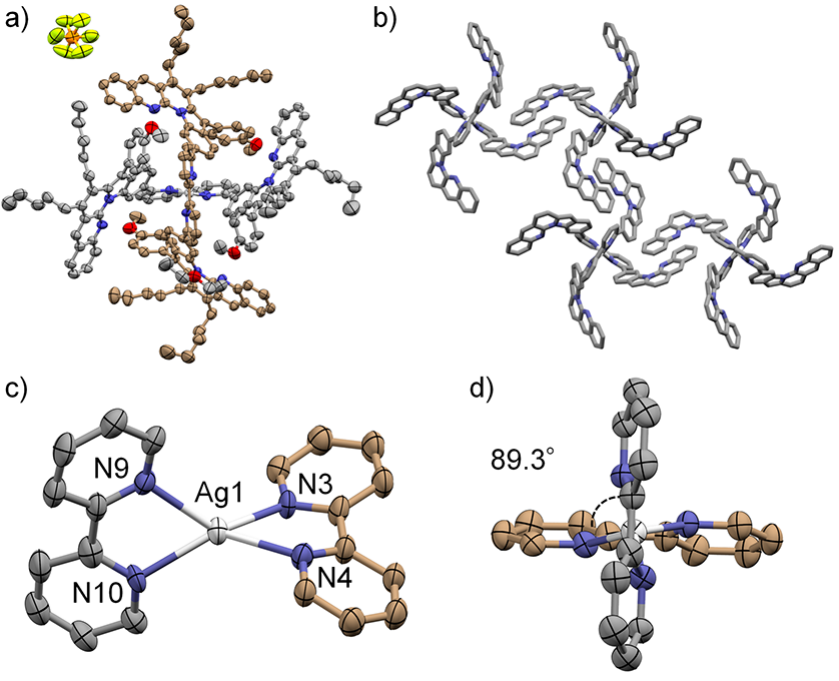
X-ray structure of [Ag­(**1a**)_2_]­[PF_6_] ^67,68^ with thermal ellipsoids at 50% probability; minor disordered parts and hydrogen atoms are omitted for clarity. a) Front view. b) Crystal packing of [Ag­(**1a**)_2_]­[PF_6_] (pentyl groups, anisyl groups, solvents, and counteranions are omitted for clarity). c, d) Coordination geometry of [Ag­(**1a**)_2_]­[PF_6_]; selected atom distances and angles: Ag1–N3 2.333(5) Å, Ag1–N4 2.377(5) Å, Ag1–N9 2.368(5) Å, Ag1–N10 2.347(5) Å, N3–Ag1–N4 70.6(2)°, N4–Ag1–N10 132.6(2)°, N9–Ag1–N10 70.2(2)°, N10–Ag1–N3 132.6(2)°.

### ^1^H NMR Studies of Complex [Ag­(**1b**)_2_]­[PF_6_]

To investigate the conformational preference of the monometallofoldamers [Ag­(**1b**)_2_]­[PF_6_] and [Zn­(**1b**)]­[OTf]_2_, the conformation of [Ag­(**1b**)_2_]­[PF_6_] was evaluated from ^1^H NMR spectra (Figure 2). Two distinct species appeared at 223 K in CD_2_Cl_2_, which indicates slower equilibrium on the NMR time scale. This suggested that [Ag­(**1b**)_2_]­[PF_6_] existed as a mixture of both open and double-helical conformations, which was consistent with the behavior of the double-helical form of [Zn­(**1b**)_2_]­[OTf]_2_.^28^ Furthermore, the large upfield shifts of the bipyridine protons in the major complex compared to those of **1b**, H_a_ (5.53 ppm) and H_b_ (6.15 ppm), suggested π–π stacking between the two L-shaped units and the bipyridine unit within the double-helical form of [Ag­(**1b**)_2_]­[PF_6_] (Figure 2d). In contrast, another chemical species became predominant at 283 K, which was tentatively assigned to the open form, as observed in the X-ray structure of [Ag­(**1a**)_2_]­[PF_6_] (Figure 2f). However, the significantly broad peaks suggested an equilibrium mixture including various open forms with one or more L-shaped units facing outward (Figure S13).

**Figure 2:**
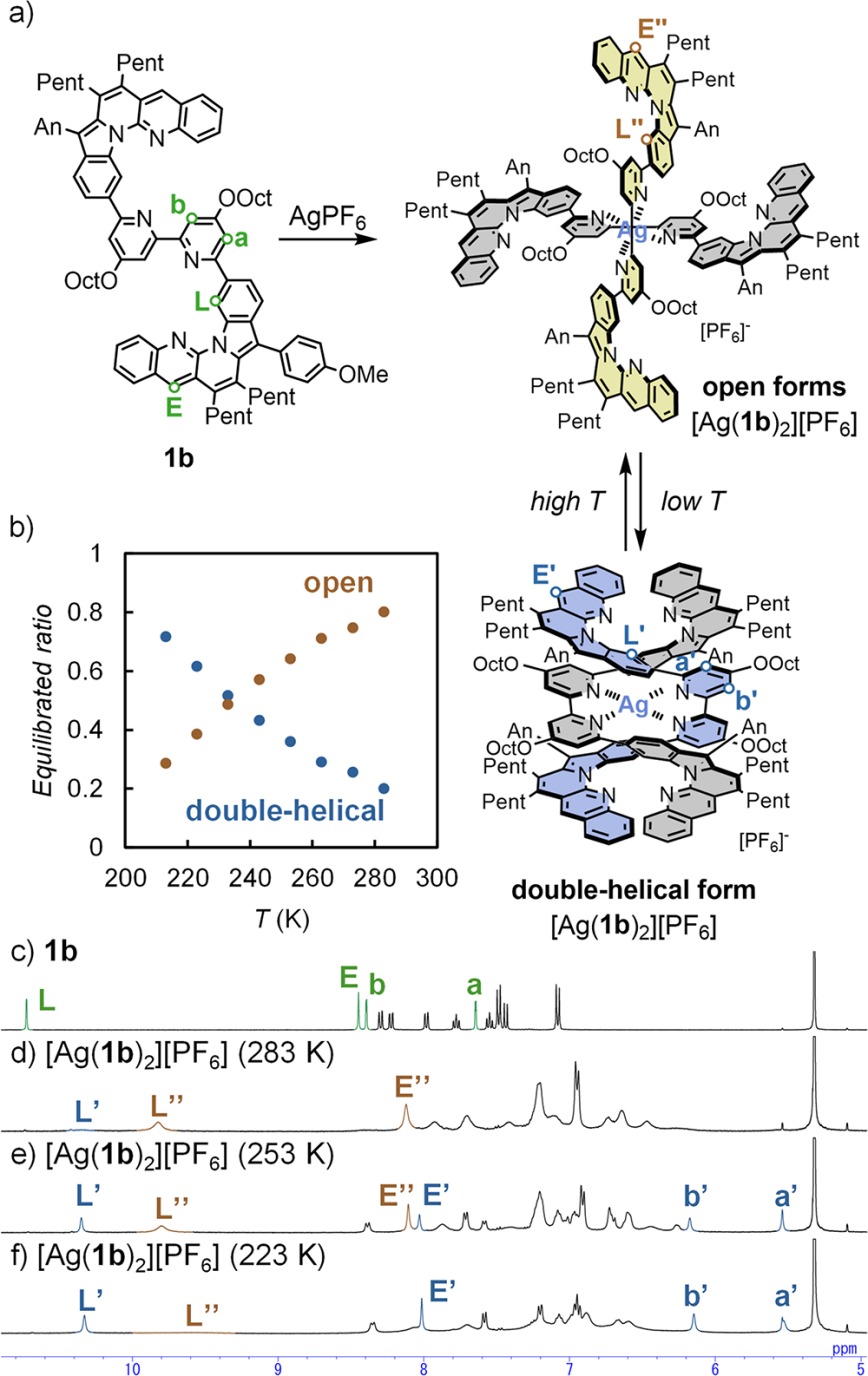
a) Complexation of **1b** with AgPF_6_ and dynamic behavior of the monometallofoldamer [Ag­(**1b**)_2_]­[PF_6_]. b) Relative ratio for open and double-helical forms of [Ag­(**1b**)_2_]­[PF_6_] (CD_2_Cl_2_, [Ag­(**1b**)_2_]­[PF_6_] = 1.0 mM); ^1^H NMR analyses (5–11 ppm) of c) for **1b** in CD_2_Cl_2_ at 298 K and [Ag­(**1b**)_2_]­[PF_6_] in CD_2_Cl_2_ at d) 283 K, e) 253 K, and f) 223 K ([**1b**] = 2.0 mM, [Ag­(**1b**)_2_]­[PF_6_] = 1.0 mM).

Variable-temperature ^1^H NMR revealed the equilibrium between the open and double-helical forms, and their thermodynamic parameters (Δ*G*_open→helix_, Δ*H*_open→helix_, Δ*S*_open→helix_) were determined from the van ’t Hoff plots (Table 1, Figure S12). The double-helical form with dense π-stacking was enthalpically favored at low temperatures but entropically disfavored (Δ*H*_open→helix_ = −3.9 ± 0.2 kcal mol^–1^, Δ*S*_open→helix_ = −16.5 ± 0.7 cal mol^–1^ K^–1^ in CD_2_Cl_2_), which indicates that the open forms possessed high mobility of their L-shaped units and high flexibility in the coordination around Ag­(I). The entropy difference (Δ*S*_open→helix_) for [Ag­(**1b**)_2_]­[PF_6_] was more negative than that of [Zn­(**1b**)_2_]­[OTf]_2_ ^67^ likely due to the increased degrees of freedom of the open forms, which resulted from the longer and more flexible coordination bonds of the Ag­(I) cation. This flexibility allows the complex to adopt either tridentate or bidentate modes in the open forms, thereby increasing the degrees of freedom. The more negative Δ*H*_open→helix_ for [Ag­(**1b**)_2_]­[PF_6_] compared to that in the previously reported [Zn­(**1b**)_2_]­[OTf]_2_ also suggested enthalpic stabilization of the double-helical form of [Ag­(**1b**)_2_]­[PF_6_], which was likely because the flexible coordination bonds of the Ag­(I) cation could easily adopt a distorted tetrahedral geometry in the double-helical form. Similar to the Ag­(I)-based complex, the Cu­(I)-based complex [Cu­(**1b**)_2_]­[PF_6_] also exhibited more negative Δ*S*_open→helix_ and Δ*H*_open→helix_ compared to [Zn­(**1b**)_2_]­[OTf]_2_ (see Supporting Information Section 4).

**Table 1:** Thermodynamic Parameters for Open-to-Double-Helical Form Conversion^a^

Complex	Solvent	Δ*H*_open→helix_ (kcal mol^–1^)	Δ*S*_open→helix_ (cal mol^–1^ K^–1^)	Δ*G*_open→helix_ at 298 K (kcal mol^–1^)	*K*_open→helix_ at 298 K
[Ag(**1b**)_2_][PF_6_]	CD_2_Cl_2_	–3.9 ± 0.2	–16.5 ± 0.7	1.0 ± 0.3	0.18
[Ag(**1b**)_2_][PF_6_]	CDCl_3_	–3.1 ± 0.5	–15.5 ± 2.0	1.5 ± 0.8	0.08
[Cu(**1b**)_2_][PF_6_]	CDCl_3_	–3.8 ± 0.4	–15.8 ± 2.0	0.9 ± 0.5	0.20
[Zn(**1b**)_2_][OTf]_2_^[67]^	CDCl_3_	–2.4 ± 0.3	–7.1 ± 1.0	–0.3 ± 0.4	1.8

a Thermodynamic parameters were determined from the van ’t Hoff plots based on the variable-temperature ^1^H NMR spectra of [Ag­(**1b**)_2_]­[PF_6_], [Cu­(**1b**)_2_]­[PF_6_], and [Zn­(**1b**)_2_]­[OTf]_2_.

### Complexation of [Ag­(**1c**)_2_]­[PF_6_]

[Ag­(**1b**)_2_]­[PF_6_] showed larger Δ*H* and Δ*S* than [Zn­(**1b**)_2_]­[OTf]_2_, as a result of changing the metal cation to one with longer and more labile coordination bonds, which indicated that Ag­(I)-based monometallofoldamers exhibited greater responsiveness to switching^69^ induced by external stimuli such as temperature and solvents. Therefore, it was expected that [Ag­(**1c**)_2_]­[PF_6_] with chiral side chains would exhibit a more significant *M*/*P* helicity switching response induced by solvents compared to that of [Zn­(**1c**)_2_]­[OTf]_2_.

Although ^1^H NMR analysis revealed that [Ag­(**1c**)_2_]­[PF_6_] formed a complex similar to that of [Ag­(**1b**)_2_]­[PF_6_] (Figure S3), the formation of [Ag­(**1c**)_2_]­[PF_6_] under more dilute conditions was required for UV–vis and CD spectroscopy to investigate the *M*/*P* helicity switching of [Ag­(**1c**)_2_]­[PF_6_]. In toluene and CH_2_Cl_2_, UV–vis absorption spectra of the complex [Ag­(**1c**)_2_]­[PF_6_] (5 μM) exhibited a definite hypochromic effect compared with strand **1c** (Figure S19), and the absorption maximum of strand **1c** around 320 nm disappeared upon complexation.^70^ In addition, [Ag­(**1c**)_2_]­[PF_6_] exhibited large Cotton effects compared to marginal Cotton effects of **1c**.^67^ Concentration-dependent measurements revealed that in toluene (5–100 μM), the absorption spectra of [Ag­(**1c**)_2_]­[PF_6_] remained essentially unchanged, whereas the Cotton effects slightly increased at high concentration (Figure S23). In contrast, in CH_2_Cl_2_, a broad absorption band appeared at concentrations below 5 μM in the longer-wavelength region, suggesting the partial dissociation of [Ag­(**1c**)_2_]­[PF_6_] (Figure S24). At 1 μM CH_2_Cl_2_, no Cotton effect was observed. In addition, the free strand **1c** did not exhibit any Cotton effects in the 400–500 nm range, even at high concentrations (Figure S20), indicating that the formation of the double-helical form of [Ag­(**1c**)_2_]­[PF_6_] was important for producing large Cotton effects (Figure S24c). Based on these results, the chiral inversion switching behavior of [Ag­(**1c**)_2_]­[PF_6_] was investigated at 5 μM, in which the formation of the double-helical form was confirmed.

### Chiral Inversion Switching of Monometallofoldamers

Remarkably, [Ag­(**1c**)_2_]­[PF_6_] exhibited a negative Cotton effect in toluene (Δ*ε*_485_ = −72 dm^3^ cm^–1^ mol^–1^) and a positive Cotton effect in CH_2_Cl_2_ (Δ*ε*_485_ = 68 dm^3^ cm^–1^ mol^–1^) for the range of 400–500 nm (Figure 3). The reversal of these Cotton effects with dependence on the solvent was similar to that observed for [Zn­(**1c**)_2_]­[OTf]_2_ (Figure S30; Δ*ε*_485_ = −434 dm^3^ cm^–1^ mol^–1^ in toluene, Δ*ε*_485_ = +211 dm^3^ cm^–1^ mol^–1^ in CH_2_Cl_2_),^67^ which suggested that the double-helical form of [Ag­(**1c**)_2_]­[PF_6_] was biased toward (*M*)-helicity in toluene and (*P*)-helicity in CH_2_Cl_2_. However, the Cotton effect of [Ag­(**1c**)_2_]­[PF_6_] was smaller than that of [Zn­(**1c**)_2_]­[OTf]_2_. This is possibly because the double-helical form is a minority species in the Ag­(I)-based foldamers, with the open form being predominant. While [Zn­(**1c**)_2_]­[OTf]_2_ exhibited large positive Cotton effects in Lewis basic solvents such as acetone and DMSO, [Ag­(**1c**)_2_]­[PF_6_] dissociated in these solvents, which prevented CD measurements. In *i*-PrOH [Ag­(**1c**)_2_]­[PF_6_] exhibited positive Cotton effects.

**Figure 3:**
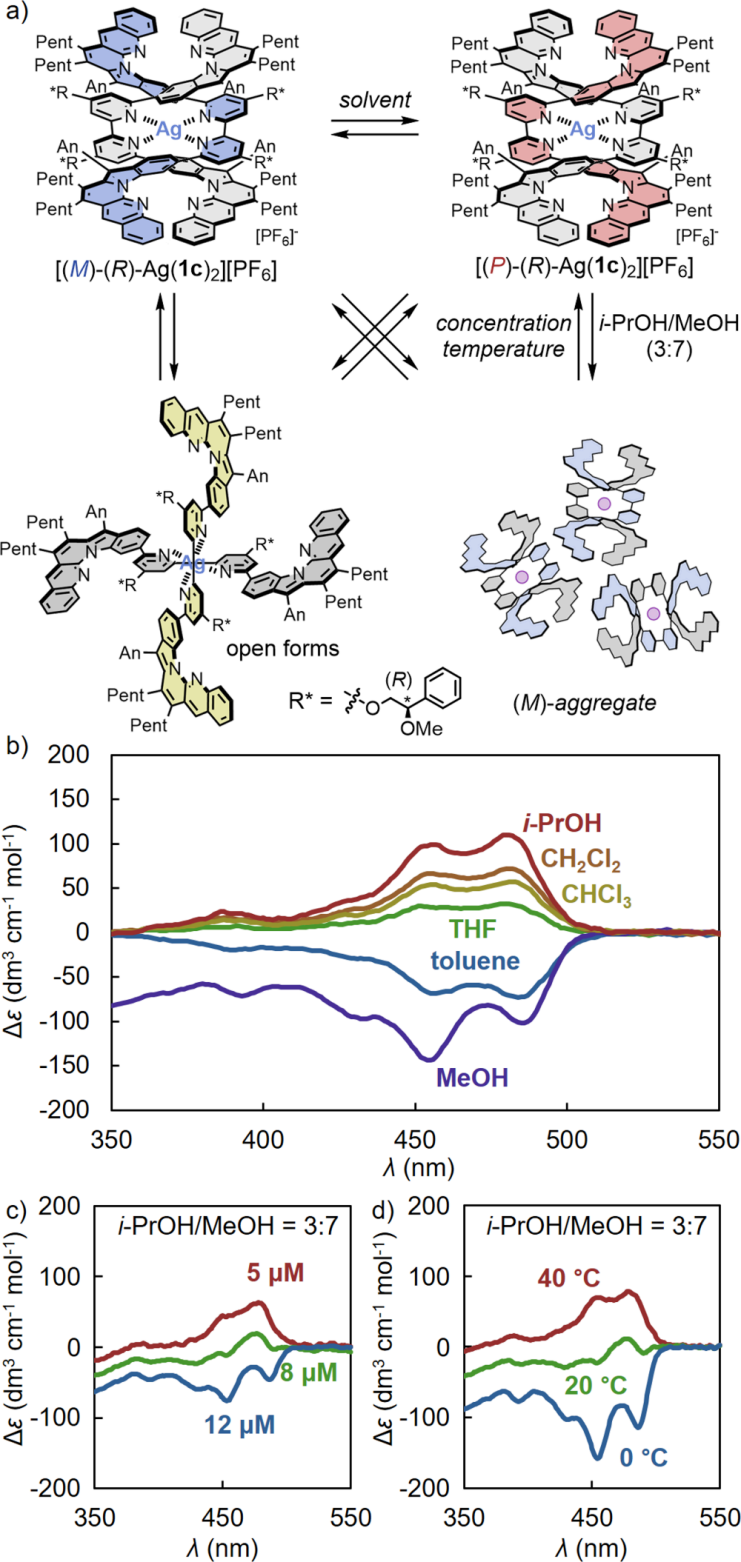
a) Equilibrium between (*M*)-helicity, (*P*)-helicity, and other forms (open forms) and b) CD spectra of [Ag­(**1c**)_2_]­[PF_6_] in *i*-PrOH (red line), CH_2_Cl_2_ (orange line), CHCl_3_ (yellow line), THF (green line), toluene (blue line), and MeOH (purple line) (r.t., [Ag­(**1c**)_2_]­[PF_6_] = 5.0 μM). c, d) CD spectra of [Ag­(**1c**)_2_]­[PF_6_] in *i*-PrOH/MeOH = 3:7. c) Variable-concentration ([Ag­(**1c**)_2_]­[PF_6_] = 12 μM (blue line), 8 μM (green line), and 5 μM (red line) at 30 °C) and d) variable-temperature CD spectra (40 °C (red line), 20 °C (green line), and 0 °C (blue line) at [Ag­(**1c**)_2_]­[PF_6_] = 5.0 μM).

In MeOH, in contrast to a positive Cotton effect of [Zn­(**1c**)_2_]­[OTf]_2_, [Ag­(**1c**)_2_]­[PF_6_] exhibited a larger, broader, and more extended negative Cotton effect around 350–450 nm, along with more pronounced hypochromic absorption than that in toluene (Figures 3b, S32). ^1^H NMR measurements of [Ag­(**1c**)_2_]­[PF_6_] in CD_3_OD showed very broad signals, in contrast to the signal shape observed in CDCl_3_, which suggested the aggregation of [Ag­(**1c**)_2_]­[PF_6_] in CD_3_OD (Figure S4). DLS measurements of [Ag­(**1c**)_2_]­[PF_6_] in MeOH (200 μM) also indicated the formation of aggregates exhibiting hydrodynamic diameters greater than 3 nm (Figure S49). Interestingly, while a large negative Cotton effect (Δε∼10^2^ dm^3^ cm^–1^ mol^–1^) was observed at 5 and 100 μM, a weak positive Cotton effect (Δ*ε*_455_ = 21 dm^3^ cm^–1^ mol^–1^) appeared at 1 μM, indicating that aggregation promoted at high concentrations caused the inversion of the Cotton effects to large negative values.^71^

This aggregation-induced inversion of the Cotton effects indicated the potential for chiral inversion switching modulated by the concentration and temperature. [Ag­(**1c**)_2_]­[PF_6_] also exhibited concentration-dependent CD inversion based on aggregation in *i*-PrOH/MeOH (3:7) at 30 °C, switching from a negative Cotton effect at 12 μM (Δ*ε*_455_ = −74 dm^3^ cm^–1^ mol^–1^) to a positive Cotton effect at 5 μM (Δ*ε*_455_ = 45 dm^3^ cm^–1^ mol^–1^) (Figure 3c). Furthermore, variable-temperature CD measurements of [Ag­(**1c**)_2_]­[PF_6_] in *i*-PrOH/MeOH (5.0 μM) revealed a temperature-dependent inversion of the Cotton effects (Δ*ε*_455_ = 69 dm^3^ cm^–1^ mol^–1^ at 40 °C, Δ*ε*_455_ = −22 dm^3^ cm^–1^ mol^–1^ at 20 °C, and Δ*ε*_455_ = −157 dm^3^ cm^–1^ mol^–1^ at 0 °C). This CD inversion switching was reproducible for at least three cycles (Figures 3d and S29a). The similarity of the broad Cotton effects observed at high concentrations and low temperatures to those in MeOH, combined with the reversal of the Cotton effects upon an increase in the water content in the *i*-PrOH/H_2_O system (Figure S22), suggested that the CD inversion switching in [Ag­(**1c**)_2_]­[PF_6_] was induced by aggregation. Such CD inversion can be attributed to conformational changes in the chiral side chains upon aggregate formation, which leads to an inversion of the helix-sense preference (Figure S39).

[Ag­(**1c**)_2_]­[OTf] with a different counteranion was prepared next from AgOTf to investigate the effect of the counteranion. While [Ag­(**1c**)_2_]­[OTf] exhibited solvent-, temperature-, and concentration-dependent CD inversion switching (Figures S33–S35), [Zn­(**1c**)_2_]­[OTf]_2_ did not exhibit such CD inversion (Figures S36, S37). These results suggest that the valence of the complexes, the number of counteranions, and the equilibrium ratio of the open forms are key factors that influence the formation of aggregation, which leads to the metal-cation-dependent chiroptical switching properties. This result is particularly noteworthy because double-helix inversions in response to achiral stimuli have rarely been reported.^32,34–36^ Therefore, the unique double-helix inversions induced by three different stimuli (temperature, concentration, and solvent) observed in monometallofoldamers [Ag­(**1c**)_2_]­[PF_6_]/[OTf] demonstrate that these complexes are promising candidates for chiroptical inversion switching. The observation of such double-helical inversions is significant because it could offer insights into other systems, such as the inverted double-helices of DNA;^72^ only a limited number of molecules have been reported to exhibit double-helix inversion, and the structural and external factors that induce such inversion are unresolved issues.

### CPL Inversion Switching in Response to Achiral Stimuli

This unique double-helix inversion can be applied to CPL inversion switching. Therefore, we examined the fluorescence and CPL properties of the double-helical monometallofoldamer [Ag­(**1c**)_2_]­[PF_6_] that exhibits *M*/*P* helicity inversion switching.

[Ag­(**1c**)_2_]­[PF_6_] exhibited strong fluorescence in CH_2_Cl_2_ and toluene (φ_F_ = 0.69 and 0.79, respectively), comparable to that of strand **1c** (φ_F_ ∼ 0.8) (Figures 4a, S21, and S22, Table 2). In contrast, the quantum yield decreased to φ_F_ = 0.42 in MeOH, possibly due to dynamic quenching caused by the formation of aggregation in the polar solvent. CPL measurements revealed that [Ag­(**1c**)_2_]­[PF_6_] exhibited positive CPL in CH_2_Cl_2_ (*g*_lum_ = 1.9 × 10^–3^, *B*_CPL_ = 33) and negative CPL in toluene (*g*_lum_ = −2.0 × 10^–3^, *B*_CPL_ = 39) at 515 nm (Figure 4b, Table 2), which suggested solvent-dependent *M*/*P* helicity inversion. The *g*_lum_ and *B*_CPL_ values (*g*_lum_ = −4.2 × 10^–4^, *B*_CPL_ = 4.8 at 515 nm) in MeOH were smaller than the *g*_abs_ values from the CD spectra (*g*_abs_ = −4.2 × 10^–3^ at 485 nm), which indicates that aggregation reduced the CPL efficiency (Table 2).

**Table 2:** Optical Properties of [(*R*)-Ag­(**1c**)_2_]­[PF_6_] in CH_2_Cl_2_, Toluene, and MeOH^a^

Solvent	λ_max_ (CD) (nm)	Δ*ε* (dm^3^ cm^–1^ mol^–1^)^b^	*g*_abs_ ^b^	φ_F_ ^c^	λ_em_ (CPL) (nm)^c^	*g*_lum_ ^c^,^d^	*B*_CPL_ ^e^
CH_2_Cl_2_	455, 480	68	2.1 × 10^–3^	0.69	511	1.9 × 10^–3^	33
toluene	457, 484	–72	–1.8 × 10^–3^	0.79	516	–2.0 × 10^–3^	39
MeOH	455, 485	–102	–4.2 × 10^–3^	0.42	491	–6.5 × 10^–4^	4.8

a [(*R*)-Ag­(**1c**)_2_]­[PF_6_] = 5.0 μM at r.t.

b Δ*ε* and *g*_abs_ (= Δ*ε*/*ε*) were measured at 485 nm.

c λ_ex_ = 440 nm.

d *g*_lum_ was measured at 515 nm.

e *B*_CPL_ = *ε*_440_ × φ_F_ × |*g*_lum_|/2.^73^

**Figure 4:**
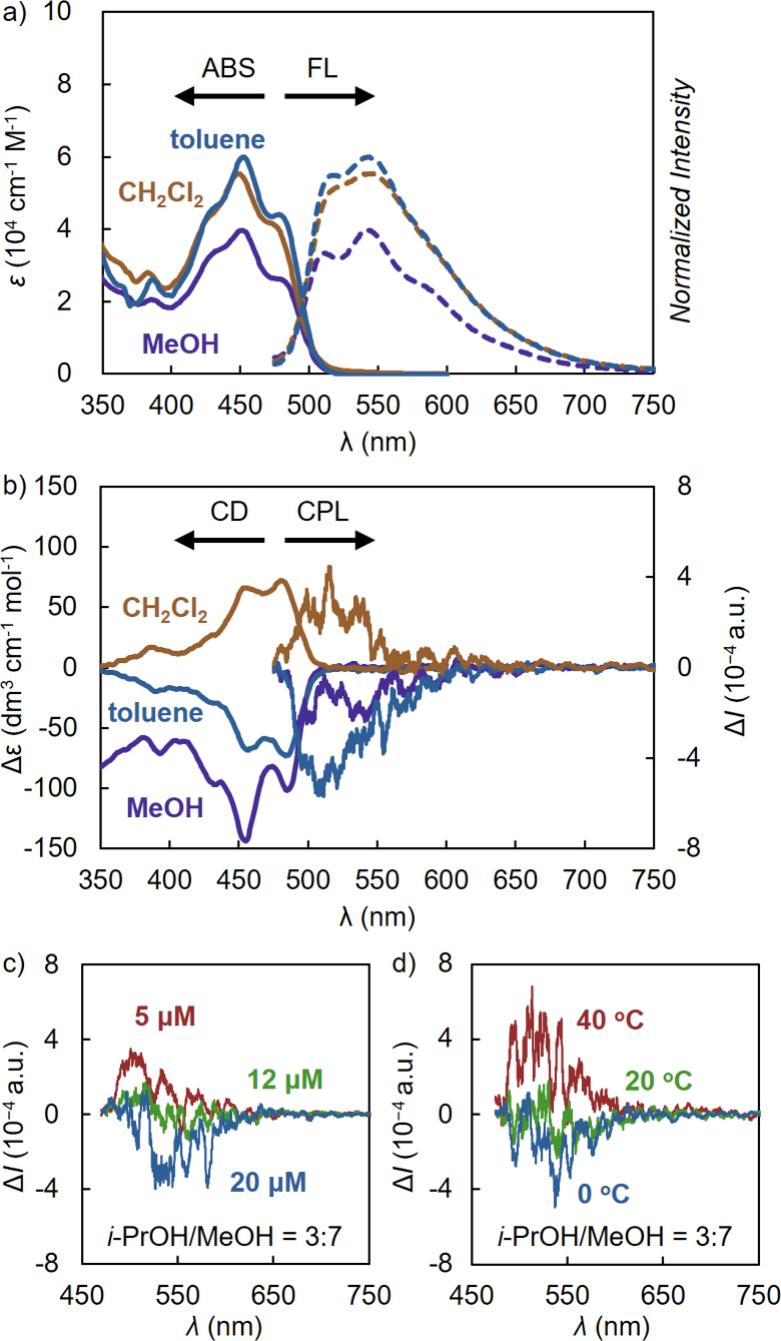
a) UV–vis absorption and fluorescence and b) CD and CPL spectra of [Ag­(**1c**)_2_]­[PF_6_] in CH_2_Cl_2_ (orange line), toluene (blue line), and MeOH (purple line) (r.t., [Ag­(**1c**)_2_]­[PF_6_] = 5.0 μM). c, d) CPL spectra of [Ag­(**1c**)_2_]­[PF_6_] in *i*-PrOH/MeOH = 3:7. c) Variable-concentration ([a] = 20 μM (blue line), 12 μM (green line), and 5 μM (red line) at 30 °C) and d) variable-temperature CPL spectra (40 °C (red line), 20 °C (green line), and 0 °C (blue line) at [Ag­(**1c**)_2_]­[PF_6_] = 7.0 μM).

Notably, the CPL of [Ag­(**1c**)_2_]­[PF_6_] exhibited concentration- and temperature-dependent inversion, consistent with the Cotton effect inversion based on aggregation (Figure 4c,d). In *i*-PrOH/MeOH at 30 °C, [Ag­(**1c**)_2_]­[PF_6_] showed positive CPL at a lower concentration (5.0 μM, *g*_lum_ = 2.6 × 10^–3^ at 515 nm) and negative CPL at higher concentration (20 μM, *g*_lum_ = −1.0 × 10^–3^ at 540 nm) (Figure 4c). The observed red-shift of the CPL maximum suggested the formation of an aggregate. Similarly, in *i*-PrOH/MeOH (7.0 μM), [Ag­(**1c**)_2_]­[PF_6_] also exhibited switchable CPL based on temperature, exhibiting positive CPL at 40 °C (*g*_lum_ = 1.8 × 10^–3^ at 500 nm) and negative CPL at 0 °C (*g*_lum_ = −2.2 × 10^–3^ at 540 nm), accompanied by a redshift (Figure 4d). This thermal CPL inversion was reproducible for at least three cycles, which indicated the long-term stability and reversibility of [Ag­(**1c**)_2_]­[PF_6_] (Figure S48). Although the *g*_lum_ values were moderate (|*g*_lum_| ∼ ±10^–3^), the results demonstrated CPL inversion switching based on double-helix inversion in response to achiral stimuli, which to the best of our knowledge has not been reported for double helices.

## Conclusions

In summary, double-helical monometallofoldamers [Ag­(**1**)_2_]­[PF_6_] were synthesized by complexing bipyridine-type strands **1a**–**c** that featured L-shaped dibenzopyrrolo­[1,2-*a*]­[1,8]­naphthyridine units at both ends with a Ag­(I) cation. In particular, [Ag­(**1c**)_2_]­[PF_6_] not only exhibits strong fluorescence but also undergoes *M*/*P* helicity inversion in response to solvent (Δ*ε* ∼ ±10^2^, *g*_abs_ ∼ ±10^–3^) and exhibits temperature- and concentration-dependent CD inversion switching due to aggregation in *i*-PrOH/MeOH. This behavior is distinct from that of previously reported [Zn­(**1c**)_2_]­[OTf]_2_, where such CD inversion was not observed. The differences in coordination geometry, the valence of the complexes, and the number of counteranions between the Ag­(I) and Zn­(II) cations significantly influence their conformational properties and aggregation propensity. This demonstrates that the chiroptical switching properties of monometallofoldamers can be easily tuned by selection of a metal cation. Furthermore, [Ag­(**1c**)_2_]­[PF_6_] exhibited strong fluorescence and inversion-switchable CPL in response to solvents, concentration, and temperature. The chiral switching observed in the double-helical structure is expected to be applicable to advanced chiral amplification and chiroptical sensing systems. These results provide an important foundation for the design of dynamic metallo-supramolecular systems and the rational selection of metal ions for the development of helical structures as chiroptical switching materials.

## Supplementary Material



## Notes

Experimental procedures; NMR spectra; UV–vis and CD spectra; luminescence and CPL spectra; theoretical studies, DLS profiles; X-ray crystallography data; experimental details of the complexation study (PDF)
