# Supplementary material for: Temperature‑, Concentration‑, and Solvent-Dependent M/P Helicity Switching of Double-Helical Monometallofoldamers with Inversion of Circularly Polarized Luminescence
Source: JACS Au. 2026 Jan 30;6(2):1299–307. doi: 10.1021/jacsau.5c01659 (PMC12933351; doi:10.1021/jacsau.5c01659)
Supplement: Supplementary file 1 [file au5c01659_si_001.pdf]

Supporting Information for

Temperature-, Concentration-, and Solvent-Dependent *M*/*P* Helicity Switching of Double-Helical Monometallofoldamers with Inversion of Circularly Polarized Luminescence

Kotaro Matsumura,[a] Daiki Tauchi,[b] Masashi Hasegawa,[b] Yoshitaka Tsuchido[a] and Hidetoshi Kawai*[a]

[a] Department of Chemistry, Faculty of Science, Tokyo University of Science, 1-3 Kagurazaka, Shinjuku-ku, Tokyo 162-8601, Japan

[b] Department of Chemistry, Graduate School of Science, Kitasato University, 1-15-1 Kitasato, Minami-ku, Sagamihara, Kanagawa 252-0373, Japan

Contents

1. General 2

2. Experimental section 3

3. VT NMR and van ’t Hoff plots for $[Ag(\mathbf{1b})_2][PF_6]$ 10

4. Complexation of strands **1** with Cu(I) cations 16

5. Single crystal X-ray structural analysis 19

6. UV-vis absorption and CD spectra 23

7. Luminescence and CPL spectra 36

8. DLS measurements 40

9. Theoretical calculations 41

10. Cyclic voltammogram of the Cu(I)-based monometallofoldamer 42

11. References 43

1. General

^{1}H NMR spectra were recorded on a Bruker-Biospin·AVANCE NEO 400 (^{1}H: 400 MHz) spectrometer. UV-vis absorption spectra were obtained on a JASCO V-630 spectrophotometer or SHIMADZU UV-3150. Fluorescence spectra were recorded using a JASCO FP-8300 spectrofluorometer equipped with an ILF835 integrating sphere system (100 mm diameter) at room temperature. The fluorescence quantum yields were determined by an absolute method using the integrating sphere setup. CD spectra were taken on JASCO J-820 and CPL spectra were taken on JASCO CPL-300 spectrofluoropolarimeter. The X-ray analysis data was obtained by using a Rigaku XtaLAB Synergy-DW diffractometer. DLS analysis data was obtained by using a Zetasizer Nano ZS. Cyclic voltammetry (CV) was measured in CH_2Cl_2 solutions containing 100 mM nBu$_4$NPF$_6$ using an ALS electrochemical analyzer Model-600A. A platinum electrode served as the working electrode, a platinum wire as the counter electrode, and a Ag/AgCl electrode (0.01 M $AgNO_3$) as the reference electrode. All potentials are reported versus the ferrocene/ferrocenium (Fc/Fc$^+$) couple as an internal standard.

2,2'-[(2,2'-bipyridine)-6,6'-diyl]bis{5-(4-methoxyphenyl)-6,7-dipentyldibenzopyrrolo[1,2-*a*][1,8]naphthyridine} **1a**, 2,2'-[4,4'-dioctoxy(2,2'-bipyridine)-6,6'-diyl]bis{5-(4-methoxyphenyl)-6,7-dipentyldibenzopyrrolo[1,2-*a*][1,8]naphthyridine} **1b** and 2,2'-{4,4'-bis[(*R*)-2-methoxy-2-phenylethoxy]-(2,2'-bipyridine)-6,6'-diyl}bis{5-(4-methoxyphenyl)-6,7-dipentyldibenzopyrrolo[1,2-*a*][1,8]naphthyridine} **1c** were prepared according to the reported procedures.[S1]

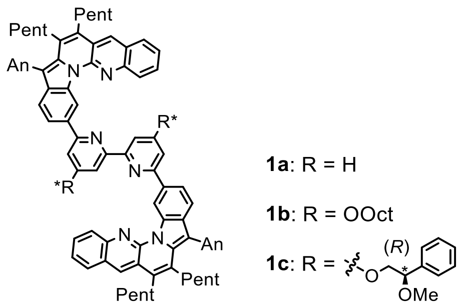

Figure S1. Known compounds.

2. Experimental section

Complexation to [Ag(1b)$_2$][PF$_6$]

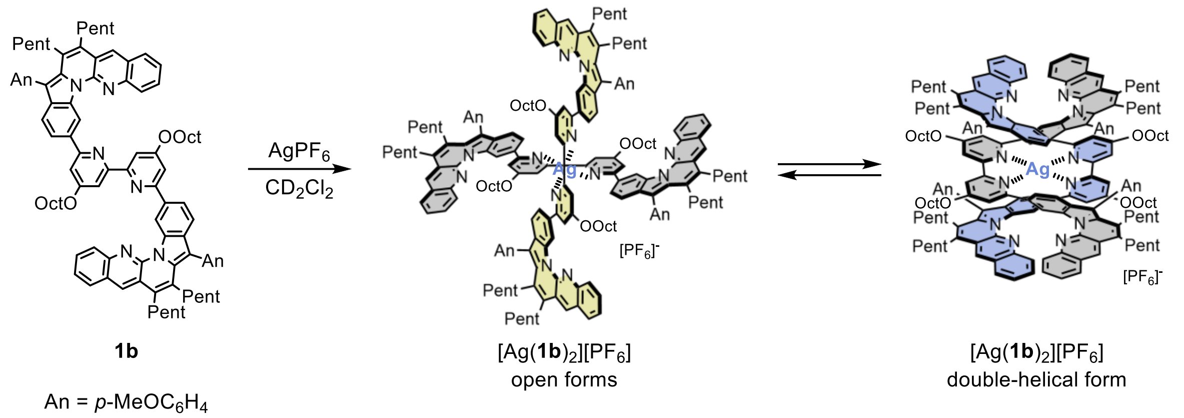

Scheme S1. Complexation of **1b** with AgPF$_6$.

Silver complex [Ag(1b)$_2$][PF$_6$]: To an NMR tube charged with **1b** (1.4 mg, 1.0 μmol) and CD_2Cl_2 (0.50 mL) was added AgPF$_6$ in acetone-*d*$_6$ (50 mM, 10 μL, 0.5 μmol, 0.5.*eq.*). After the mixture was stirred for 1 min, the VT ^{1}H NMR spectra of the resulting mixture were recorded (Figure S2), which suggested the formation of [Ag(**1b**)$_2$][PF$_6$].

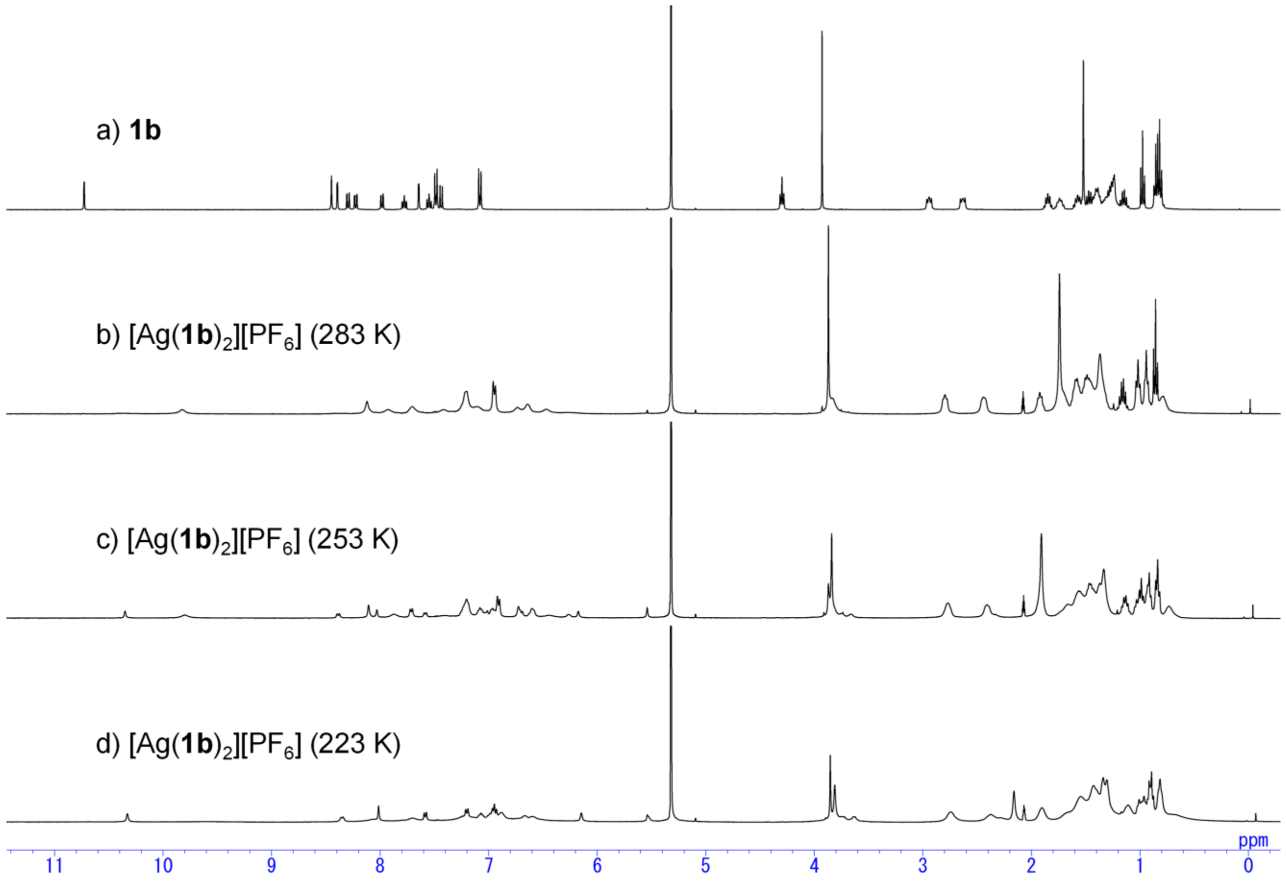

Figure S2. ^{1}H NMR spectra of a) **1b** in CD_2Cl_2 at 298 K, [Ag(**1b**)$_2$][PF$_6$] in CD_2Cl_2 at b) 283 K, c) 253 K, and d) 223 K ([**1b**] = 2.0 mM, [Ag(**1b**)$_2$][PF$_6$] = 1.0 mM).

Complexation to [Ag(1c)$_2$][PF$_6$]

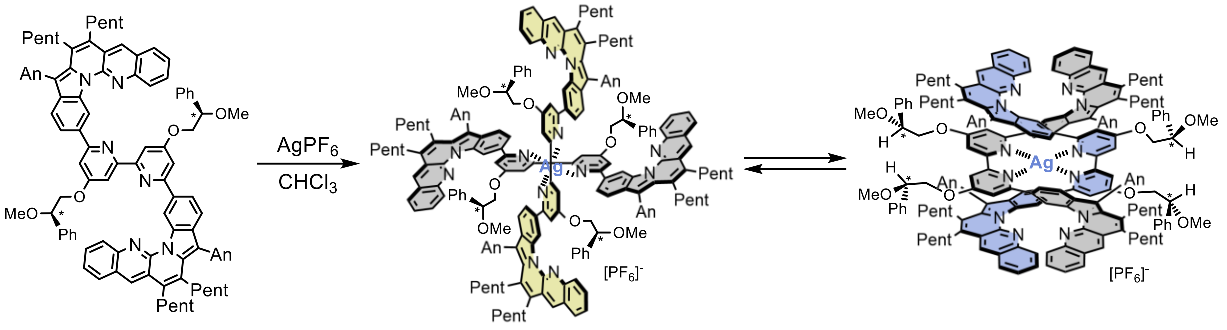

1c

An = *p*-MeOC$_6$H$_4$

[Ag(**1c**)$_2$][PF$_6$]
open forms

[Ag(**1c**)$_2$][PF$_6$]
double-helical form

Scheme S2. Complexation of **1c** with AgPF$_6$.

Silver complex [Ag(1c)$_2$][PF$_6$]: A solution of **1c** (1.5 mg, 1.0 µmol) in CHCl$_3$ (0.50 mL) was added AgPF$_6$ in CD$_3$CN (50 mM, 10 µL, 0.5 µmol, 0.5.*eq.*). After stirring for 1 min, the solution was concentrated in vacuo. The residue was dissolved in CDCl$_3$ (0.50 mL), and ^{1}H NMR spectrum of the resulting mixture was recorded (Figure S3), which suggested the formation of [Ag(**1c**)$_2$][PF$_6$].

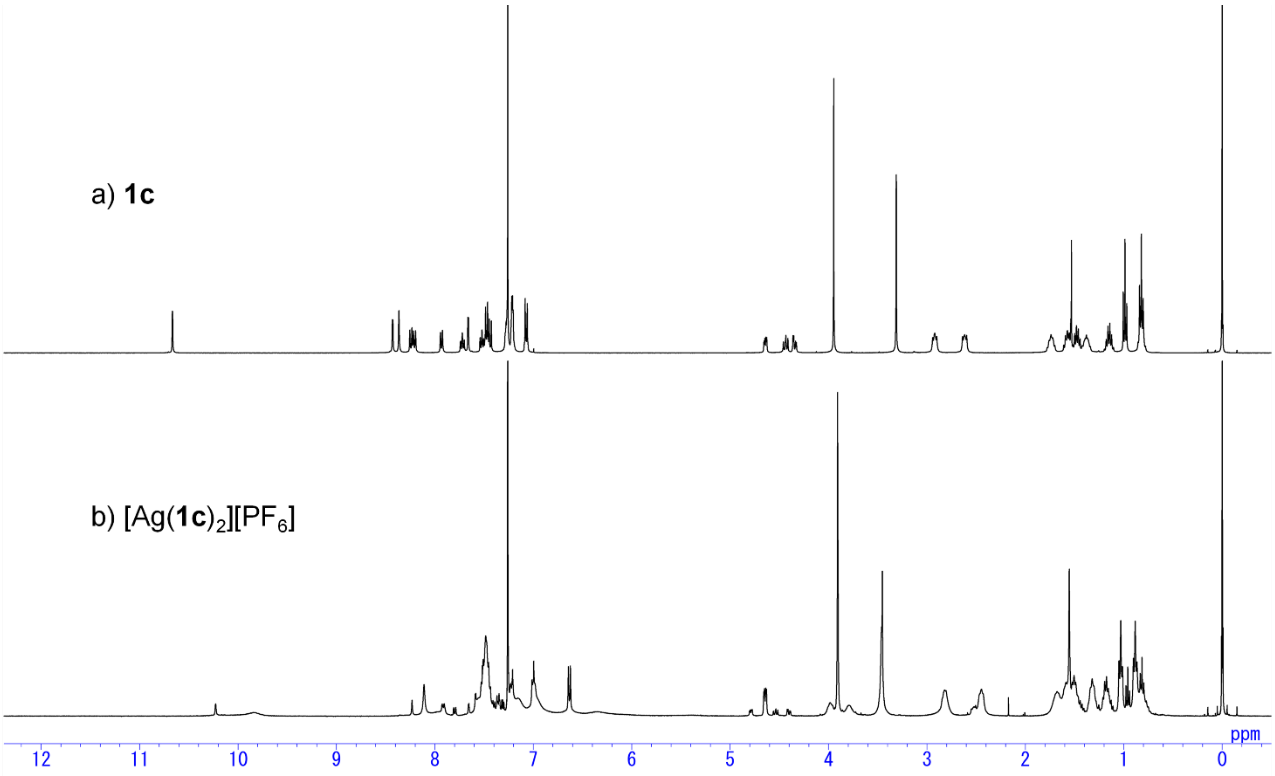

Figure S3. ^{1}H NMR spectra of a) **1c** and b) [Ag(**1c**)$_2$][PF$_6$] in CDCl$_3$ at 298 K ([**1c**] = 2.0 mM, [Ag(**1c**)$_2$][PF$_6$] = 1.0 mM).

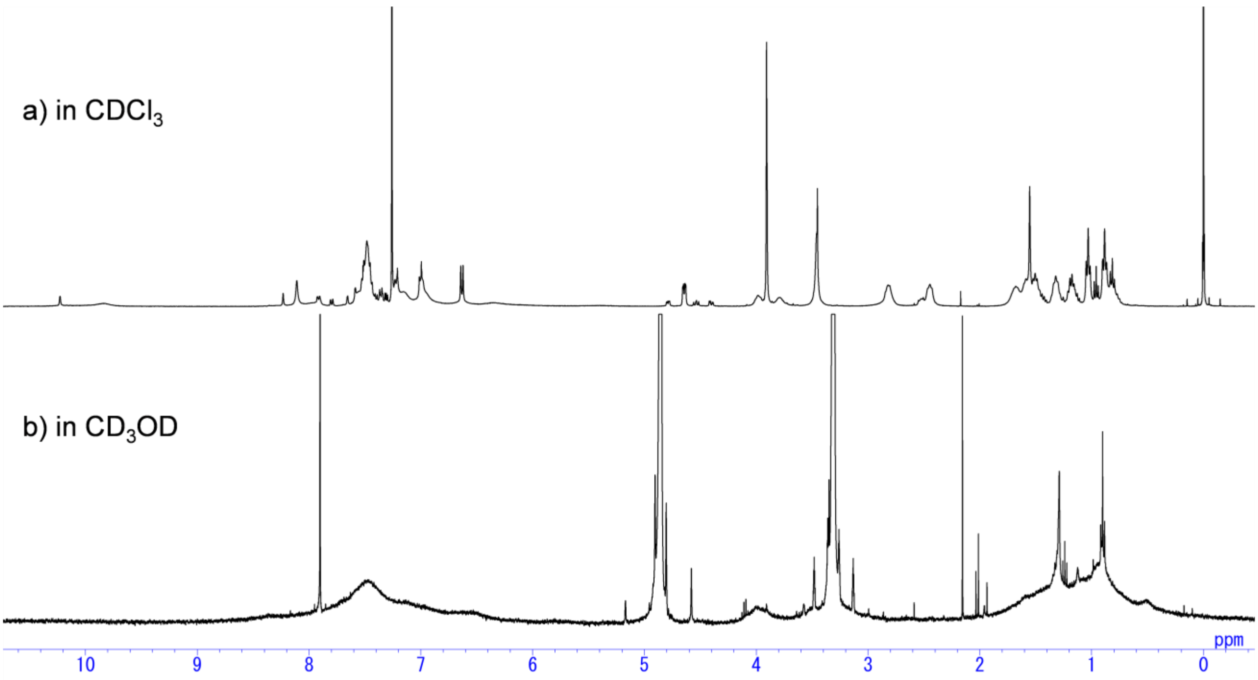

Figure S4. ^{1}H NMR spectra of a) [Ag(**1c**)$_2$][PF$_6$] in $CDCl_3$ and b) in CD_3OD at 298 K ([Ag(**1c**)$_2$][PF$_6$] = 1.0 mM).

Complexation to [Ag(1c)$_2$][OTf]

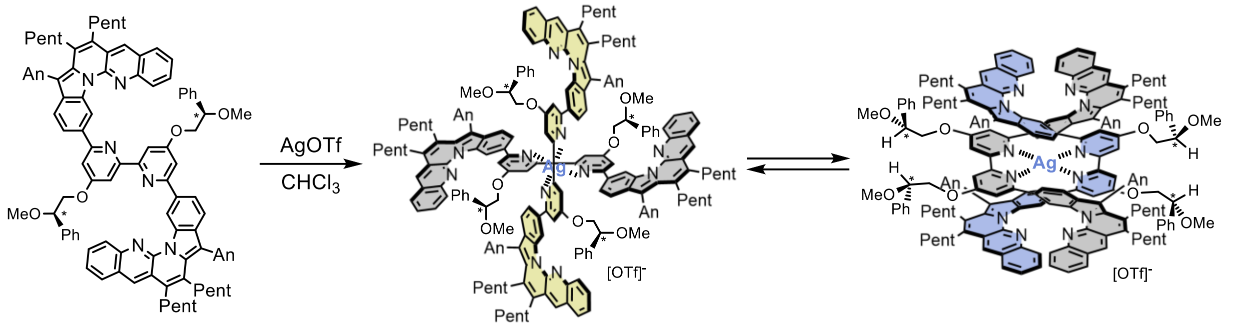

1c

An = *p*-MeOC$_6$H$_4$

[Ag(**1c**)$_2$][OTf]
open forms

[Ag(**1c**)$_2$][OTf]
double-helical form

Scheme S3. Complexation of **1c** with AgOTf.

Silver complex [Ag(1c)$_2$][OTf]: A solution of **1c** (1.5 mg, 1.0 µmol) in CHCl$_3$ (0.50 mL) was added AgOTf in CH$_3$CN (50 mM, 10 µL, 0.5 µmol, 0.5.*eq.*). After stirring for 1 min, the solution was concentrated in vacuo. The residue was dissolved in CDCl$_3$ (0.50 mL), and ^{1}H NMR spectrum of the resulting mixture was recorded (Figure S5), which suggested the formation of [Ag(**1c**)$_2$][OTf].

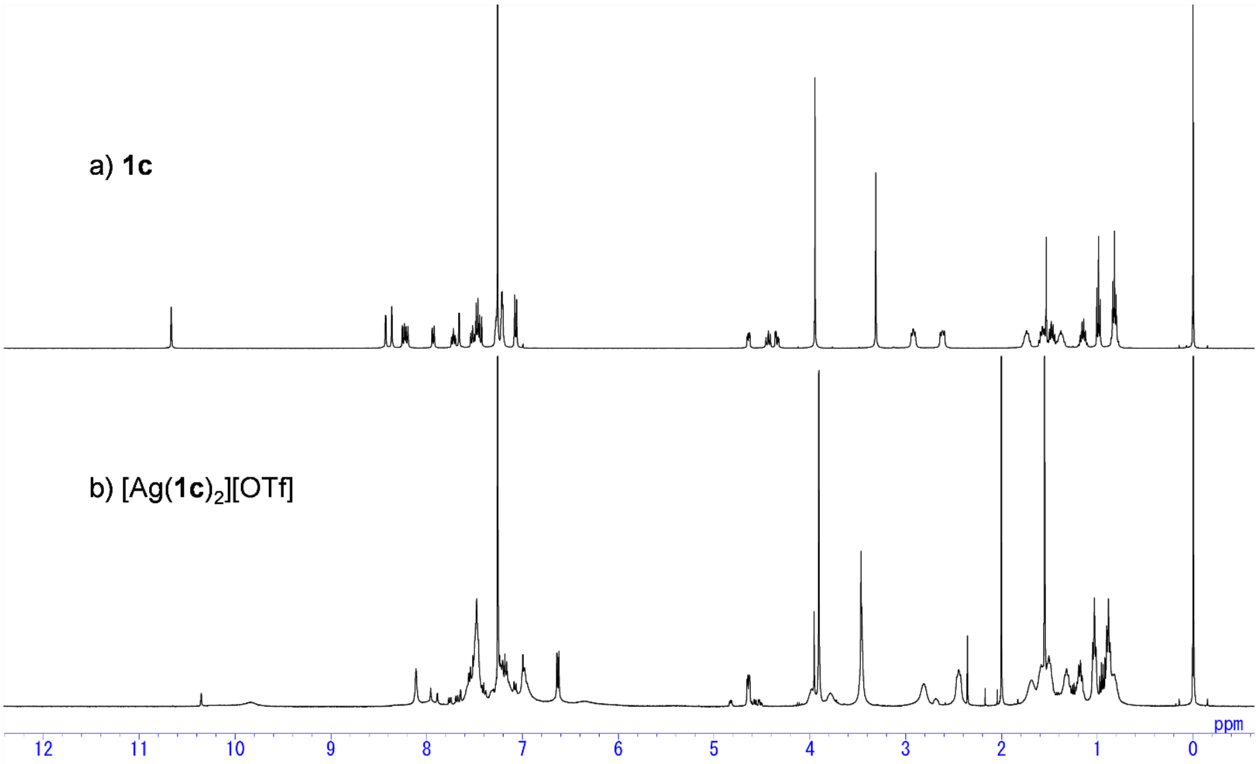

Figure S5. ^{1}H NMR spectra of a) **1c** and b) [Ag(**1c**)$_2$][OTf] in CDCl$_3$ at 298 K ([**1c**] = 2.0 mM, [Ag(**1c**)$_2$][OTf] = 1.0 mM).

Complexation to [Cu(1b)$_2$][PF$_6$]

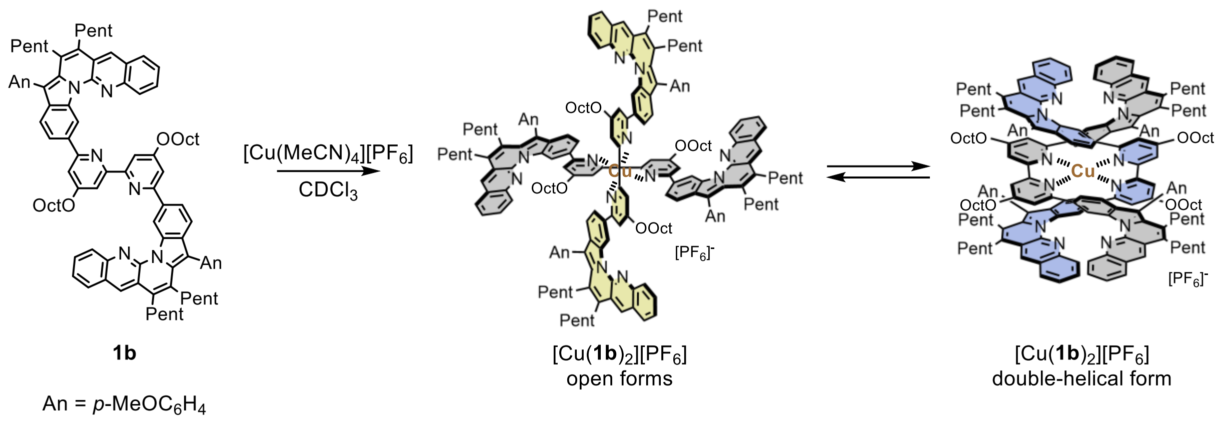

Scheme S4. Complexation of **1b** with [Cu(MeCN)$_4$][PF$_6$].

Cu(I) complex [Cu(1b)$_2$][PF$_6$]: A solution of **1b** (1.4 mg, 1.0 µmol) in CDCl$_3$ (0.50 mL) was added [Cu(MeCN)$_4$][PF$_6$] in acetone-d_6 (50 mM, 10 µL, 0.5 µmol, 0.5.*eq.*). After stirring for 1 min, ^{1}H NMR spectrum of the resulting mixture was recorded (Figure S6), which suggested the formation of [Cu(**1b**)$_2$][PF$_6$].

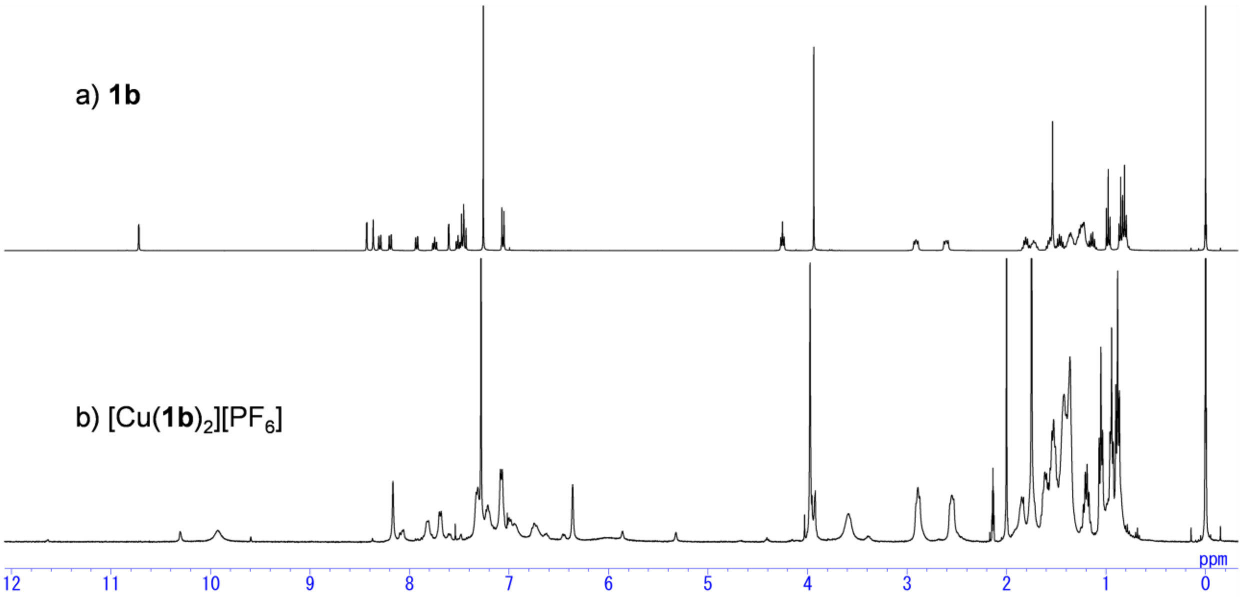

Figure S6. ^{1}H NMR spectra of a) **1b** and b) [Cu(**1b**)$_2$][PF$_6$] in CDCl$_3$ at 298 K ([**1b**] = 2.0 mM, [Cu(**1b**)$_2$][PF$_6$] = 1.0 mM).

Complexation to [Cu(1c)$_2$][PF$_6$]

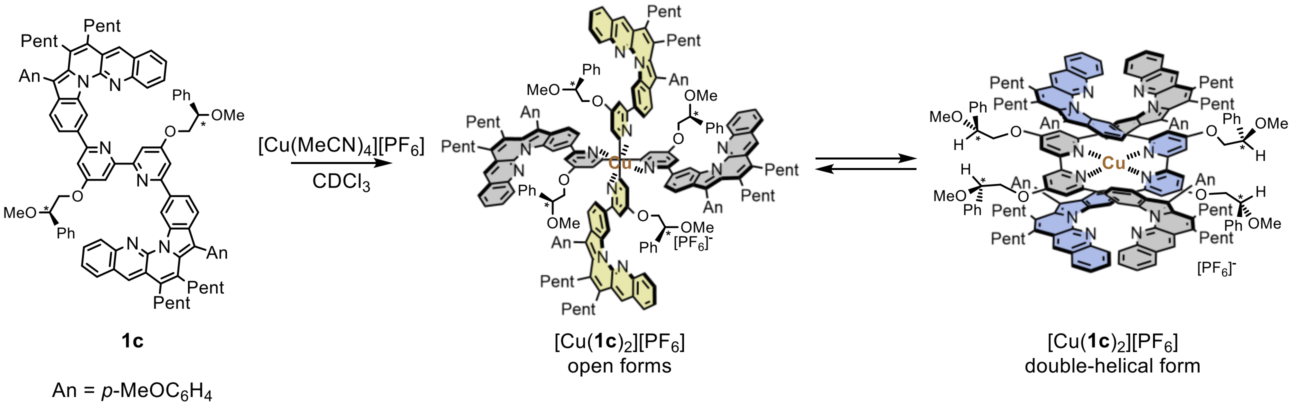

Scheme S5. Complexation of **1c** with [Cu(MeCN)$_4$][PF$_6$].

Cu(I) complex [Cu(1c)$_2$][PF$_6$]: A solution of **1c** (1.5 mg, 1.0 µmol) in CDCl$_3$ (0.50 mL) was added [Cu(MeCN)$_4$][PF$_6$] in acetone-d_6 (50 mM, 10 µL, 0.5 µmol, 0.5*eq.*). After stirring for 1 min, the solution was concentrated in vacuo. The residue was dissolved in CDCl$_3$ (0.50 mL), and ^{1}H NMR spectrum of the resulting mixture was recorded (Figure S7), which suggested the formation of [Cu(**1c**)$_2$][PF$_6$].

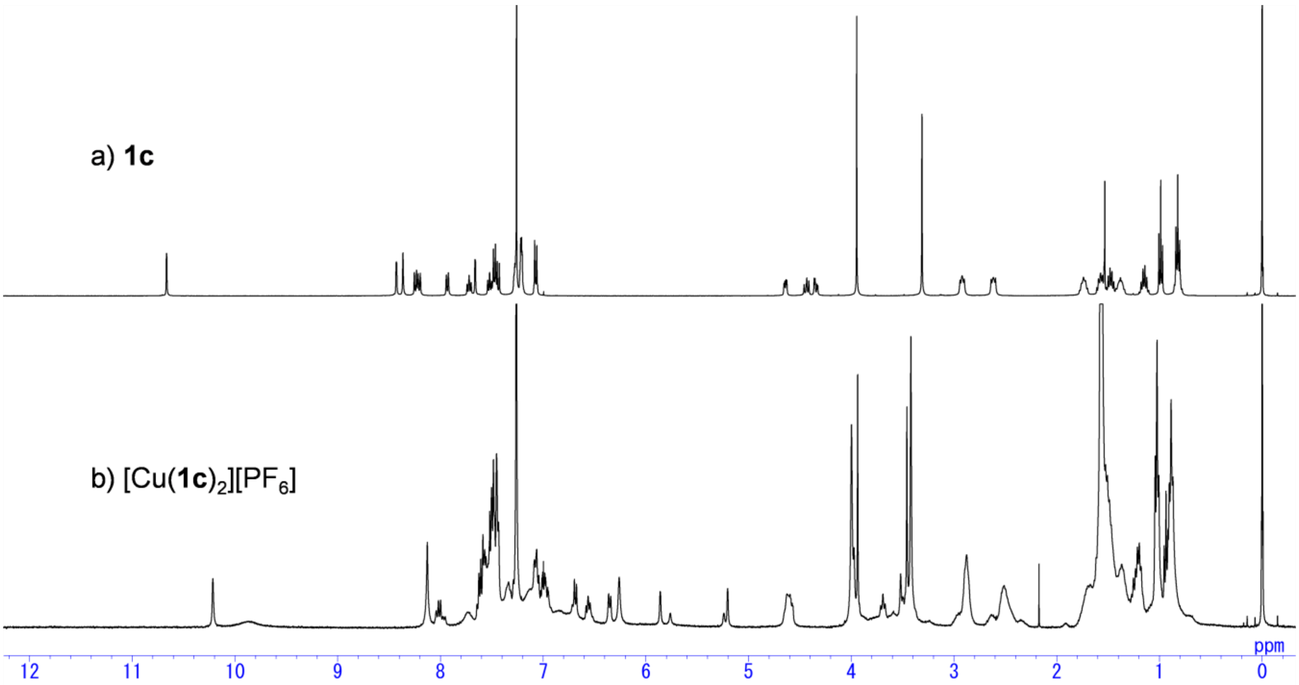

Figure S7. ^{1}H NMR spectra of a) **1c** and b) [Cu(**1c**)$_2$][PF$_6$] in CDCl$_3$ at 298 K ([**1c**] = 2.0 mM, [Cu(**1c**)$_2$][PF$_6$] = 1.0 mM).

Complexation to [Cu(1c)$_2$][OTf]$_2$

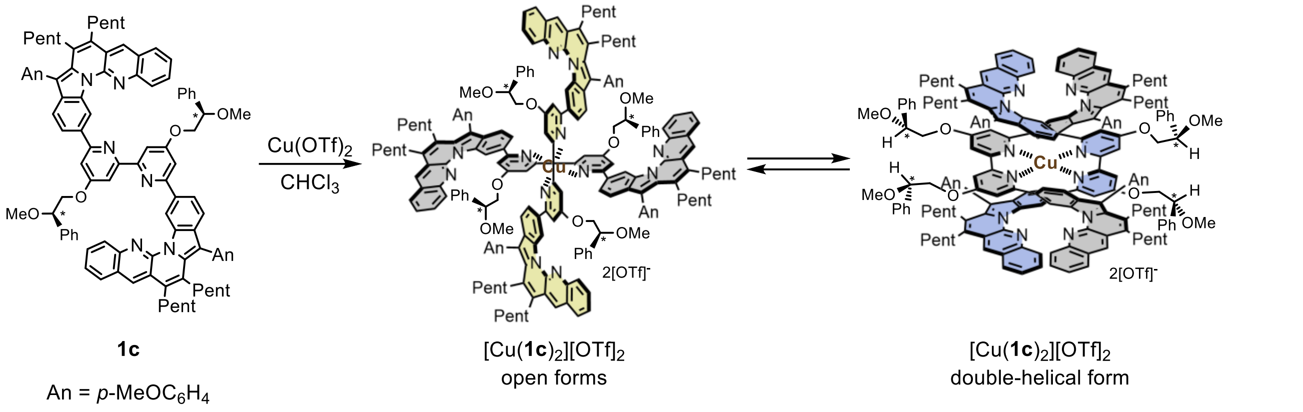

Scheme S6. Complexation of **1c** with Cu(OTf)$_2$.

Cu(II) complex [Cu(1c)$_2$][OTf]$_2$: A solution of **1c** (1.5 mg, 1.0 μmol) in CHCl$_3$ (0.50 mL) was added Cu(OTf)$_2$ in MeCN (50 mM, 10 μL, 0.5 μmol, 0.5.*eq.*) and MS4A (5 mg). After stirring for 1 min, the solution was concentrated in vacuo. The residue was dissolved in CDCl$_3$ (0.50 mL), and ^{1}H NMR spectrum of the resulting mixture was recorded (Figure S8).

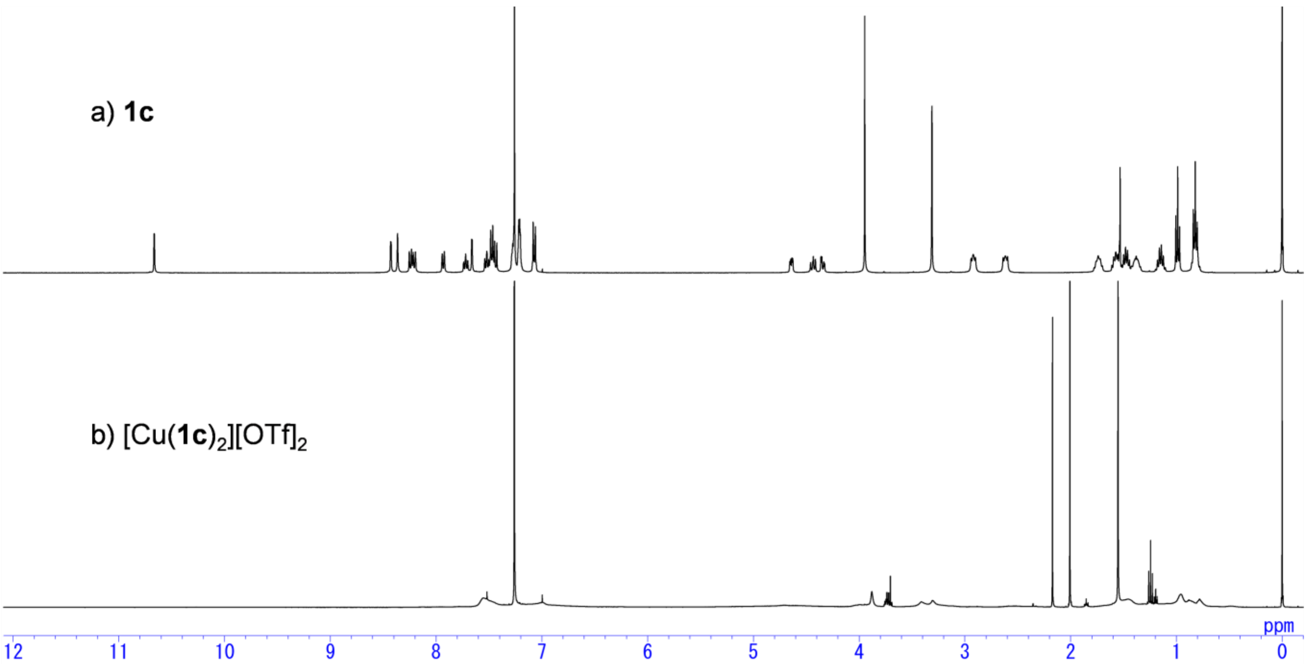

Figure S8. ^{1}H NMR spectra of a) **1c** and b) [Cu(**1c**)$_2$][OTf]$_2$ in CDCl$_3$ at 298 K ([**1c**] = 2.0 mM, [Cu(**1c**)$_2$][OTf]$_2$ = 1.0 mM).

3. VT NMR and van ’t Hoff plots for [Ag(1b)$_2$][PF$_6$]

General method

[Ag(**1b**)$_2$][PF$_6$]: To an NMR tube charged with **1b** (1.4 mg, 1.0 μmol) and $CDCl_3$ (0.50 mL) was added $AgPF_6$ in acetone (50 mM, 10 μL, 0.5 μmol, 0.5*eq.*). After the mixture was stirred for 1 min, the ^{1}H NMR spectrum of the resulting mixture was recorded, which suggested the formation of [Ag(**1b**)$_2$][PF$_6$]. The solution was concentrated in *vacuo*, followed by the addition of solvent (0.50 mL) to the NMR tube. The solution of [Ag(**1b**)$_2$][PF$_6$] (1.0 mM) was subjected to VT NMR analyses.

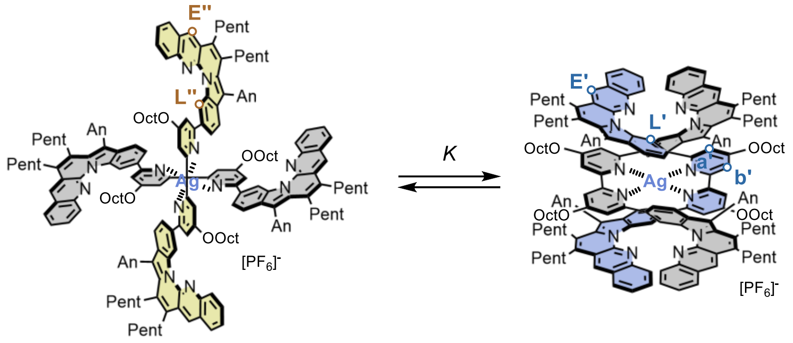

Scheme S7. Dynamic behavior of monometallofoldamer [Ag(**1b**)$_2$][PF$_6$].

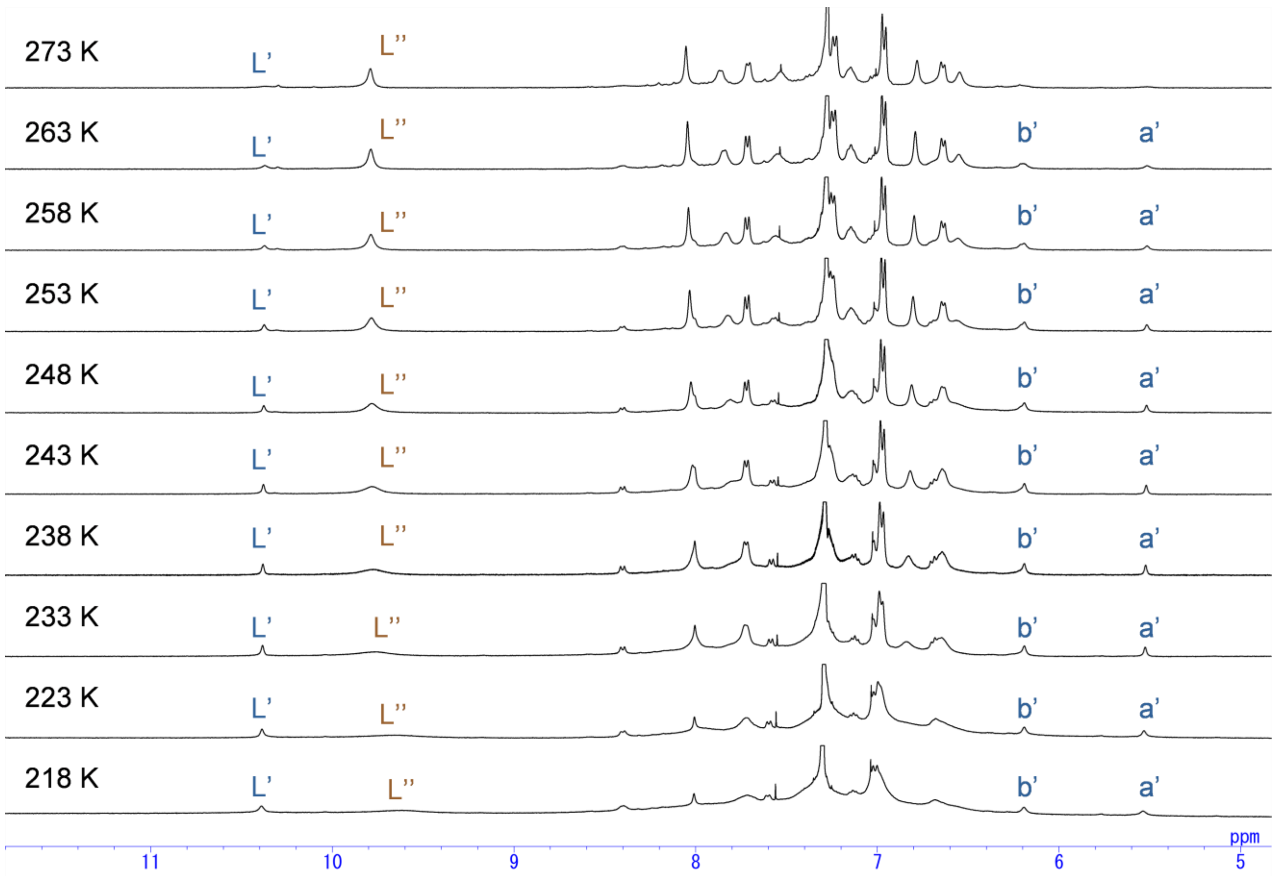

Figure S9. Comparison of representative region of the ^{1}H NMR spectra (400 MHz) of [Ag(**1b**)$_2$][PF$_6$] in $CDCl_3$ (1.0 μM) at various temperatures.

Table S1. Relative ratio of the open form and the double-helical form of [Ag(**1b**)$_2$][PF_6], determined by the integrations of ^{1}H NMR spectra at various temperatures in $CDCl_3$.

	integration		ratio	
temp. (K)	double-helical (L')	open (L")	double-helical	open
263	1.0	6.0	0.14	0.86
258	1.0	5.6	0.15	0.85
253	1.0	4.7	0.17	0.83
248	1.0	4.5	0.18	0.82
243	1.0	3.5	0.22	0.78
238	1.0	3.2	0.24	0.76
233	1.0	2.9	0.26	0.74

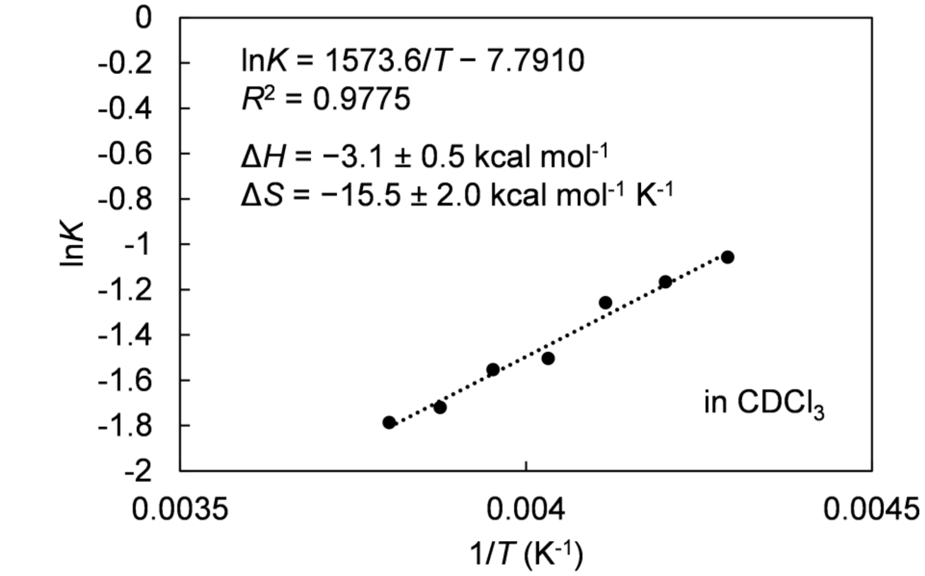

Figure S10. van ’t Hoff plot for the equilibriums of the open forms and the double-helical form of [Ag(**1b**)$_2$][PF_6] in $CDCl_3$.

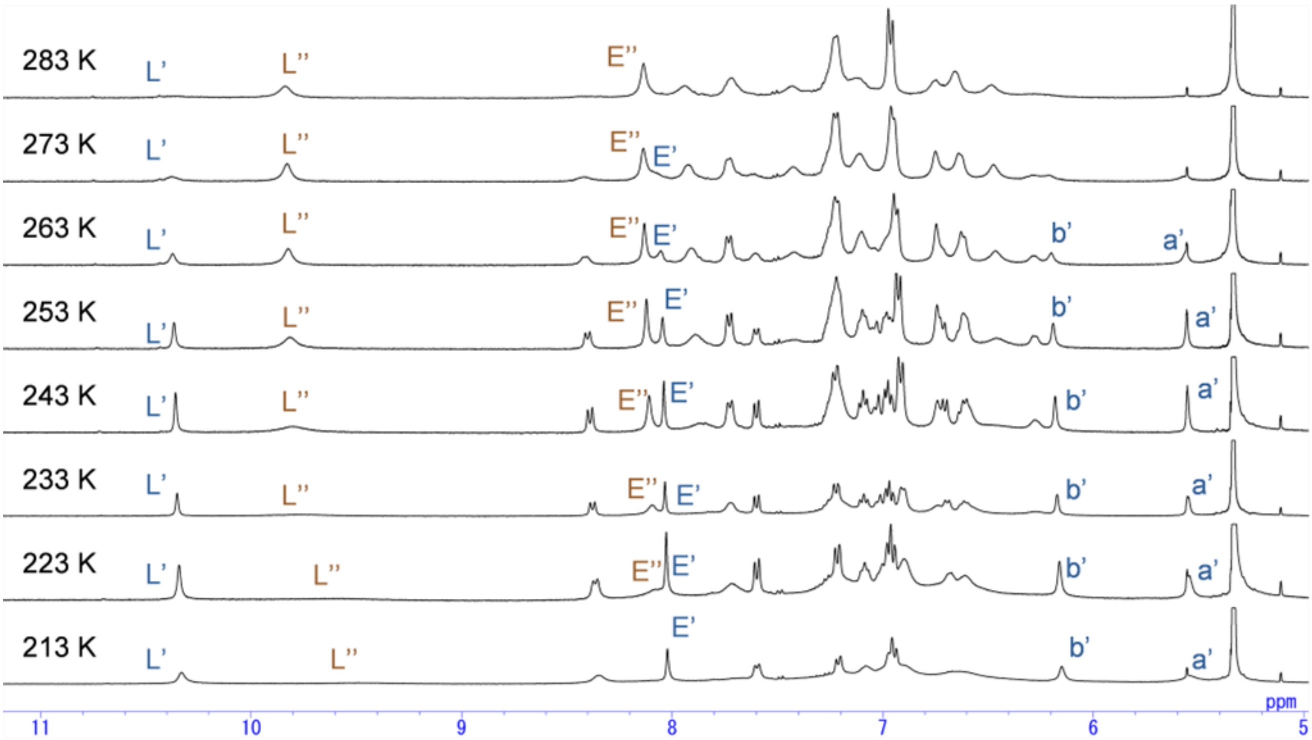

Figure S11. Comparison of representative region of the ^{1}H NMR spectra (400 MHz) of [Ag(**1b**)$_2$][PF$_6$] in CD_2Cl_2 (1.0 μM) at various temperatures.

Table S2. Relative ratio of the open form and the double-helical form of $[Ag(\mathbf{1b})_2][PF_6]$, determined by the integrations of ^1H NMR spectra at various temperatures in CD_2Cl_2.

	integration		ratio	
temp. (K)	double-helical (L')	open (L")	double-helical	open
283	1.0	4.0	0.20	0.80
273	1.0	2.9	0.25	0.75
263	1.0	2.4	0.29	0.71
253	1.0	1.8	0.36	0.64
243	1.0	1.3	0.43	0.57
233	1.0	0.94	0.52	0.48
223	1.0	0.62	0.62	0.38
213	1.0	0.40	0.72	0.28

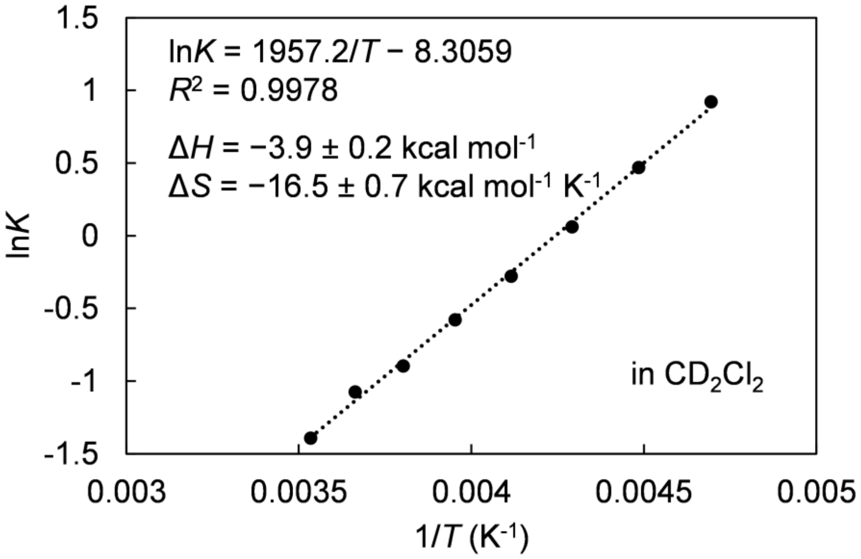

Figure S12. van 't Hoff plot for the equilibriums of the open forms and the double-helical form of $[Ag(\mathbf{1b})_2][PF_6]$ in CD_2Cl_2.

Table S3. Thermodynamic parameters for conversion of the open form to the double-helical form in $[Ag(\mathbf{1b})_2][PF_6]$.

solvent	ΔH (kcal mol^{-1})	ΔS (cal mol^{-1} K^{-1})	ΔG (298 K) (kcal mol^{-1})	K (298 K)
$CDCl_3$	−3.1 ± 0.5	−15.5 ± 2.0	1.5± 0.8	0.08
CD_2Cl_2	−3.9 ± 0.2	−16.5 ± 0.7	1.0 ± 0.3	0.18

Conformational isomer candidates for the open forms

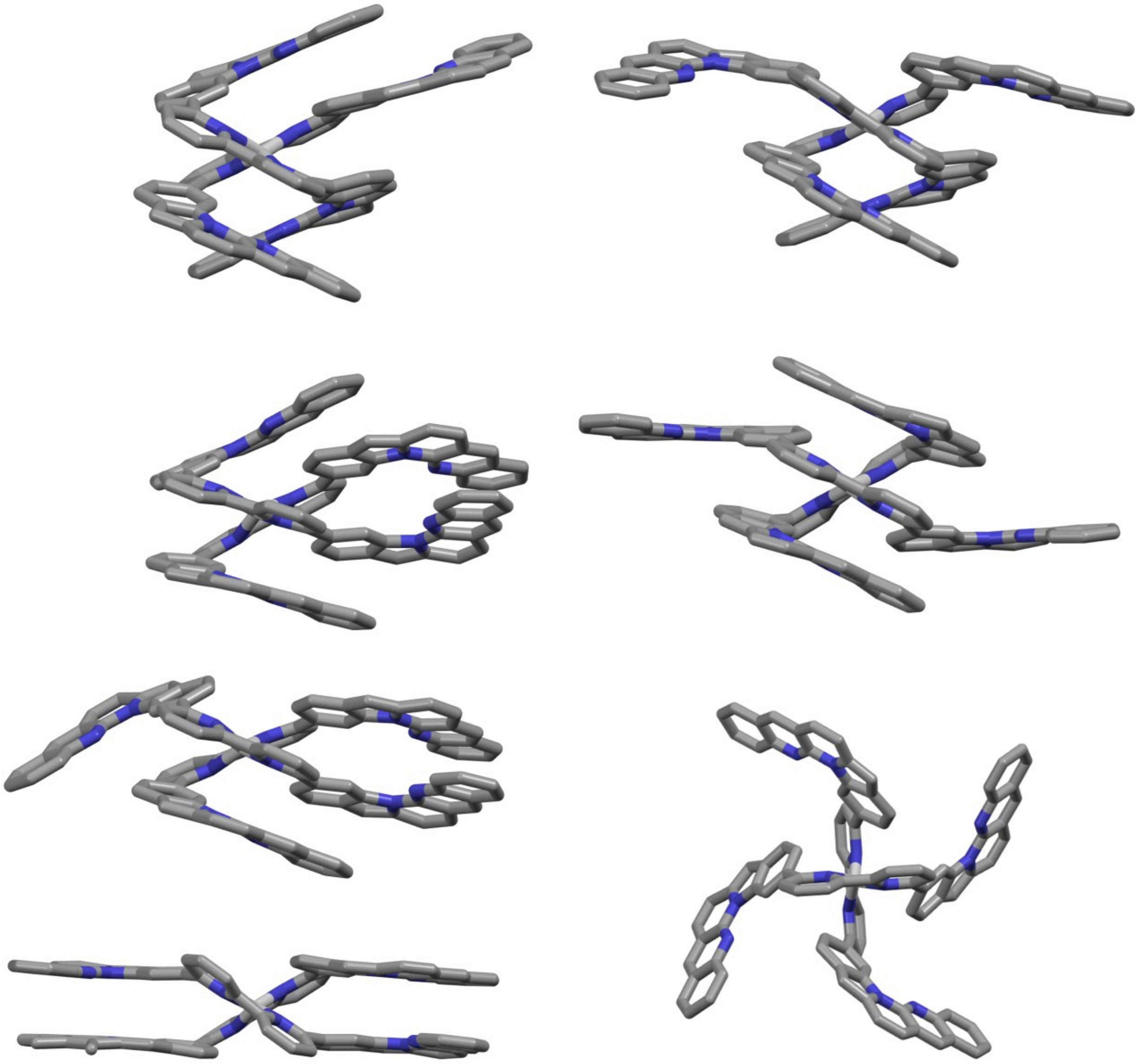

Figure S13. Optimized structures of the expected open forms (GFN2-xTB[S2]). Hydrogen atoms and side chains are omitted for clarity.

4. Complexation of strands 1 with Cu(I) cations

To investigate the metal cation dependence of chiral inversion switching in monometallofoldamers, the monometallofoldamers [Cu(**1**)$_2$][OTf]$_2$ and [Cu(**1**)$_2$][PF$_6$] were constructed through the complexation of strand **1** with Cu(I) and Cu(II) cations, in addition to Zn(II) and Ag(I) cations.

The Cu(I) based foldamers [Cu(**1**)$_2$][PF$_6$] were prepared by adding [Cu(MeCN)$_4$][PF$_6$] to a solution of strands **1**, following the same procedure as for the Zn(II) and Ag(I) based foldamers. The ^{1}H NMR spectra of [Cu(**1**)$_2$][PF$_6$] were similar to those of [Zn(**1**)$_2$][OTf]$_2$ and [Ag(**1**)$_2$][PF$_6$], which suggested that the Cu(I) complexes formed a tetracoordinate complex similar to the Zn(II) and Ag(I) based complexes (Figure S16). Furthermore, variable-temperature ^{1}H NMR and the van 't Hoff plots revealed that the open and double-helical forms of [Cu(**1b**)$_2$][PF$_6$] were in equilibrium (Scheme S8, Figure S14). The double-helical form was enthalpically favored, while the open forms were entropically favored ($\Delta H_{\text{open}\rightarrow\text{helix}}$ = –3.7 ± 0.4 kcal mol^{-1}, $\Delta S_{\text{open}\rightarrow\text{helix}}$ = –15.8 ± 1.2 cal mol^{-1} K^{-1} in CDCl$_3$), as observed for [Zn(**1b**)$_2$][OTf]$_2$ and [Ag(**1b**)$_2$][PF$_6$] (Table S5). The entropy difference ($\Delta S_{\text{open}\rightarrow\text{helix}}$) for [Cu(**1b**)$_2$][PF$_6$] was, similarly to [Ag(**1b**)$_2$][PF$_6$], more negative than that for [Zn(**1b**)$_2$][OTf]$_2$, which indicated that the more flexible coordination bonds of Cu(I) and Ag(I) cations might provide increased degrees of freedom of the open forms.

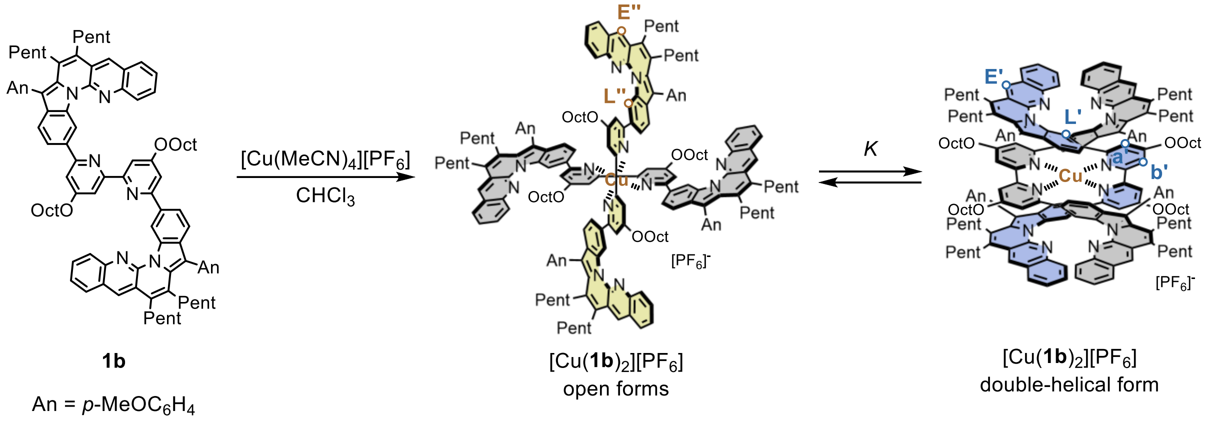

Scheme S8. Complexation of **1b** with Cu(I) and dynamic behavior of monometallofoldamer [Cu(**1b**)$_2$][PF$_6$].

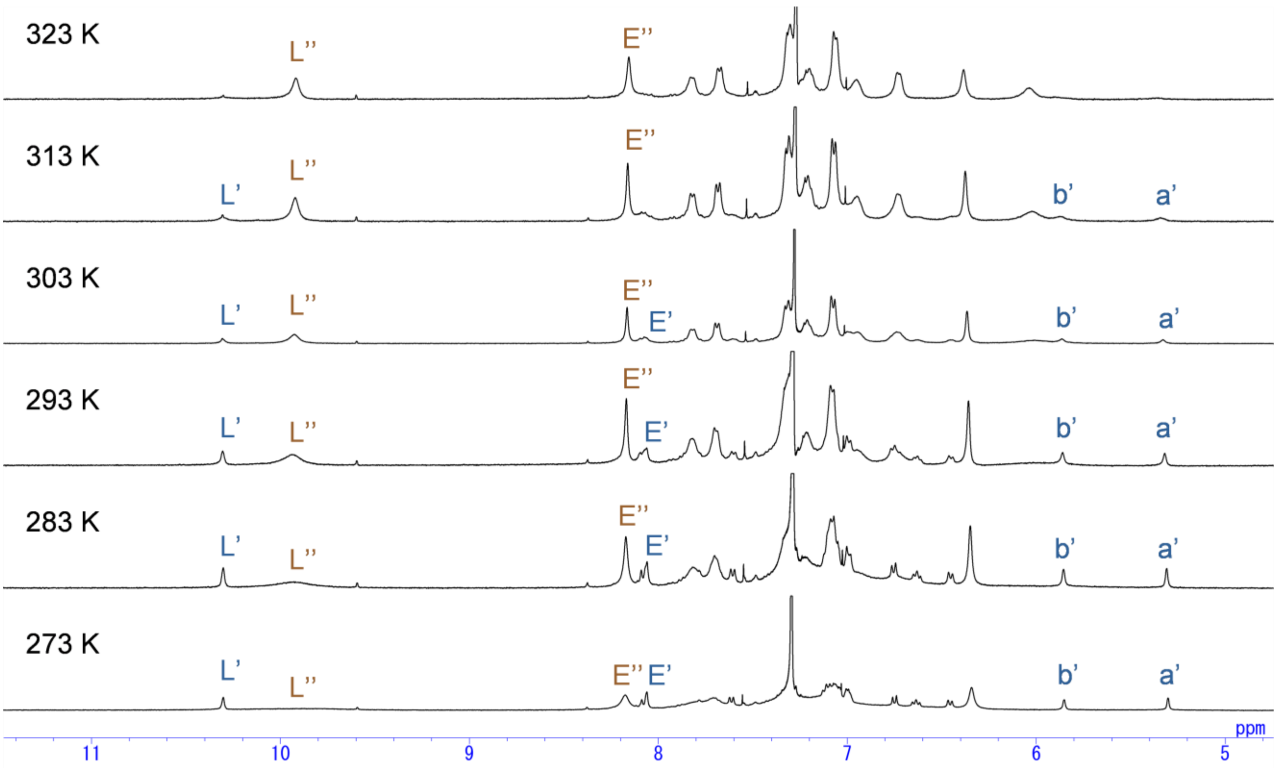

Figure S14. Comparison of representative region of the ^{1}H NMR spectra (400 MHz) of [Cu(**1b**)$_2$][PF$_6$] in $CDCl_3$ (1.0 μM) at various temperatures.

Table S4. Relative ratio of the open form and the double-helical form of [Cu(**1b**)$_2$][PF$_6$], determined by the integrations of ^{1}H NMR spectra at various temperatures in $CDCl_3$.

	integration		ratio	
temp. (K)	double-helical (L')	open (L")	double-helical	open
313	0.15	1.0	0.13	0.87
303	0.18	1.0	0.16	0.84
293	0.23	1.0	0.19	0.81
283	0.28	1.0	0.22	0.78

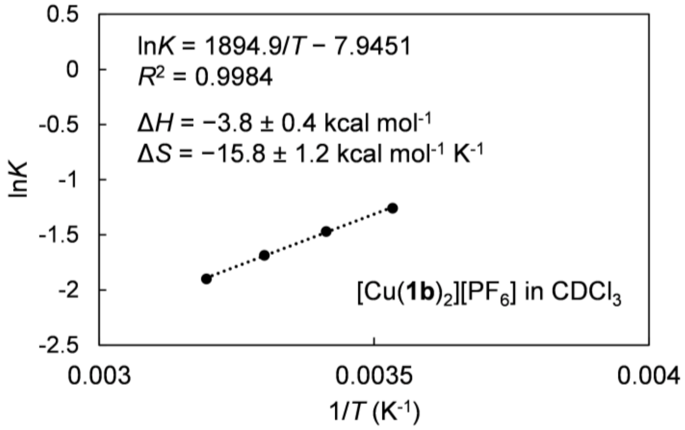

Figure S15. van 't Hoff plot for the equilibriums of the open forms and the double-helical form of [Cu(**1b**)$_2$][PF$_6$] in $CDCl_3$.

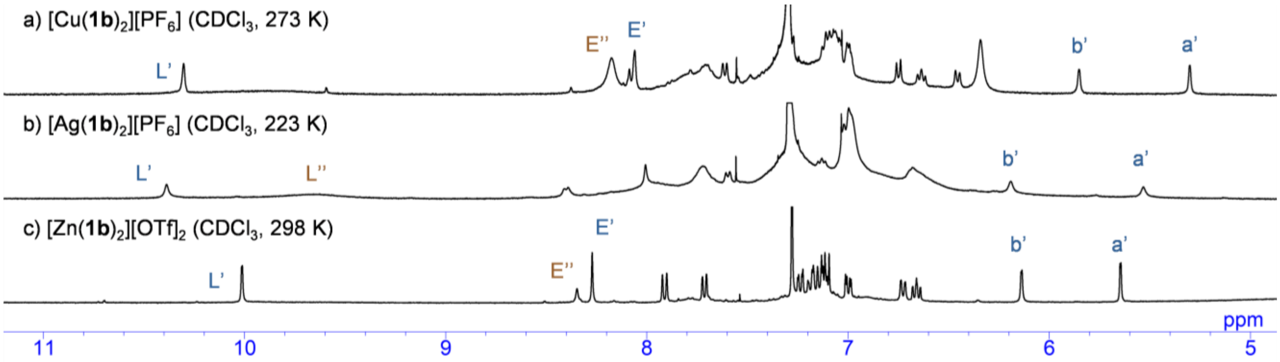

Figure S16. ^{1}H NMR spectra (5–11ppm) of a) [Cu(**1b**)$_2$][PF$_6$] (273 K), [Ag(**1b**)$_2$][PF$_6$] (223 K) and [Zn(**1b**)$_2$][OTf]$_2$ (298 K) in CDCl$_3$.

Table S5. Thermodynamic parameters for open-to-double-helical form conversion

Complex	Solvent	$\Delta H_{open\rightarrow helix}$ (kcal mol^{-1})	$\Delta S_{open\rightarrow helix}$ (cal mol^{-1} K^{-1})	$\Delta G_{open\rightarrow helix}$ at 298 K (kcal mol^{-1})	$K_{open\rightarrow helix}$ at 298 K
[Cu(**1b**)$_2$][PF$_6$]	CDCl$_3$	–3.8 ± 0.4	–15.8 ± 2.0	0.9 ± 0.5	0.20
[Ag(**1b**)$_2$][PF$_6$]	CDCl$_3$	–3.1 ± 0.5	–15.5 ± 2.0	1.5 ± 0.8	0.18
[Zn(**1b**)$_2$][OTf]$_2$[S1]	CDCl$_3$	–2.4 ± 0.3	–7.1 ± 1.0	–0.3 ± 0.4	1.8

5. Single crystal X-ray structural analysis

Although the Cu(II) based foldamers [Cu(**1**)$_2$][OTf]$_2$ were also prepared by adding Cu(OTf)$_2$ to a solution of strands **1**, they could not be characterized by ^{1}H NMR spectroscopy because Cu(II) is paramagnetic. Fortunately, single crystals of [Cu(**1a**)$_2$][OTf]$_2$ were obtained by diffusion method from a C_6H_5Br/hexane mixture, and the double-helical structure of Cu(II) complex was revealed by X-ray crystallography (Figure S17). [Cu(**1a**)$_2$][OTf]$_2$ adopted a double-helical mononuclear structure with two bipyridines coordinated to the Cu(II) cation distorted tetrahedrally (Figure S17f, g). The bipyridine spacer was stacked between the two L-shaped units with a shorter stacking distance of 3.0–3.2 Å compared to 3.2–3.4 Å for [Zn(**1a**)$_2$][OTf]$_2$[S1] (Figure S17e). This shorter distance might be attributed to the shallower angle between the two bipyridine ligands (62.9° for [Cu(**1a**)$_2$][OTf]$_2$ and 70.2° for [Zn(**1a**)$_2$][OTf]$_2$[S1]), which compressed the double helix.

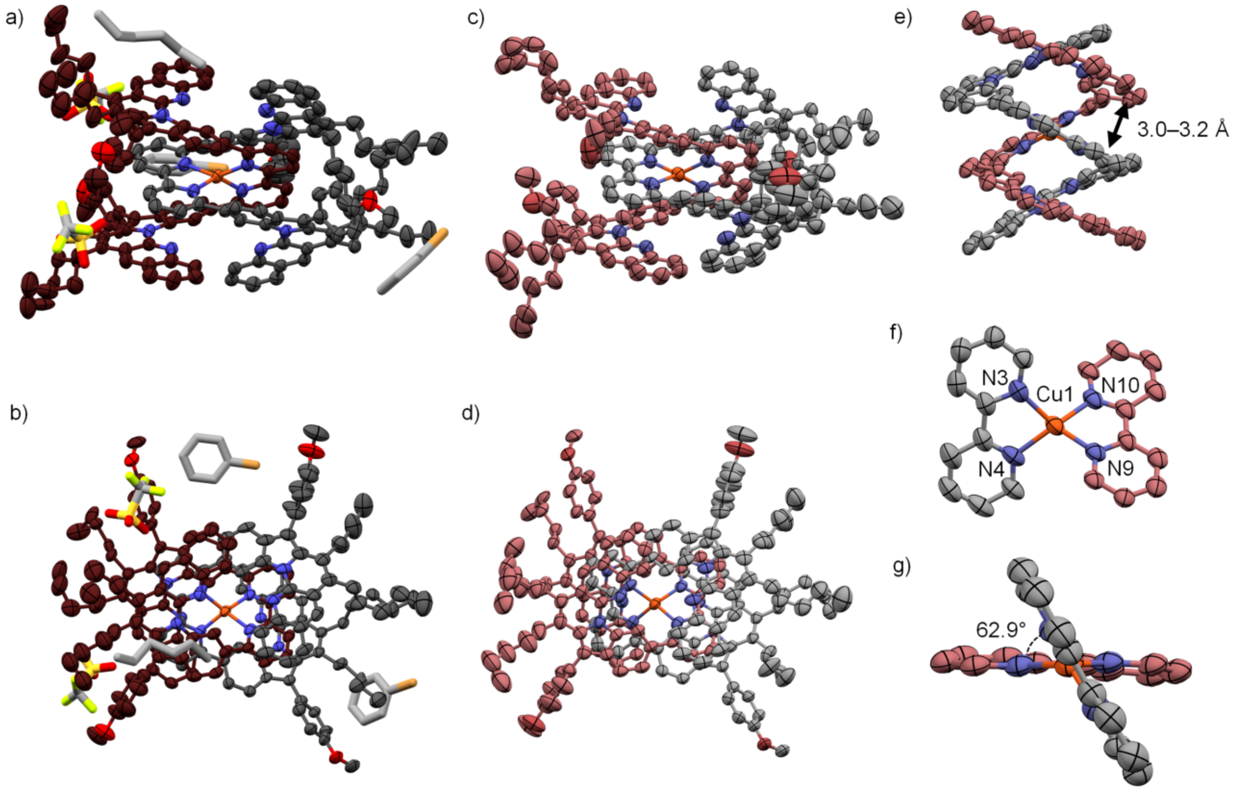

Figure S17. a) Front view and b) Top view of X-ray structures of [Cu(**1a**)$_2$][OTf]$_2$ with the disordered solvents and counter-anions being resolved through the application of isotropic refinement parameters. The solvents and counterions were not adequately refined. The crystal structure of [Cu(**1a**)$_2$][OTf]$_2$ including of solvents and counter-ions, were subjected to analyzed as preliminary examination to verify the placement and quantity of counter-ions (R_1 = 0.24, wR_2 = 0.61). c) Front view, d) Top view and e) Side view of X-ray structures of [Cu(**1a**)$_2$]$^{2+}$ (solvent mask). f, g) Coordination geometry of [Cu(**1a**)$_2$][OTf]$_2$, Selected atom distances and angles: N3-Cu1 2.024(3) Å, N4-Cu1 2.007(3) Å, N9-Cu1 2.024(3) Å, N10-Cu1 1.984(4) Å, N3-Cu1-N4 83.8(1)°, N4-Cu1-N9 113.1(1)°, N9-Cu1-N10 82.9(1)°, N10-Cu1-N3 108.4(1)°.

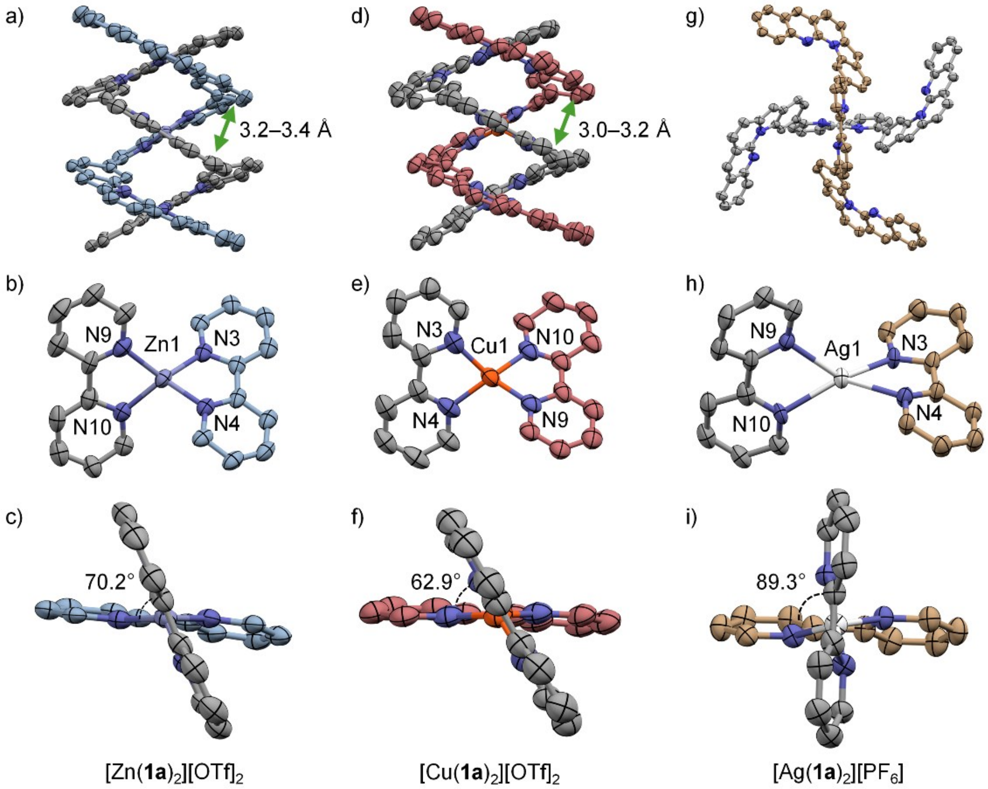

Figure S18. X-ray structures of a–c) [Zn(**1a**)$_2$][OTf]$_2$[S1], d–f) [Ag(**1a**)$_2$][PF$_6$][S1] and g–i) [Cu(**1a**)$_2$][OTf]$_2$ with thermal ellipsoids at 50% probability (pentyl groups, anisyl groups, solvents and counteranions are omitted for clarity).

Single crystal X-ray structural analysis of [Cu(1a)$_2$][OTf]$_2$

General. Suitable crystals for diffraction experiments of compound [Cu(**1a**)$_2$][OTf]$_2$ were obtained by diffusion method from a C_6H_5Br/hexane mixture. The single X-ray structure determination was performed on Rigaku XtaLAB Synergy-DW diffractometer (CuKα radiation, λ =1.54184 Å). A numerical absorption correction (μ) was applied. The structure was solved by direct methods and refined by the full-matrix least-squares method on F^2 with anisotropic temperature factors for nonhydrogen atoms.[S2-4] All the hydrogen atoms were located at the calculated positions and refined with riding. One of the disordered alkyl chains of [Cu(**1a**)$_2$][OTf]$_2$ was restricted by DFIX, DANG, RIGU, ISOR and SIMU. The electron density related to disordered solvent molecules, which could not be represented through discrete atomic positions, was addressed through the implementation of the solvent Mask routine in Olex2. Crystallographic data collection and refinement information is listed in Table S1. The final cif files were checked by IUCR's checkcif.

Alert level A: PLAT029_ALERT_3_A _diffrn_measured_fraction_theta_full value Low . 0.899 Why?

Response: The value of diffrn_measured_fraction_theta_full (0.899) is due to the very weak diffraction at high θ angles. The crystal used for data collection was small (0.05 × 0.2 × 0.3 mm3) and exhibited significant disorder in the alkyl chains, solvent molecules, and counterions, which caused substantial diffuse scattering and further weakened the high-angle reflections. As a result, the completeness in the highest θ shell could not be fully achieved despite attempts to improve the measurement.

Table S6. Crystal data and structure refinement parameters for [Cu(**1a**)$_2$][OTf]$_2$

**Compound**	[Cu(**1a**)$_2$][OTf]$_2$
Data deposition	2512889
Empirical formula	$C_{164}H_{160}N_{12}O_4Cu$ (Solvent Mask)
Formula weight	2426.57
Crystal habit	yellow plate crystal
Crystal size	0.05×0.2×0.3 mm^3
Temperature	93 K
Wavelength	1.54184 Å
Crystal system	monoclinic
Space group	$P2_1/n$
Unit cell dimensions a	37.7065(10) Å
b	22.7565(4) Å
c	22.0201(7) Å
α	90 °
β	125.963(4) °
γ	90 °
Volume	15293.3(9) Å^3
Z	4
Density (calculated)	1.054 g/cm^3
Absorption coefficient Mu	0.618 mm^{-1}
F(000)	5156
Theta range for data collection	2.422 ° to76.516 °
Index ranges	$-45<=h<=46$
	$-28<=k<=24$
	$-27<=l<=26$
Reflections collected	64627
Refinement method	Full-matrix least-squares on F^2
Data/restraints/parameters	27262 /345 /1781
Goodness-of-fit on F^2	1.234
Final R indices	R_1 = 0.1008 [I>2σ(I)] ,
	wR_2 = 0.3254 (all data)

Table S7. Crystal data and structure refinement parameters for [Zn(**1a**)$_2$][OTf]$_2$[S1] and [Ag(**1a**)$_2$][PF_6][S1].

**Compound**		[Zn(**1a**)$_2$][OTf]$_2$[S1]	[Ag(**1a**)$_2$][PF_6][S1]
Data deposition		2354004	2354003
Empirical formula		$C_{164}H_{160}N_{12}O_4Zn$ (Solvent Mask)	$C_{164}H_{160}N_{12}O_4Ag$, PF_6, $C_4H_{10}O$
Formula weight		2428.40	2689.99
Crystal habit		red block crystal	yellow plate crystal
Crystal size		0.1×0.1×0.1 mm^3	0.3×0.2×0.05 mm^3
Temperature		93 K	93 K
Wavelength		1.54184 Å	1.54184 Å
Crystal system		monoclinic	monoclinic
Space group		$P2_1/c$	$P2_1/c$
Unit cell dimensions	a	22.1953(7) Å	29.3564(4) Å
	b	22.8757(7) Å	16.47772(18) Å
	c	30.6110(7) Å	29.4648(3) Å
	α	90 º	90 º
	β	88.224(6) º	90.4168(12) º
	γ	90.533(2) º	90 º
Volume		15541.5(8) $Å^3$	14252.5(3) $Å^3$
Z		4	4
Density (calculated)		1.038 g/cm^3	1.254 g/cm^3
Absorption coefficient Mu		0.629 mm^{-1}	1.793 mm^{-1}
F(000)		5160	5672
Theta range for data collection		2.411 º to 76.457 º	3.000 º to76.687 º
Index ranges		$-27<=h<=27$	$-34<=h<=36$
		$-28<=k<=24$	$-17<=k<=20$
		$-37<=l<=36$	$-36<=l<=37$
Reflections collected		116304	107706
Refinement method		Full-matrix least-squares on F^2	Full-matrix least-squares on F^2
Data/restraints/parameters		31305 /142 /1713	28817 /179 /1818
Goodness-of-fit on F^2		1.051	1.096
Final R indices		R_1 = 0.0866 [I>2σ(I)] , wR_2 = 0.2759 (all data)	R_1 = 0.1205 [I>2σ(I)] , wR_2 = 0.3444 (all data)

6. UV-vis absorption and CD spectra

General procedure on UV-vis and CD measurements for [Ag(1c)$_2$][PF$_6$] and [Ag(1c)$_2$][OTf]

To an NMR tube charged with **1c** (1.5 mg, 1.0 µmol) and $CHCl_3$ (0.50 mL) was added $AgPF_6$ or AgOTf in CH_3CN (50 mM, 10 µL, 0.5 µmol, 0.5*eq.*). After the mixture was stirred for 1 min, the formation of complex [Ag(**1c**)$_2$][PF$_6$] or [Ag(**1c**)$_2$][OTf] was confirmed by ^{1}H NMR spectroscopy (See section 2). The resulting solution was concentrated and dried in *vacuo* to obtain [Ag(**1c**)$_2$][PF$_6$] or [Ag(**1c**)$_2$][OTf] as a yellow solid. The solid of [Ag(**1c**)$_2$][PF$_6$] or [Ag(**1c**)$_2$][OTf] was dissolved in various solvents for UV-vis absorption and CD spectra measurements. The solid-state samples were prepared by mixing the powdered samples with KBr and pressing the mixture into pellets for measurements.

UV-vis absorption spectra of 1c and [Ag(1c)$_2$][PF$_6$] (toluene, CH_2Cl_2, KBr)

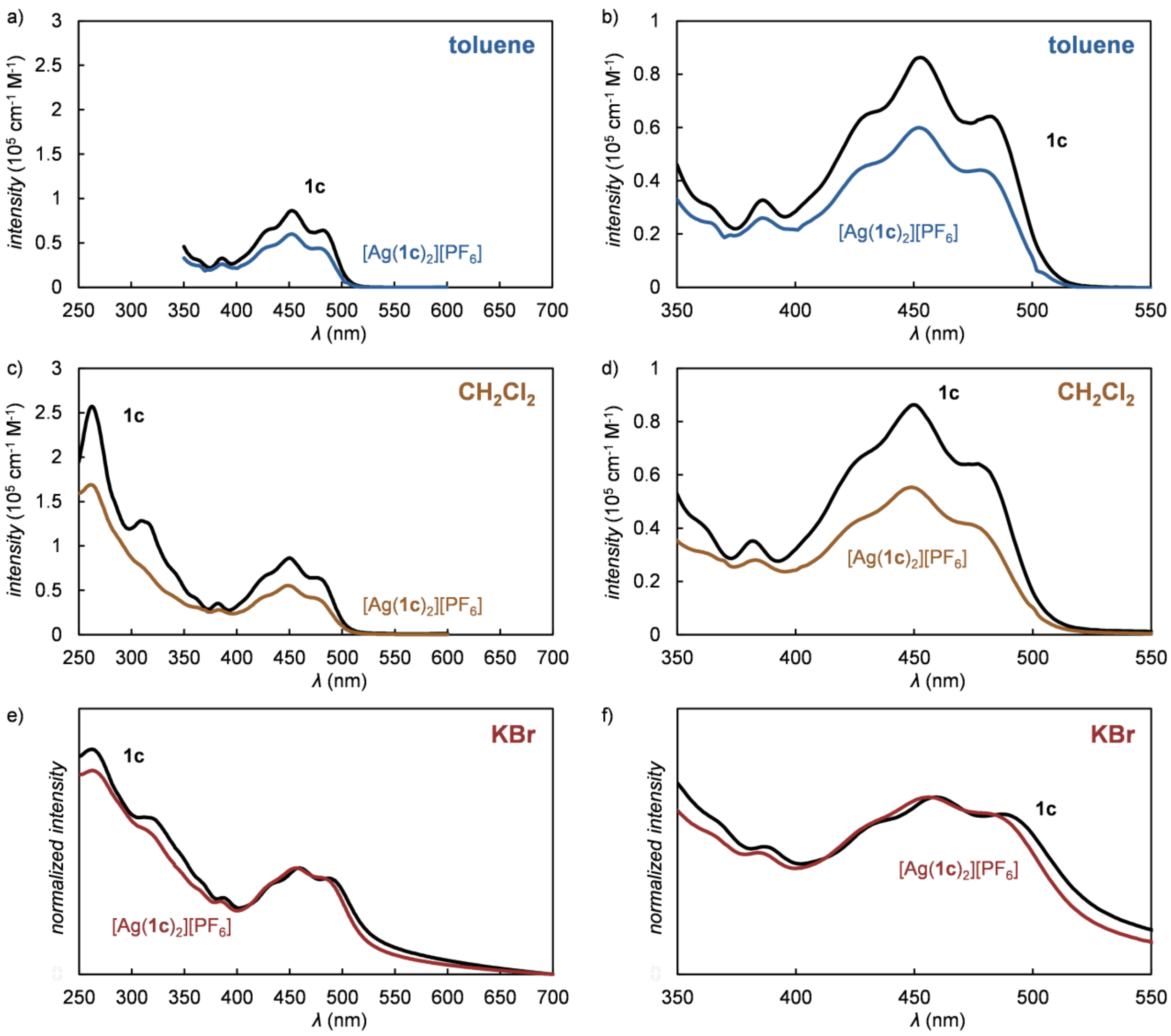

Figure S19. UV-vis absorption spectra of **1c** and [Ag(**1c**)$_2$][PF$_6$] in a, b) toluene, c, d) CH_2Cl_2 and e, f) KBr. UV–vis absorption spectra of **1c** and [Ag(**1c**)$_2$][PF$_6$] in a, b) toluene, c, d) CH_2Cl_2 (r.t., [**1c**] = 10 µM, [Ag(**1c**)$_2$][PF$_6$] = 5.0 µM), and e, f) KBr. For a direct comparison on a per-unit-of-**1c** basis, the absorbance of **1c** is plotted as 2ε, while that of the silver complex [Ag(**1c**)$_2$][PF$_6$] is shown as ε in Figures a–d.

UV–vis absorption and CD spectra of strand **1c** were measured in polar solvent mixtures to investigate the solvophobic effects on strand **1c** (Figure S20). The CD spectra remained silent in the CH_2Cl_2/MeOH (3:7, 50 μM) system, which suggested that strand **1c** does not undergo folding into a chiroptically active conformation under these conditions. Due to the poor solubility of strand **1c**, we were unable to further increase either the concentration or the MeOH content.

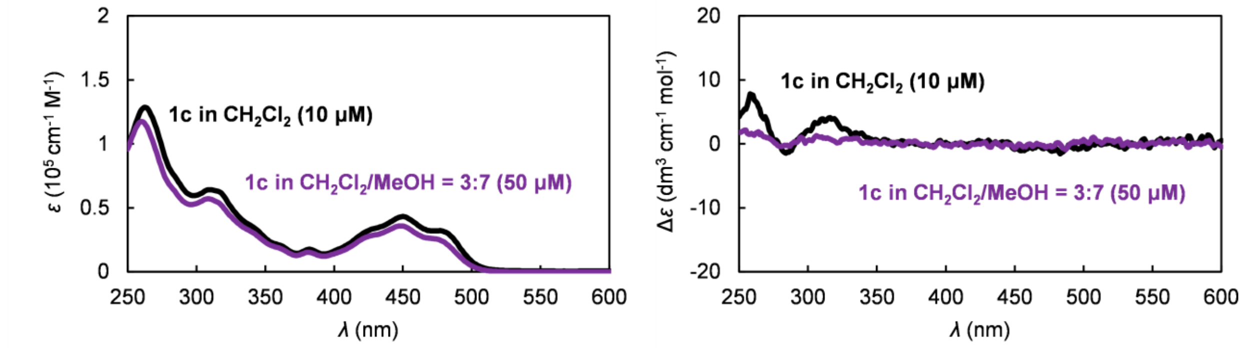

Figure S20. left) UV-vis absorption and right) CD spectra of **1c** in CH_2Cl_2 (10 μM, black line) and CH_2Cl_2/MeOH = 3:7 (50 μM, purple line).

UV-vis absorption and CD spectra of [Ag(1c)$_2$][PF$_6$]

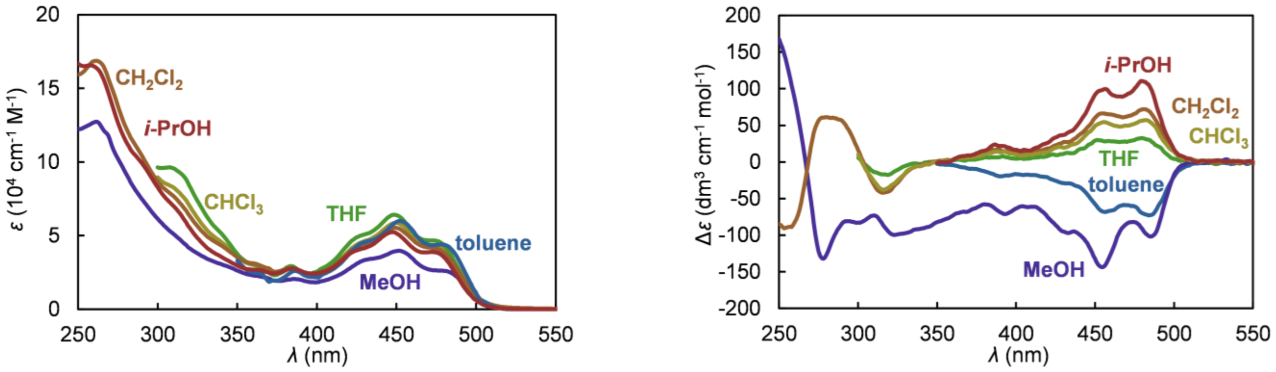

Figure S21. left) UV-vis absorption and right) CD spectra of [Ag(**1c**)$_2$][PF$_6$] in *i*-PrOH (red line), CH_2Cl_2 (orange line), $CHCl_3$ (yellow line), THF (green line), toluene (blue line) and MeOH (purple line) (r.t., [Ag(**1c**)$_2$][PF$_6$] = 5.0 μM).

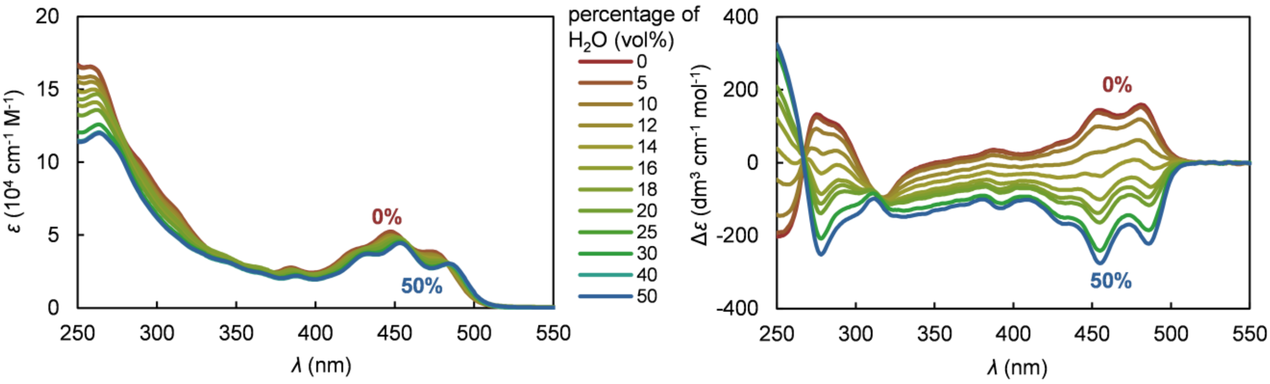

Figure S22. left) UV-vis absorption and right) CD spectra of [Ag(**1c**)$_2$][PF$_6$] (5.0 μM) at various *i*-PrOH/H_2O ratios (vol%).

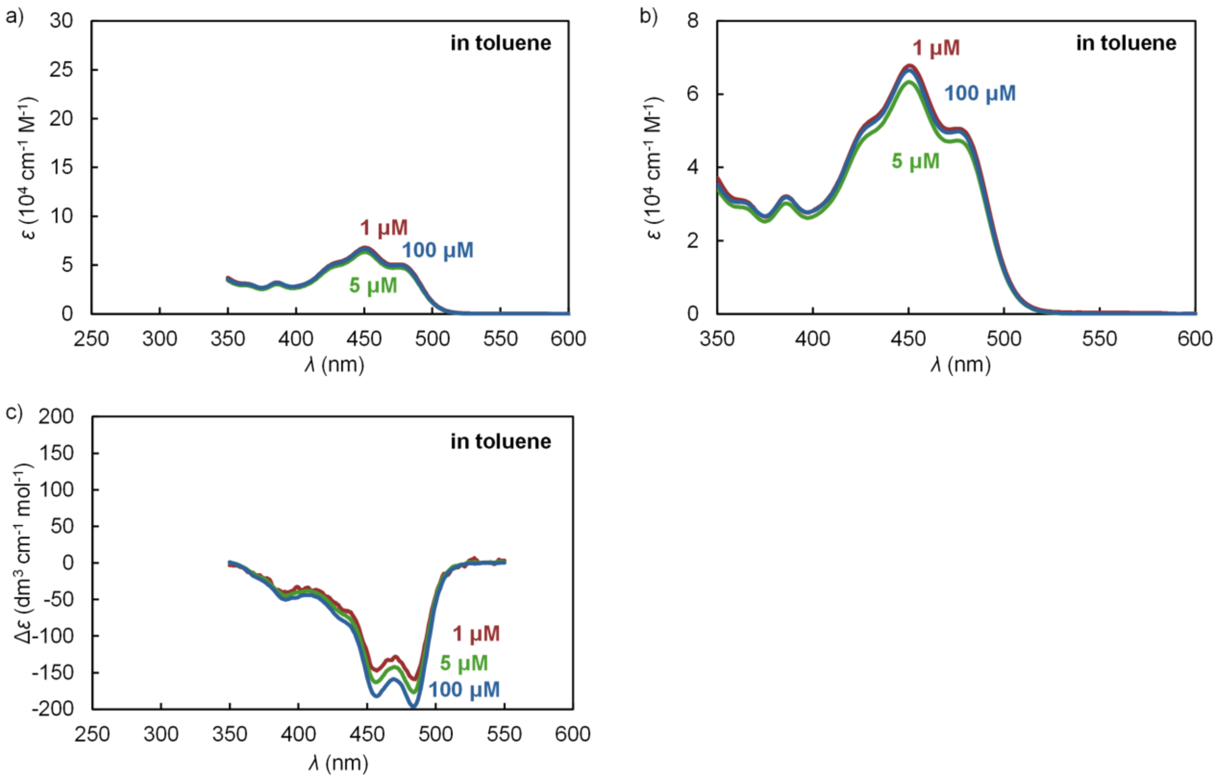

Figure S23. a, b) Variable-concentration UV-vis absorption and c) CD spectra of [Ag(**1c**)$_2$][PF_6] in toluene (r.t., [Ag(**1c**)$_2$][PF_6] = 1.0 µM (red line), 5.0 µM (green line), 100 µM (blue line)).

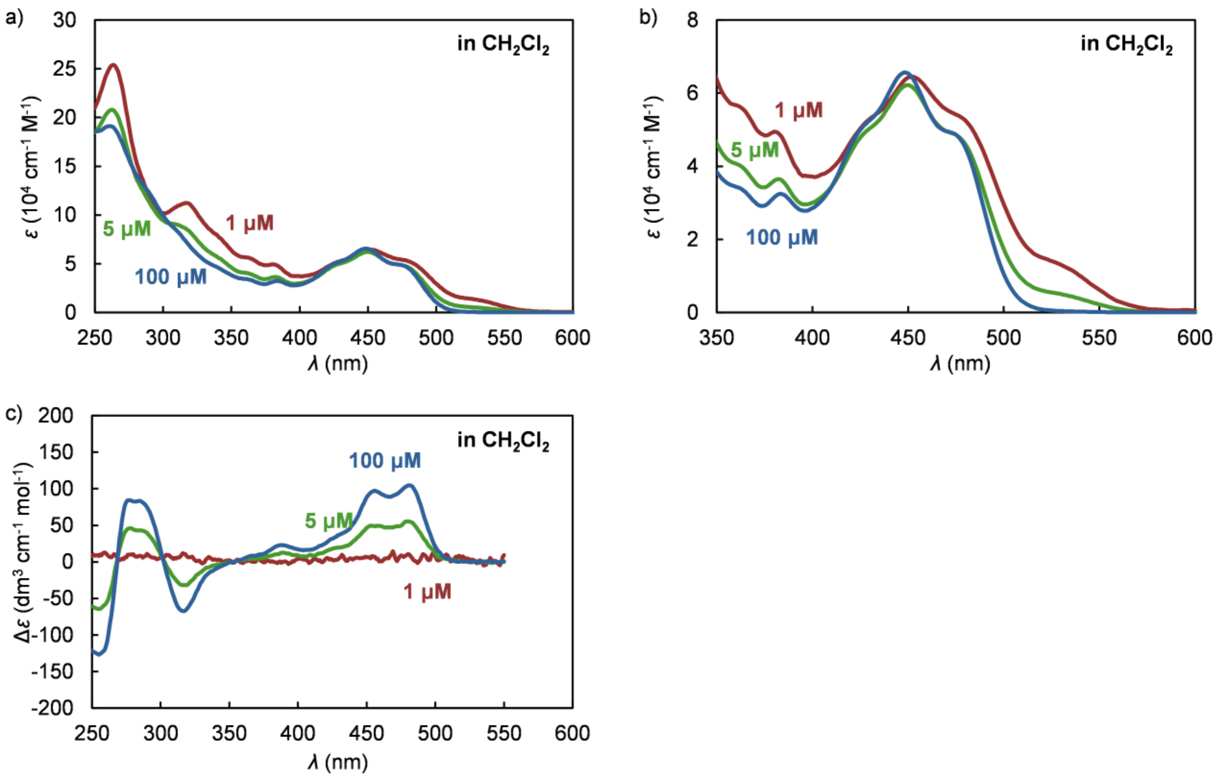

Figure S24. a, b) Variable-concentration UV-vis absorption and c) CD spectra of [Ag(**1c**)$_2$][PF_6] in CH_2Cl_2 (r.t., [Ag(**1c**)$_2$][PF_6] = 1.0 µM (red line), 5.0 µM (green line), 100 µM (blue line)).

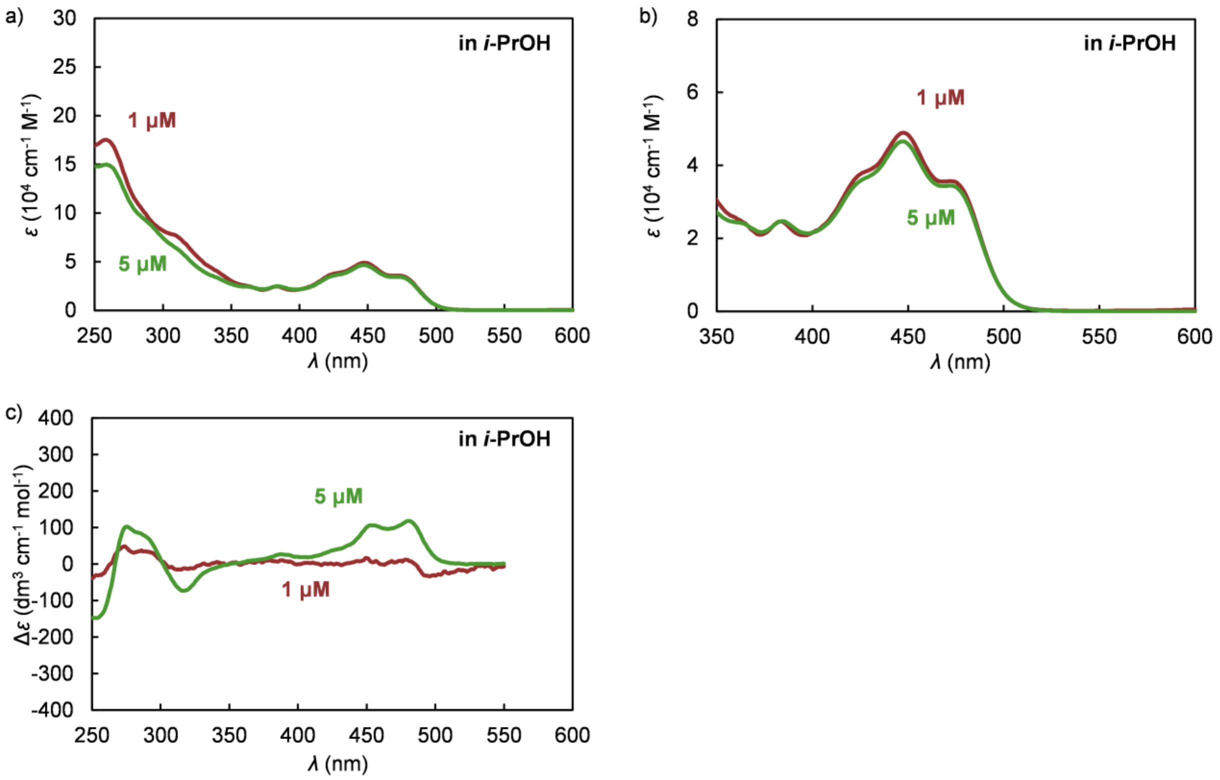

Figure S25. a, b) Variable-concentration UV-vis absorption and c) CD spectra of [Ag(**1c**)$_2$][PF$_6$] in *i*-PrOH (r.t., [Ag(**1c**)$_2$][PF$_6$] = 1.0 μM (red line) and 5.0 μM (green line)).

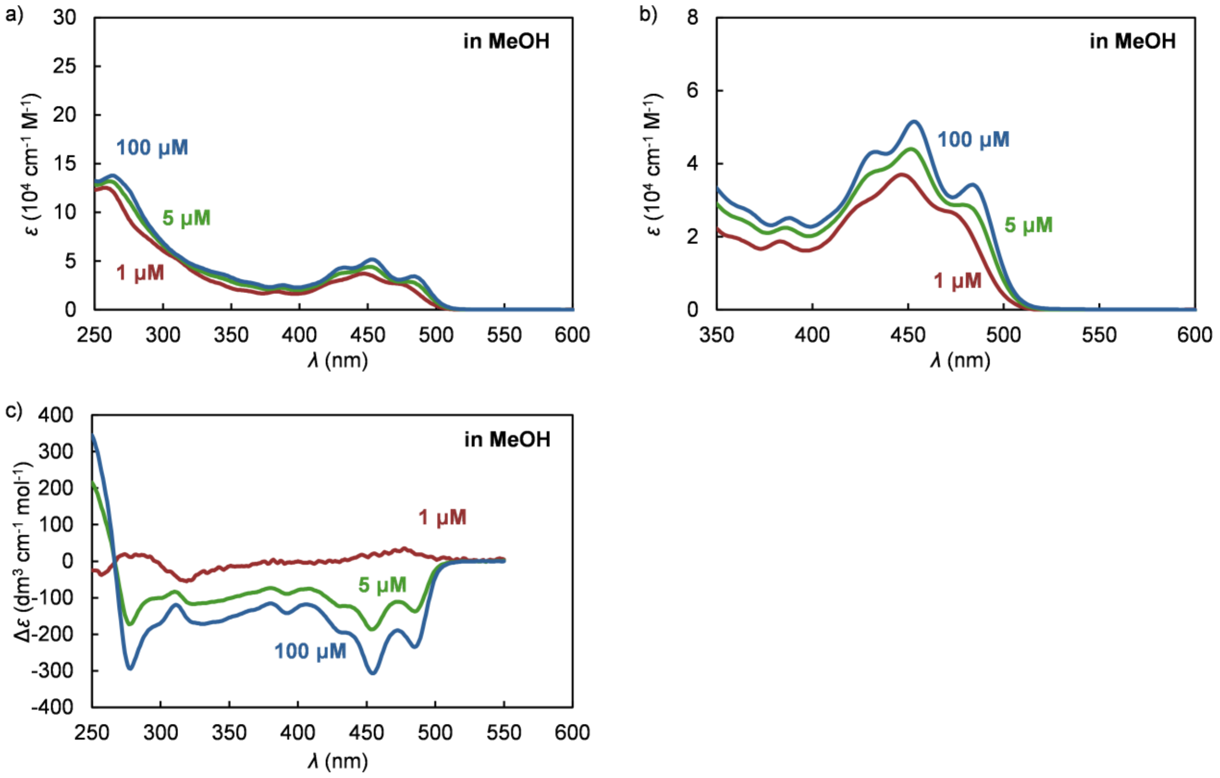

Figure S26. a, b) Variable-concentration UV-vis absorption and c) CD spectra of [Ag(**1c**)$_2$][PF$_6$] in MeOH (r.t., [Ag(**1c**)$_2$][PF$_6$] = 1.0 μM (red line), 5.0 μM (green line), 100 μM (blue line)).

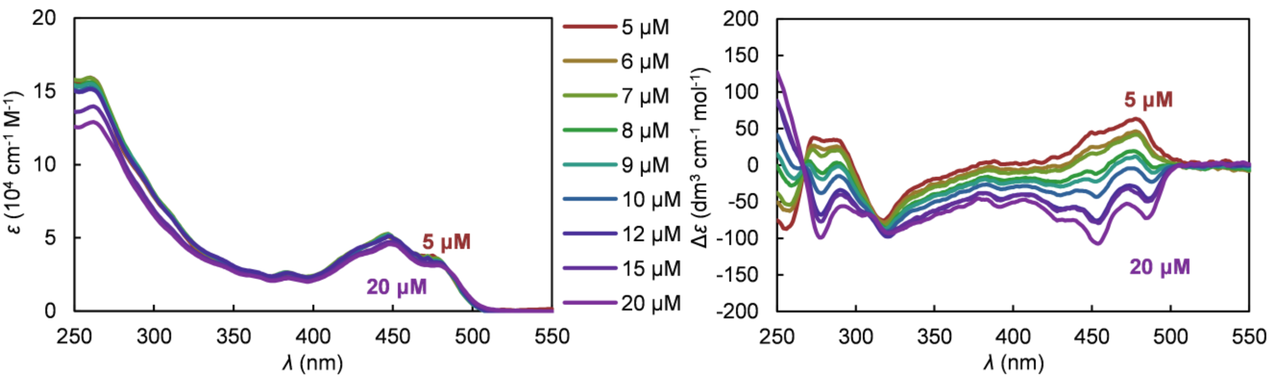

Figure S27. left) Variable-concentration UV-vis absorption and right) CD spectra of [Ag(**1c**)$_2$][PF_6] in *i*-PrOH/MeOH = 3:7 (30 °C, [Ag(**1c**)$_2$][PF_6] = 5.0–20 μM).

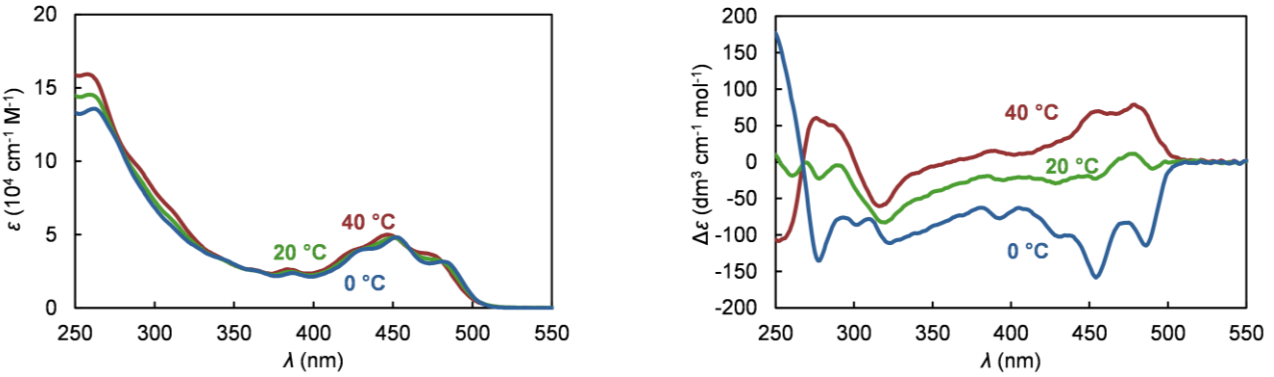

Figure S28. left) Variable-temperature UV-vis absorption and right) CD spectra of [Ag(**1c**)$_2$][PF_6] in *i*-PrOH/MeOH = 3:7 (40 °C (red line), 20 °C (green line), and 0 °C (blue line) at [Ag(**1c**)$_2$][PF_6] = 5.0 μM).

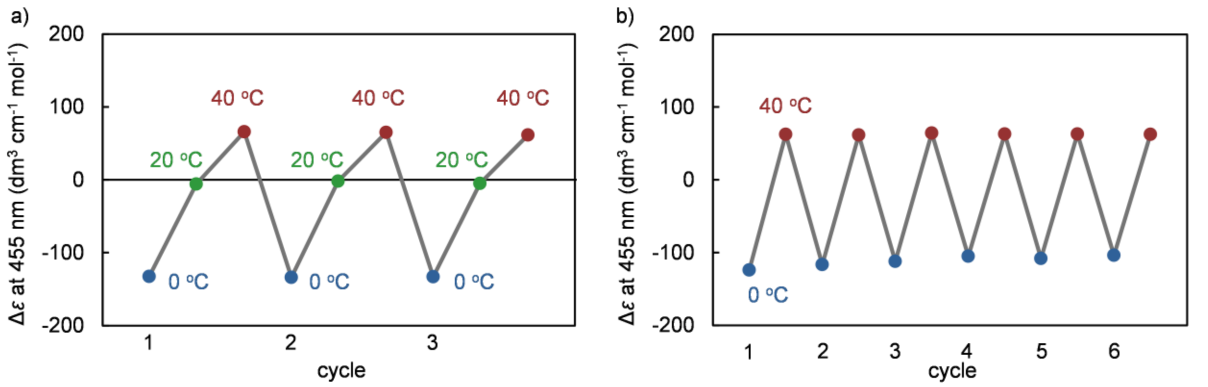

Figure S29. Temperature-regulated chiroptical switching cycles in [Ag(**1c**)$_2$][PF_6] (5.0 μM, *i*-PrOH/MeOH = 3:7). a) Three switching cycles among negative (0 °C), silent (20 °C) and positive Cotton effects (40 °C). b) Six switching cycles between negative (0 °C) and positive Cotton effects (40 °C).

UV-vis absorption and CD spectra of [Ag(1c)$_2$][PF$_6$] and [Zn(1c)$_2$][OTf]$_2$[S1]

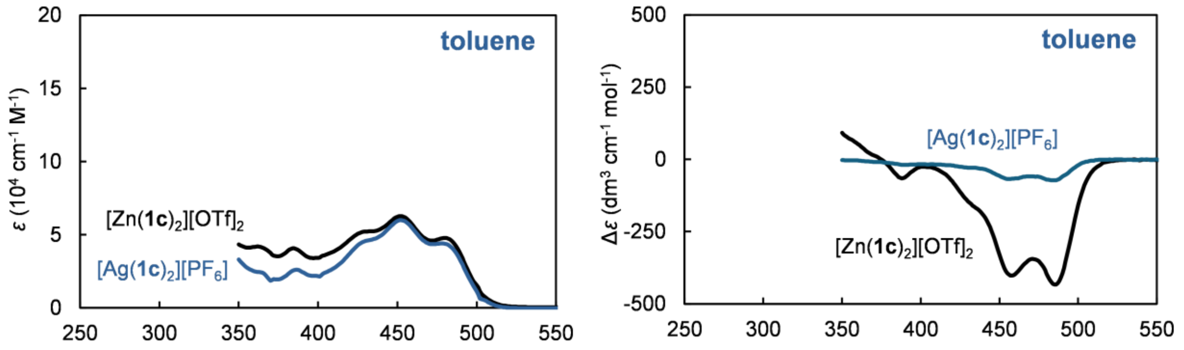

Figure S30. left) UV-vis absorption and right) CD spectra of [Ag(**1c**)$_2$][PF$_6$] and [Zn(**1c**)$_2$][OTf]$_2$[S1] in toluene (r.t., [Ag(**1c**)$_2$][PF$_6$] = [Zn(**1c**)$_2$][OTf]$_2$ = 5.0 μM).

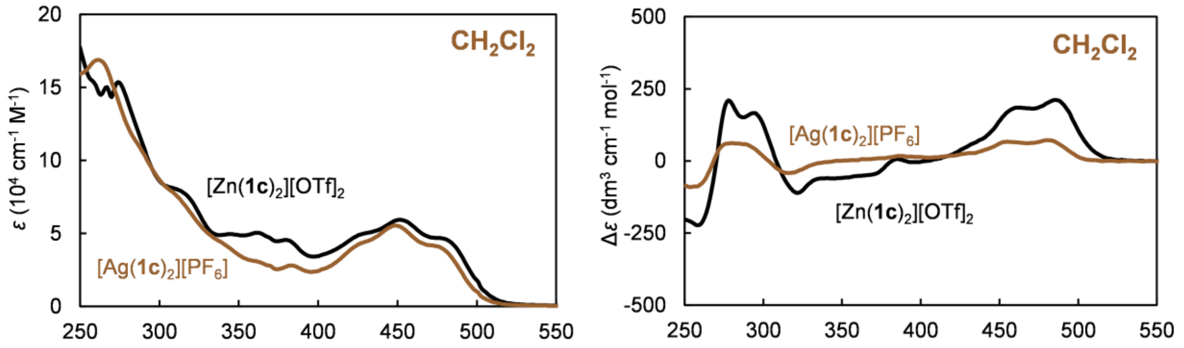

Figure S31. left) UV-vis absorption and right) CD spectra of [Ag(**1c**)$_2$][PF$_6$] and [Zn(**1c**)$_2$][OTf]$_2$[S1] in CH_2Cl_2 (r.t., [Ag(**1c**)$_2$][PF$_6$] = [Zn(**1c**)$_2$][OTf]$_2$ = 5.0 μM).

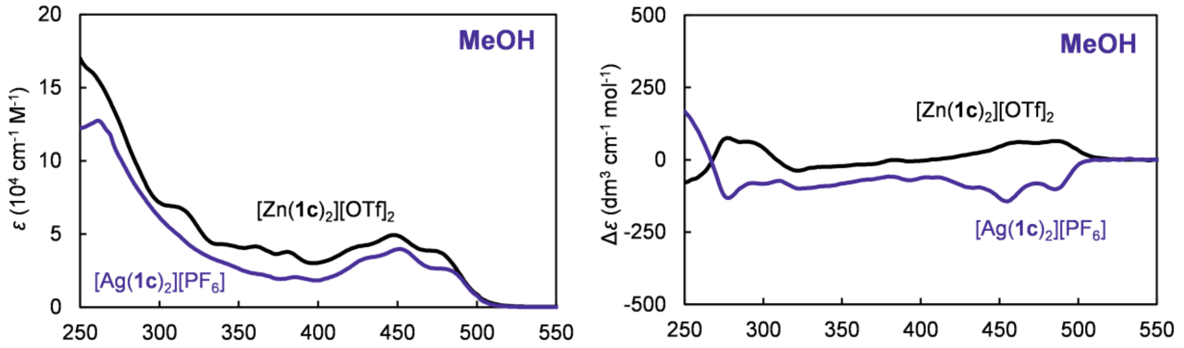

Figure S32. left) UV-vis absorption and right) CD spectra of [Ag(**1c**)$_2$][PF$_6$] and [Zn(**1c**)$_2$][OTf]$_2$[S1] in MeOH (r.t., [Ag(**1c**)$_2$][PF$_6$] = [Zn(**1c**)$_2$][OTf]$_2$ = 5.0 μM).

UV-vis absorption and CD spectra of [Ag(1c)$_2$][OTf]

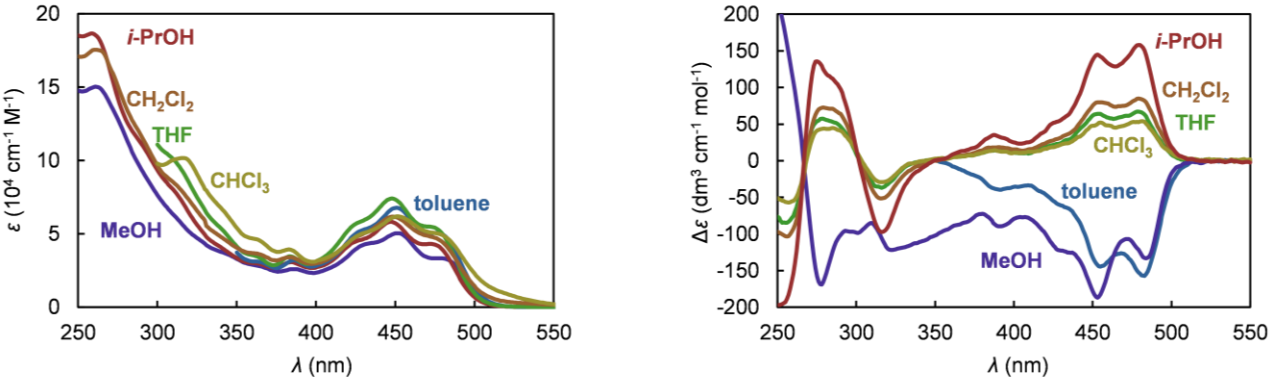

Figure S33. left) UV-vis absorption and right) CD spectra of [Ag(**1c**)$_2$][OTf] in *i*-PrOH (red line), CH_2Cl_2 (orange line), $CHCl_3$ (yellow line), THF (green line), toluene (blue line) and MeOH (purple line) (r.t., [Ag(**1c**)$_2$][PF_6] = 5.0 μM).

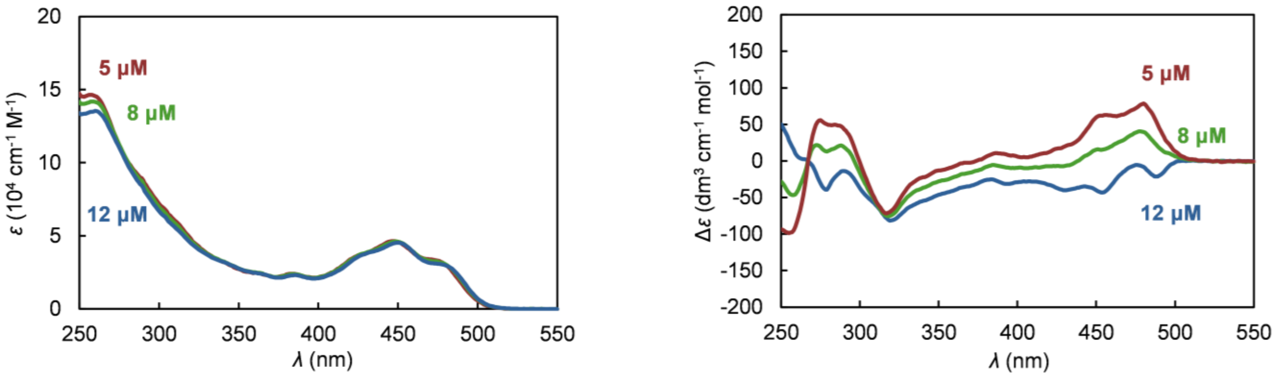

Figure S34. left) Variable-concentration UV-vis absorption (r.t., [Ag(**1c**)$_2$][OTf] = 12 μM (blue line), 8 μM (green line), 5.0 μM (red line)) and right) CD spectra of [Ag(**1c**)$_2$][OTf] in *i*-PrOH/MeOH = 3:7 (30 °C, [Ag(**1c**)$_2$][OTf] = 12 μM (blue line), 8 μM (green line), 5.0 μM (red line)).

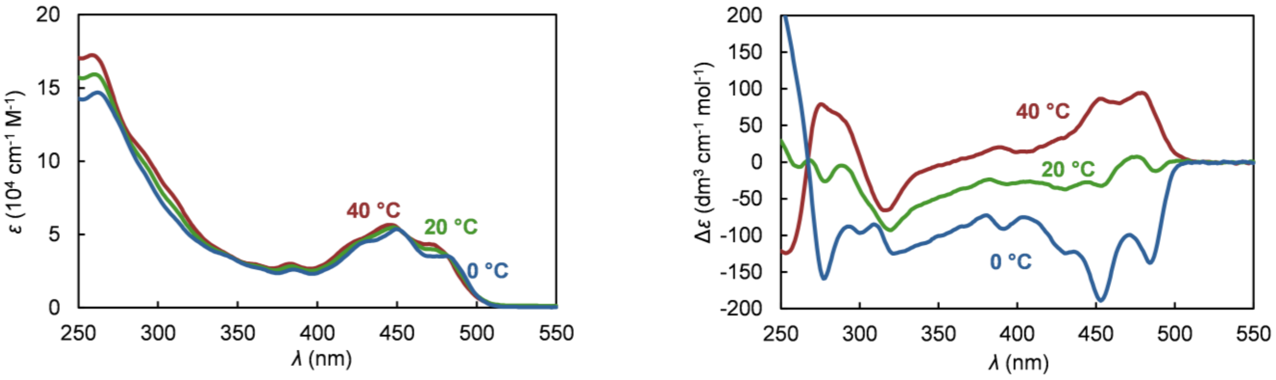

Figure S35. left) Variable-temperature UV-vis absorption and right) CD spectra of [Ag(**1c**)$_2$][OTf] in *i*-PrOH/MeOH = 3:7 (40 °C (red line), 20 °C (green line), and 0 °C (blue line) at [Ag(**1c**)$_2$][OTf] = 5.0 μM).

General procedure on UV-vis and CD measurements for [Zn(1c)$_2$][OTf]$_2$[S1]

To an NMR tube charged with **1c** (1.5 mg, 1.0 µmol) and $CDCl_3$ (0.50 mL) was added $Zn(OTf)_2$ in acetone-d_6 (50 mM, 10 µL, 0.5 µmol, 0.5*eq.*) and MS3A (10 mg). After the mixture was stirred for 1 min, the formation of complex [Zn(**1c**)$_2$][OTf]$_2$ was confirmed by ^{1}H NMR spectroscopy. The solution was concentrated and dried in *vacuo* to obtained [Zn(**1c**)$_2$][OTf]$_2$ complex as a yellow solid. [Zn(**1c**)$_2$][OTf]$_2$ was dissolved in solvents for UV-vis absorption and CD spectra measurements.

General procedure on UV-vis and CD measurements for [Cu(1c)$_2$][OTf]$_2$

To an NMR tube charged with **1c** (1.5 mg, 1.0 µmol) and $CHCl_3$ (0.50 mL) was added $Cu(OTf)_2$ in acetone (50 mM, 10 µL, 0.5 µmol, 0.5*eq.*). After the mixture was stirred for 1 min. The solution was concentrated and dried in *vacuo* to obtained [Cu(**1c**)$_2$][OTf]$_2$ complex as a brown solid. [Cu(**1c**)$_2$][OTf]$_2$ was dissolved in solvents for UV-vis absorption and CD spectra measurements.

General procedure on UV-vis and CD measurements for [Cu(1c)$_2$][PF$_6$]

To an NMR tube charged with **1c** (1.5 mg, 1.0 µmol) and $CHCl_3$ (0.50 mL) was added [Cu(MeCN)$_4$][PF$_6$] in MeCN (50 mM, 10 µL, 0.5 µmol, 0.5*eq.*). After the mixture was stirred for 1 min. The solution was concentrated and dried in *vacuo* to obtained [Cu(**1c**)$_2$][PF$_6$] complex as a brown solid. [Cu(**1c**)$_2$][PF$_6$] was dissolved in solvents for UV-vis absorption and CD spectra measurements.

UV-vis absorption and CD spectra of [Zn(1c)$_2$][OTf]$_2$

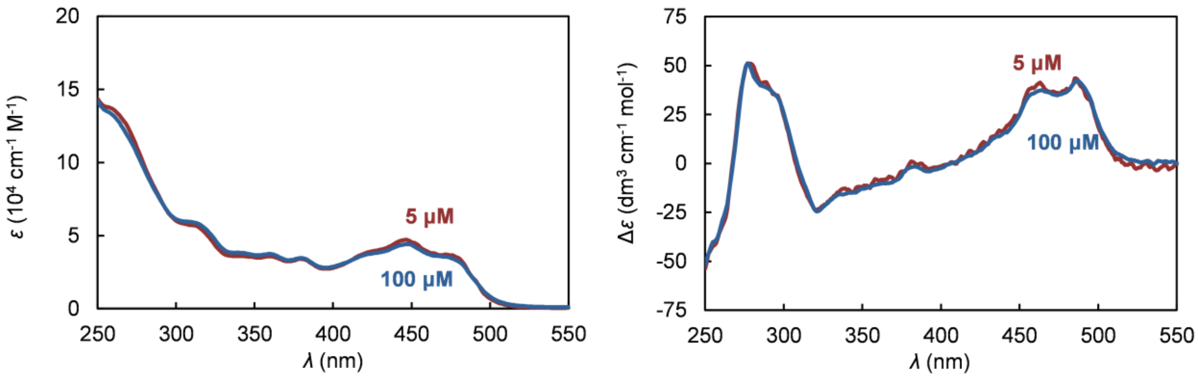

Figure S36. left) Variable-concentration UV-vis absorption and right) CD spectra of [Zn(**1c**)$_2$][OTf]$_2$ in *i*-PrOH/MeOH = 3:7 (30 °C, [Zn(**1c**)$_2$][OTf]$_2$ = 5 µM (red line), 100 µM (blue line)).

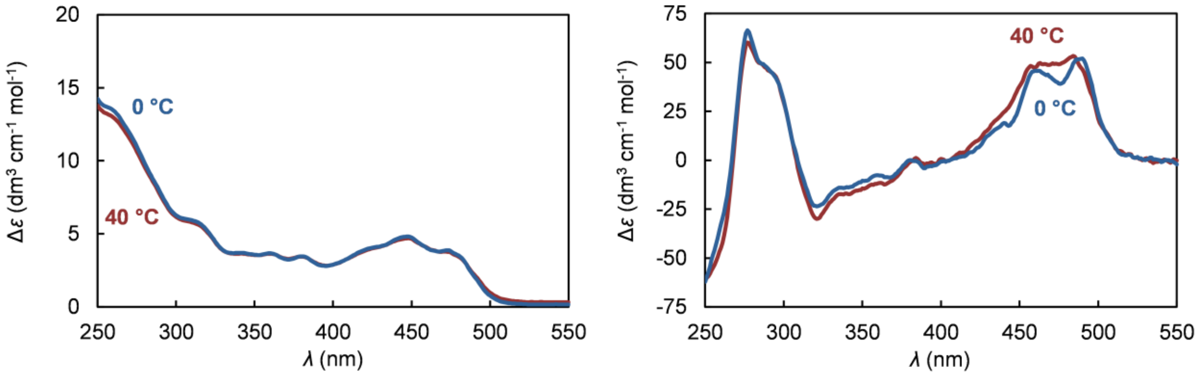

Figure S37. left) Variable-temperature UV-vis absorption and right) CD spectra of [Zn(**1c**)$_2$][OTf]$_2$ in *i*-PrOH/MeOH = 3:7 (0°C (blue line), and 40 °C (red line) at [Zn(**1c**)$_2$][OTf]$_2$ = 5 µM).

CD measurements revealed that the Cu(I) and Cu(II) based foldamers [Cu(**1c**)$_2$][PF_6] and [Cu(**1c**)$_2$][OTf]$_2$ exhibited inversion of the Cotton effects depending on the solvent, which was similar to that observed for [Zn(**1c**)$_2$][OTf]$_2$ and [Ag(**1c**)$_2$][PF_6] (Figure S38). The Cu(I) based complex [Cu(**1c**)$_2$][PF_6] exhibited trends similar to those of the Ag(I)-based complex [Ag(**1c**)$_2$][PF_6], showing a negative Cotton effect in toluene and positive Cotton effects in CH_2Cl_2, $CHCl_3$, and THF for the range of 400–500 nm (Figure S38b). In MeOH and *i*-PrOH, [Cu(**1c**)$_2$][PF_6] exhibited larger, broader, and more extended negative Cotton effects around 350–450 nm, which suggested the formation of aggregates. In contrast, the Cu(II) based complex [Cu(**1c**)$_2$][OTf]$_2$ exhibited a negative Cotton effect in toluene, a positive Cotton effect in CH_2Cl_2, and smaller Cotton effects in MeOH, *i*-PrOH, $CHCl_3$, and THF for the range of 400–500 nm, similar to those observed for [Zn(**1c**)$_2$][OTf]$_2$ (Figure S38d).

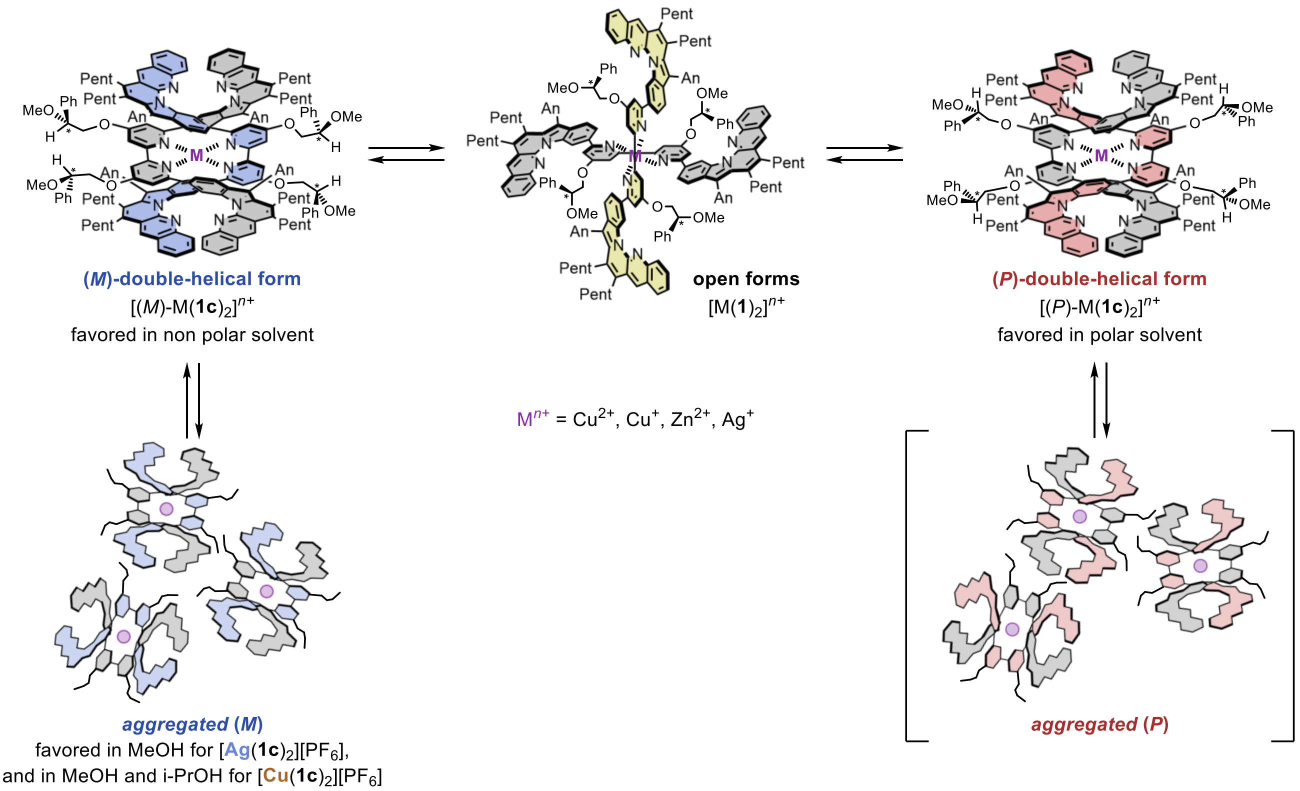

Scheme S9. *M*/*P* helicity switching in double-helical monometallofoldamers [M(**1c**)]$^{n+}$.

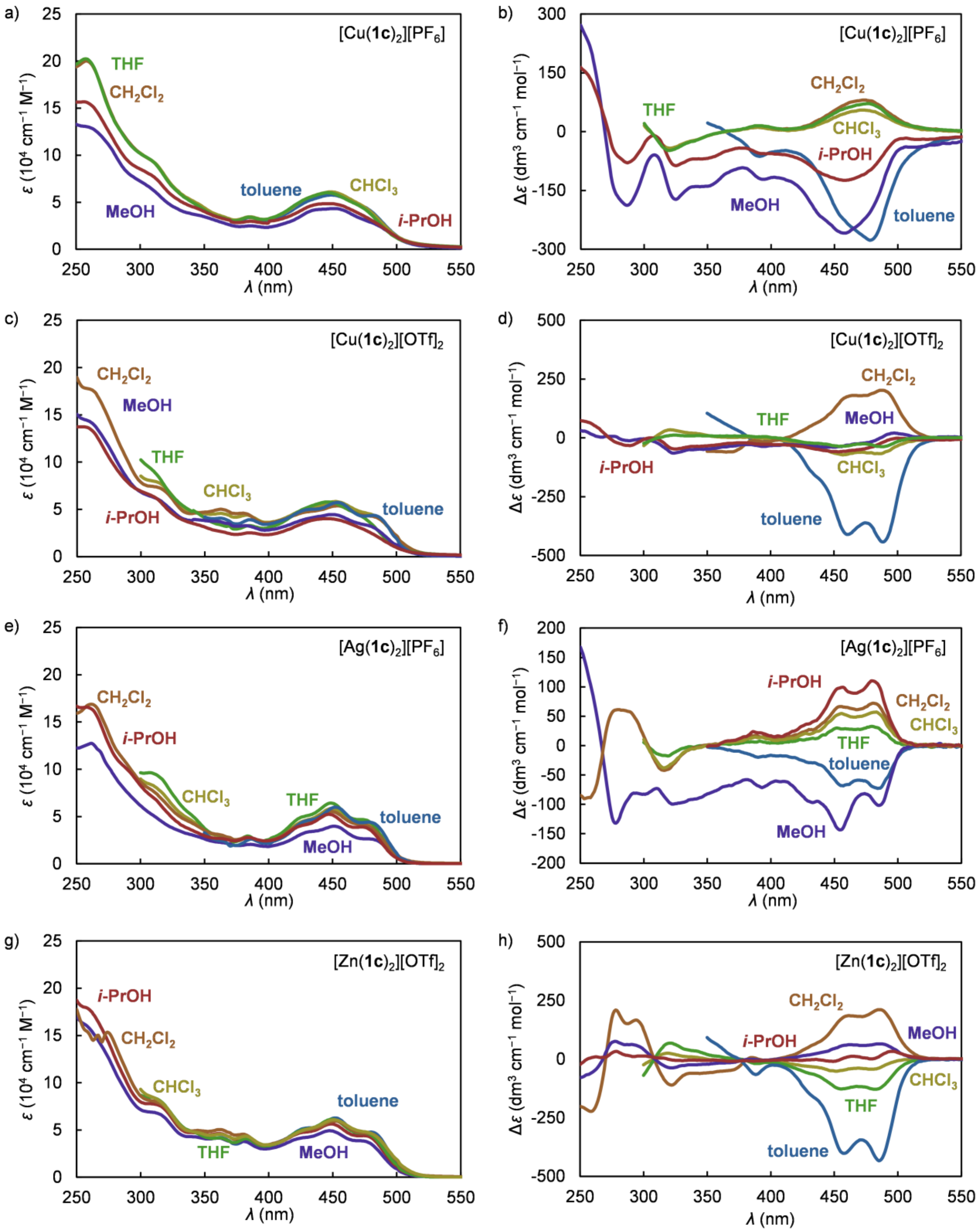

Figure S38. UV-vis absorption and CD spectra of a, b) [Cu(**1c**)$_2$][PF$_6$], c, d) [Cu(**1c**)$_2$][OTf]$_2$, e, f) [Ag(**1c**)$_2$][OTf] and g, h) [Zn(**1c**)$_2$][OTf]$_2$ in *i*-PrOH (red line), CH_2Cl_2 (orange line), $CHCl_3$ (yellow line), THF (green line), toluene (blue line) and MeOH (purple line) (r.t., [compound] = 5.0 μM).

We previously reported that conformational change in the chiral side chains was key to the helicity inversion of the metallofoldamer.[S1] The conformations of these chiral side chains were found to differ markedly between polar solvents, which favor (*P*)-helicity, and non-polar solvents, which favor (*M*)-helicity. We therefore propose that this conformational change is driven by differences in the interactions between the double helix and the counterions (PF_6^-), which dictates helicity preference. In non-polar solvents, the (*M*)-helicity is likely stabilized through interactions between the counterions and the double-helix, whereas in polar solvents, the suppression of these interactions shifts the preference toward the (*P*)-helicity (Scheme S10).

In our previously reported Zn(II) complex,[S1] ROESY measurements in toluene, (where the (*M*)-helicity was dominant), revealed that the O–CH_2–CHPh–OMe moiety of the chiral side chains adopted a gauche conformation (Figure S30). This observation suggests that the thermodynamically less favorable gauche form is stabilized through CH···F or CH···O electrostatic interactions between the counterion and the chiral side chains. Conversely, in polar solvents such as MeOH or *i*-PrOH, the interactions between the double-helical Zn(II) complex and the counterions are weakened thereby favoring the (*P*)-helicity. Consistent with this hypothesis, CD measurements confirmed that the (*P*)-helicity was favored in these polar solvents (Figure S32, 33).

In contrast to the dispersed state, where solvent molecules effectively surrounded the counterions, aggregation necessitates partial desolvation of the complex, thereby enhancing these interactions with counterions and stabilizing the (*M*)-helicity even in polar solvents. We therefore propose that the concentration- and temperature-induced changes in the equilibrium between the aggregated and dispersed state in polar solvents are the driving force for the observed chiroptical inversion of [Ag(**1c**)$_2$][PF_6].

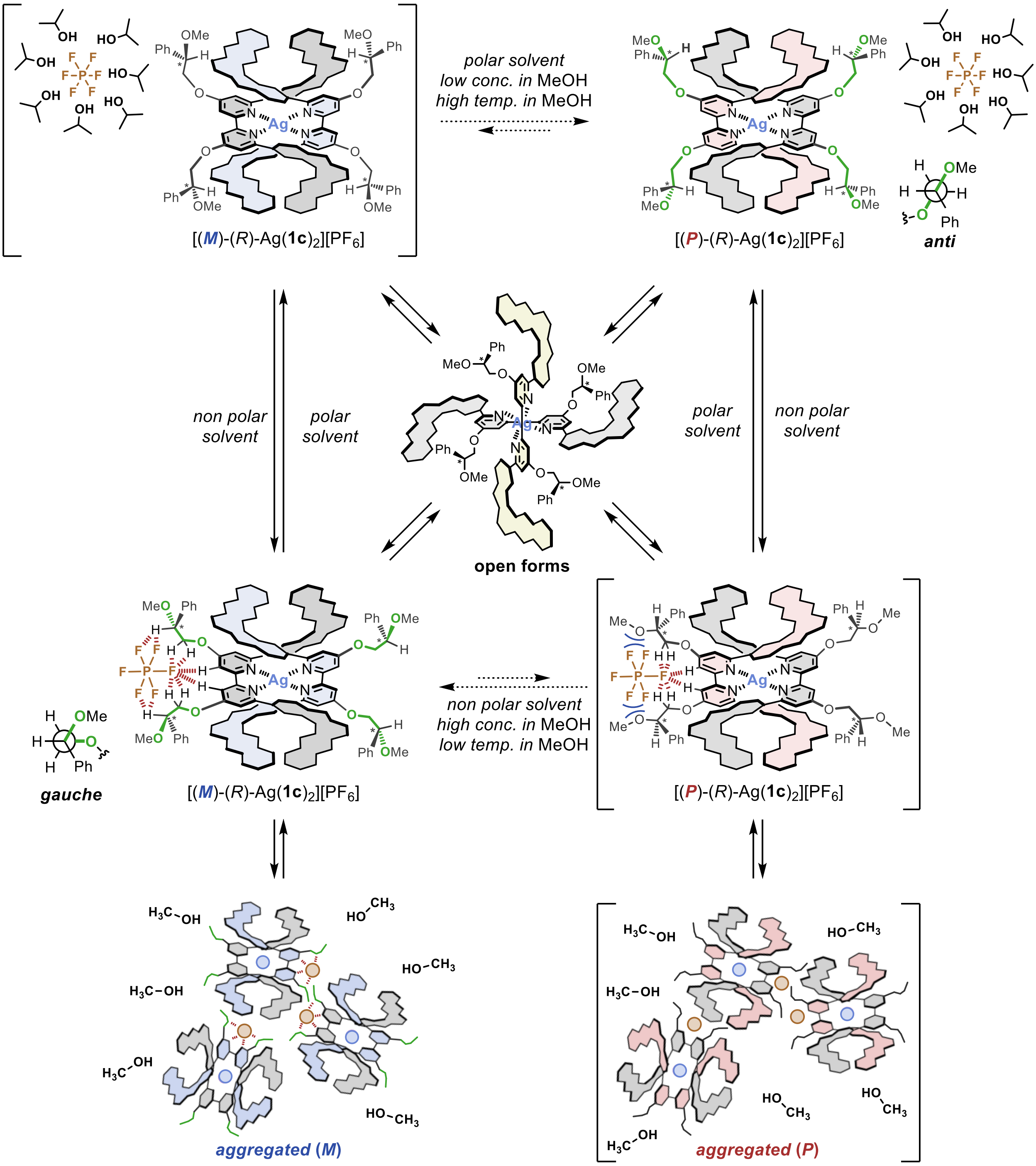

Scheme S10. Proposed solvent-dependent conformational change of the chiral side chains and helicity preference of [Ag(**1c**)$_2$][PF$_6$].

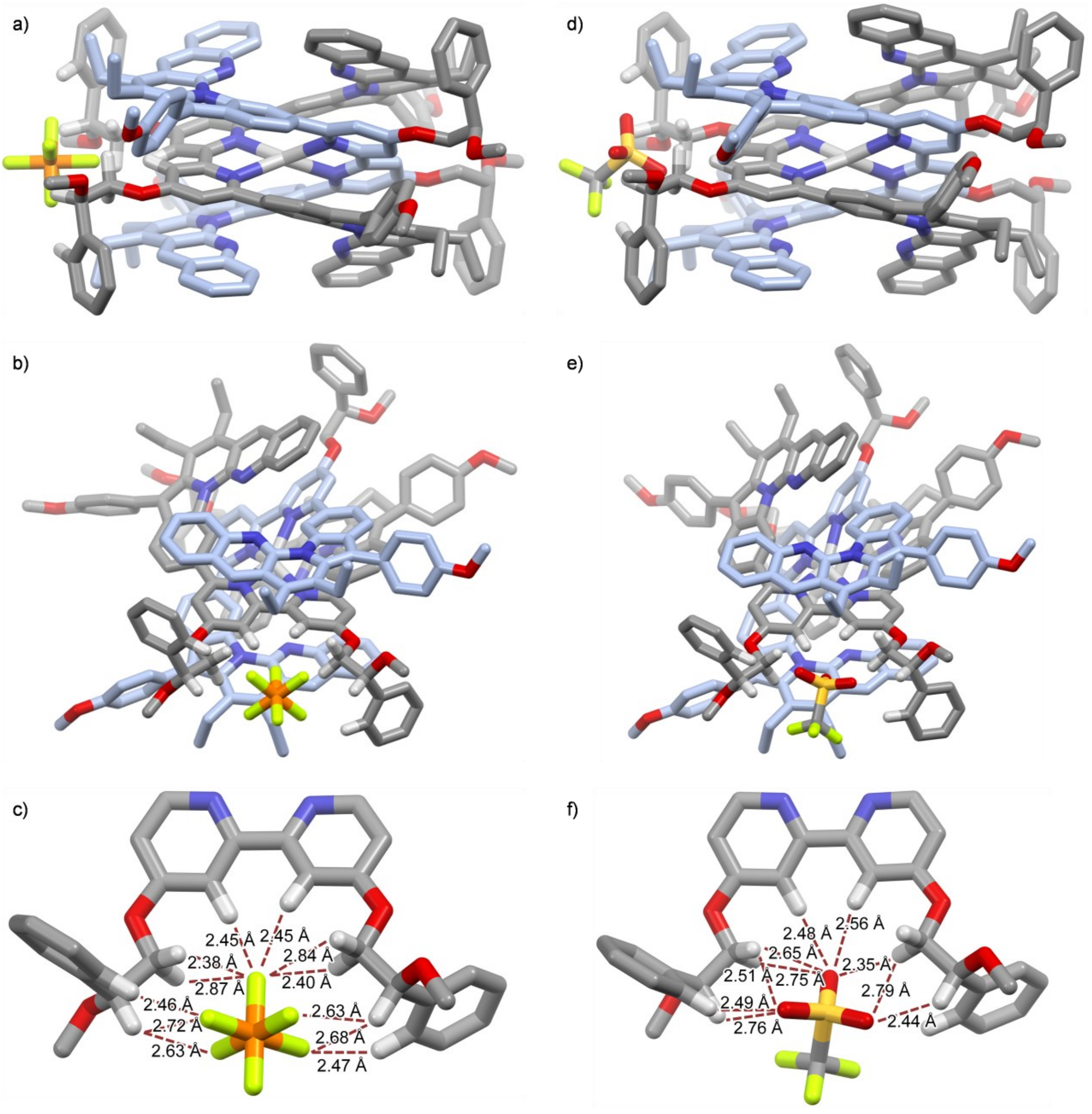

[(*M*)-Ag(**1c'**)$_2$][PF$_6$] [(*M*)-Ag(**1c'**)$_2$][OTf]

Figure S39. DFT-optimized structures of a−c) [Ag(**1c'**)$_2$][PF$_6$] and d−f) [Ag(**1c'**)$_2$][OTf], computed at the r^{2}SCAN-3c/def2-mTZVPP level[S6], using ethyl groups in place of pentyl groups.

7. Luminescence and CPL spectra

General procedure on luminescence and CPL spectra for [Ag(1c)$_2$][PF$_6$]

To an NMR tube charged with **1c** (1.5 mg, 1.0 µmol) and $CHCl_3$ (0.50 mL) was added $AgPF_6$ in CH_3CN (50 mM, 10 µL, 0.5 µmol, 0.5.*eq*.). After the mixture was stirred for 1 min, the formation of complex [Ag(**1c**)$_2$][PF$_6$] was confirmed by ^{1}H NMR spectroscopy (See section 2). The solution of [Ag(**1c**)$_2$][PF$_6$] was concentrated and dried in *vacuo* to obtain [Ag(**1c**)$_2$][PF$_6$] as a yellow solid. [Ag(**1c**)$_2$][PF$_6$] was dissolved in various solvent for luminescence and CPL spectra measurements. For solid-state measurements, powdered samples were placed between two KBr plates (5×5×1 mm) and mounted on a KBr plate sample holder.

Luminescence spectra of 1c and [Ag(1c)$_2$][PF$_6$]

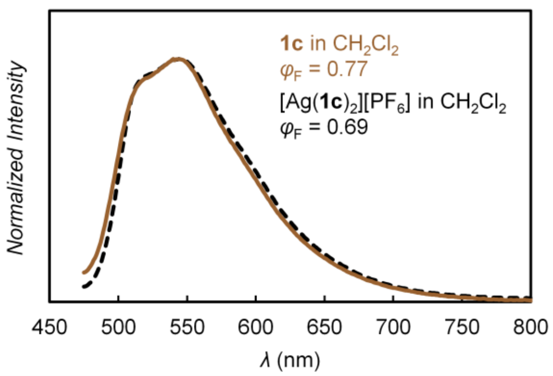

Figure S40. Fluorescence spectra of **1c** (λ_{ex} = 450 nm, [**1c**] = 10 µM) and [Ag(**1c**)$_2$][PF$_6$] (λ_{ex} = 440 nm, [Ag(**1c**)$_2$][PF$_6$] = 5.0 µM) in CH_2Cl_2.

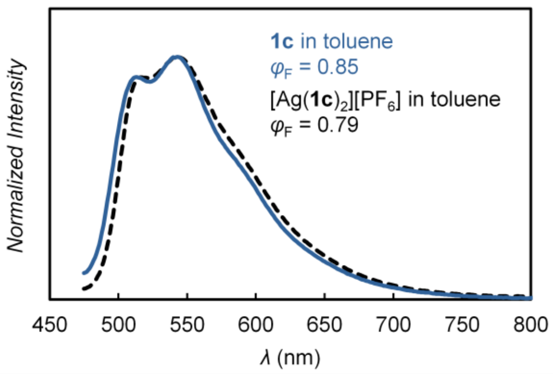

Figure S41. Fluorescence spectra of **1c** (λ_{ex} = 450 nm, [**1c**] = 10 µM) and [Ag(**1c**)$_2$][PF$_6$] (λ_{ex} = 440 nm, [Ag(**1c**)$_2$][PF$_6$] = 5.0 µM) in toluene.

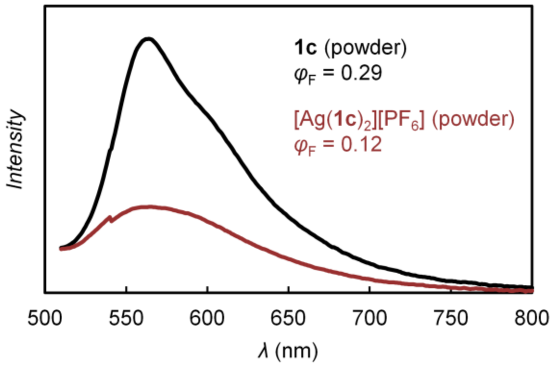

Figure S42. Fluorescence spectra of **1c** and [Ag(**1c**)$_2$][PF$_6$] as powder on KBr plates (λ_{ex} = 440 nm).

Luminescence spectra of [Ag(1c)$_2$][PF$_6$]

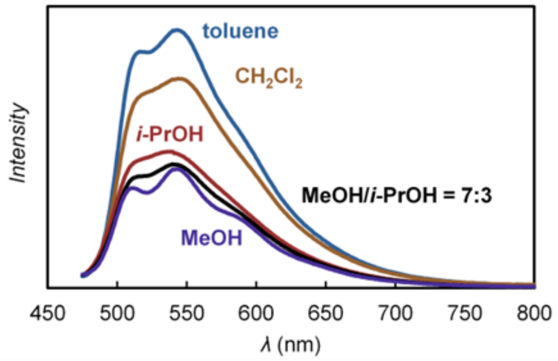

Figure S43. Fluorescence spectra of [Ag(**1c**)$_2$][PF$_6$] (λ_{ex} = 440 nm, [Ag(**1c**)$_2$][PF$_6$] = 5 μM) in toluene (blue line), CH_2Cl_2 (orange line), *i*-PrOH (red line), MeOH (purple line) and MeOH/*i*-PrOH = 7:3 (black line).

Table S8. Fluorescence data for [Ag(**1c**)$_2$][PF$_6$].

solvent	λ_{em} (nm)[a]	φ_F
toluene	517, 543	0.79
CH_2Cl_2	544	0.69
i-PrOH	537	0.46
MeOH	512, 543	0.42
MeOH/*i*-PrOH = 7:3	539	0.43

[a] λ_{ex} = 450 nm, [Ag(**1c**)$_2$][PF$_6$] = 5.0 μM.

CPL spectra of 1c and [Ag(1c)$_2$][PF$_6$]

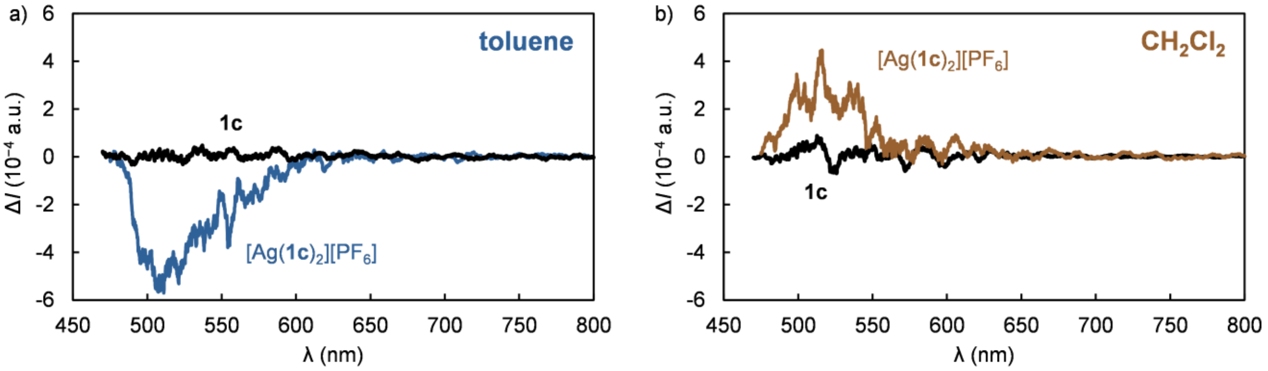

Figure S44. CPL spectra of **1c** and [Ag(**1c**)$_2$][PF$_6$] in a) toluene and b) CH_2Cl_2 (r.t., [**1c**] = 10 μM, [Ag(**1c**)$_2$][PF$_6$] = 5.0 μM).

CPL spectra of [Ag(1c)$_2$][PF$_6$]

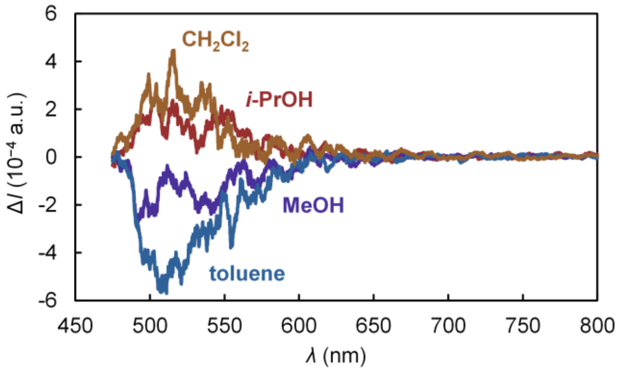

Figure S45. CPL spectra of [Ag(**1c**)$_2$][PF$_6$] (λ_{ex} = 440 nm, [Ag(**1c**)$_2$][PF$_6$] = 5.0 μM) in CH_2Cl_2 (orange line), *i*-PrOH (red line), MeOH (purple line) and toluene (blue line).

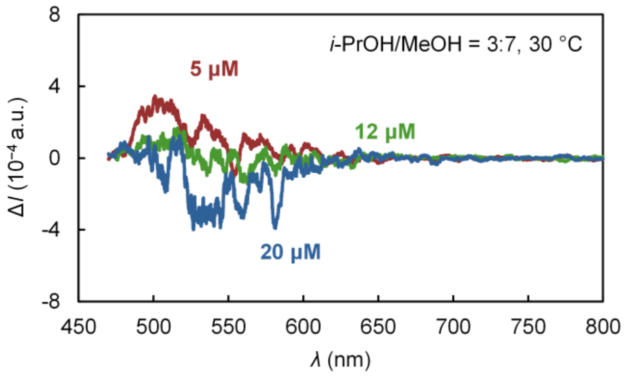

Figure S46. Variable-concentration CPL spectra of [Ag(**1c**)$_2$][PF_6] in *i*-PrOH/MeOH = 3:7 (30 °C, [Ag(**1c**)$_2$][PF_6] = 5.0–20 µM).

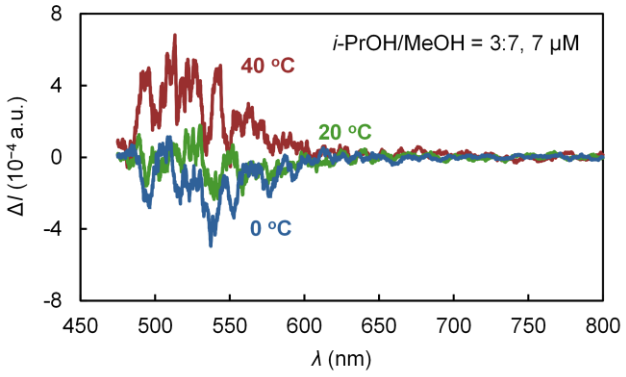

Figure S47. Variable-temperature CPL spectra of [Ag(**1c**)$_2$][PF_6] in *i*-PrOH/MeOH = 3:7 (0–40 °C, [Ag(**1c**)$_2$][PF_6] = 7.0 µM).

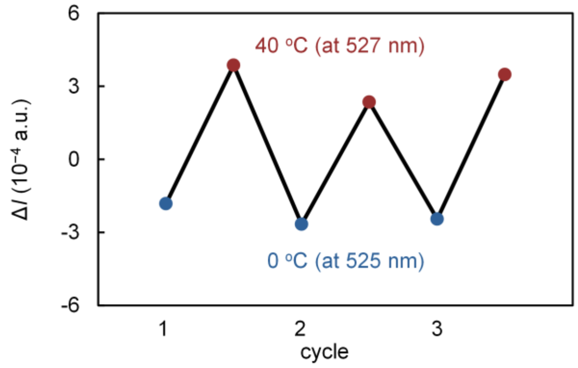

Figure S48. Temperature-regulated chiroptical switching cycles in [Ag(**1c**)$_2$][PF_6] (7.0 µM, *i*-PrOH/MeOH = 3:7) between negative (0 °C) and positive CPL signals (40 °C).

8. DLS measurements

General procedure on DLS measurements for [Ag(1c)$_2$][PF$_6$]

To a screw bottle charged with **1c** (5.9 mg, 4.0 μmol) and $CHCl_3$ (0.5 mL) was added $AgPF_6$ in CH_3CN (50 mM, 40 μL, 2 μmol, 0.5 *eq.*). After the mixture was stirred for 1 min, the solution of [Ag(**1c**)$_2$][PF$_6$] was concentrated and dried in *vacuo* to obtain [Ag(**1c**)$_2$][PF$_6$] as a yellow solid. [Ag(**1c**)$_2$][PF$_6$] was dissolved in MeOH to a concentration of 0.2 mM for DLS measurements.

DLS analysis of [Ag(**1c**)$_2$][PF$_6$] in MeOH indicated the formation of aggregates with a very broad particle size distribution (Figure S24). The volume distribution suggested that particles ranging from 2 to 10 nm, with a maximum approximately 5 nm, composed more than 99% of the overall population (Figure S24b). On the other hand, in toluene and CH_2Cl_2, the scattered light of [Ag(**1c**)$_2$][PF$_6$] could not be analyzed due to the small particle size.

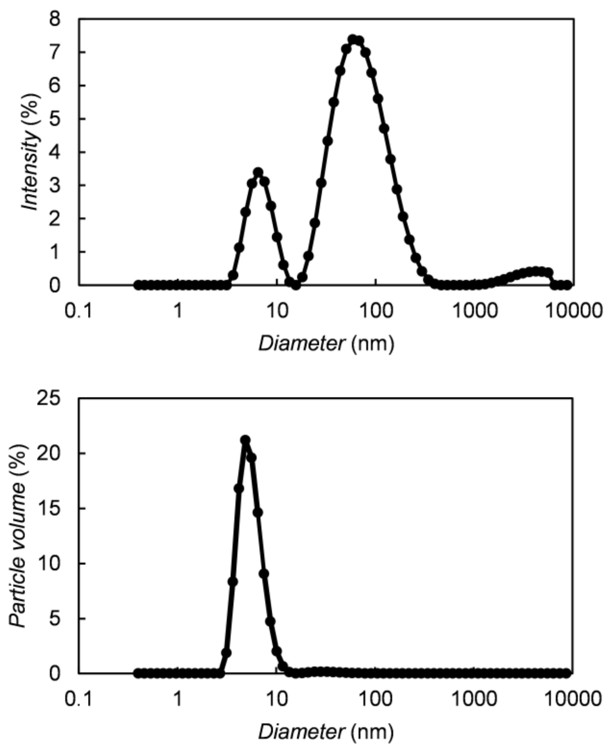

Figure S49. a) DLS intensity distribution and b) volume distribution of [Ag(**1c**)$_2$][PF$_6$] in MeOH (0.20 mM).

9. Theoretical calculations

General

All DFT and TD-DFT calculations were carried out with ORCA version 5.0.4.[S3-5] Geometry optimization and TD-DFT calculations were carried out at the revPBE0-D3(BJ)/def2-SVP (for C, H, N, O), def2-TZVP (for Ag) level of theory with the resolution of RIJCOSX approximation using the def2/ auxiliary basis set. TD-DFT calculations were performed using the Tamm-Dancoff approximation.

TD-DFT calculations for the model complex [(*M*)-Ag(**1d**)$_2$]$^+$ (with methoxy groups instead of (*R*)-2-methoxy-2-phenylethoxy groups and methyl groups instead of pentyl groups) attributed the negative Cotton effect to the (*M*)-double helix (Figure S50).

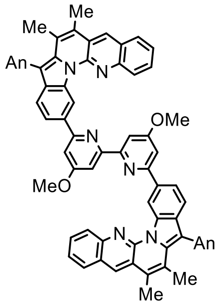

1d

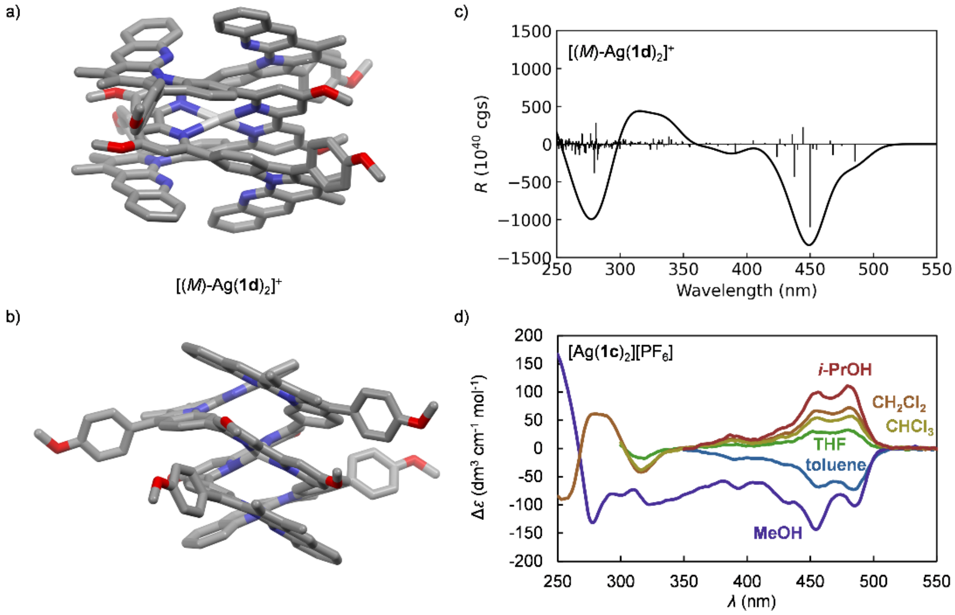

Figure S50. a, b) Optimized structures and c) CD spectra of [(*M*)-Ag(**1d**)$_2$]$^+$ calculated by TD-DFT/TDA (Orca 5.0.4, revPBE0-D3(BJ)/def2-SVP for C, H, N, O, def2-TZVP for Ag). d) CD spectra of [Ag(**1c**)$_2$][PF_6] (r.t., [Ag(**1c**)$_2$][PF_6] = 5.0 μM).

10. Cyclic voltammogram of the Cu(I)-based monometallofoldamer

In order to investigate the electrochemical properties of [Cu(**1**)$_2$]$^{n+}$ (n = 1 or 2), cyclic voltammetry (CV) of [Cu(**1b**)$_2$][PF_6] was measured (Figure S38). The cyclic voltammogram of [Cu(**1b**)$_2$][PF_6] showed the reversible redox wave with $E_{1/2}$ = −0.09 V (E_{pa} = −0.01 V, E_{pc} = −0.17 V) vs. Fc/Fc$^+$, which was considered to correspond to the Cu(I)/Cu(II) redox couple.

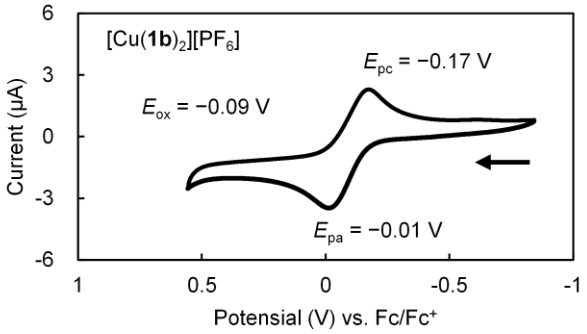

Figure S51. Cyclic voltammogram of [Cu(**1c**)$_2$][PF_6] (CH_2Cl_2, [Cu(**1c**)$_2$][PF_6] = 1.0 mM, electrolyte: 0.1 M $TBAPF_6$, scan rate: 100 mV s^{-1}, r.t.) (E_{pa}: anodic peak potential, E_{pc}: cathodic peak potential).

11. References

(S1) Matsumura, K.; Kinjo, K.; Tateno, K.; Ono, K.; Tsuchido, Y.; Kawai, H. *M*/*P* Helicity Switching and Chiral Amplification in Double-Helical Monometallofoldamers. *J. Am. Chem. Soc.* **2024**, *146*, 21078–21088. https://doi.org/10.1021/jacs.4c06560.

(S2) Bannwarth, C.; Ehlert, S.; Grimme, S. GFN2-xTB—An Accurate and Broadly Parametrized Self-Consistent Tight-Binding Quantum Chemical Method with Multipole Electrostatics and Density-Dependent Dispersion Contributions. *J. Chem. Theory Comput.* **2019**, 15, 1652–1671. https://doi.org/10.1021/acs.jctc.8b01176

(S3) Neese, F. The ORCA program system. *WIREs Comput Mol Sci.* **2012**, *2*, 73–78. https://doi.org/10.1002/wcms.81

(S4) Wennmohs, F; Becker, U; Riplimger, C. The ORCA quantum chemistry program package. *J. Chem. Phys*. **2020**, *152*, 224108. https://doi.org/10.1063/5.0004608

(S5) Neese, F. Software update: The ORCA program system-Version 5.0. *WIREs Comput Mol Sci*. **2022**, *12*, e1606. https://doi.org/10.1002/wcms.1606

(S6) Grimm, S.; Hansen, A.; Ehlert, S.; Mewes, J-M. r^{2}SCAN-3c: A "Swiss army knife" composite electronic-structure method. *J. Chem. Phys*. **2021**, *154*, 064103. https://doi.org/10.1063/5.0040021
